# Synthesis and Catalytic Applications of Advanced Sn‐ and Zr‐Zeolites Materials

**DOI:** 10.1002/advs.202306533

**Published:** 2023-12-26

**Authors:** Xue Liu, Zhiguo Zhu

**Affiliations:** ^1^ Department of Chemistry College of Science Hebei Agricultural University Lingyusi Road 289 Baoding 071001 P. R. China; ^2^ College of Chemistry and Chemical Engineering Yantai University Qingquan Road 30 Yantai 264005 P. R. China

**Keywords:** biomass conversion, Lewis acid catalysis, preparation approaches, stannosilicates, zirconosilicates

## Abstract

The incorporation of isolated Sn (IV) and Zr (IV) ions into silica frameworks is attracting widespread attention, which exhibits remarkable catalytic performance (conversion, selectivity, and stability) in a broad range of reactions, especially in the field of biomass catalytic conversion. As a representative example, the conversion route of carbohydrates into valuable platform and commodity chemicals such as lactic acid and alkyl lactates, has already been established. The zeotype materials also possess water‐tolerant ability and are capable to be served as promising heterogeneous catalysts for aqueous reactions. Therefore, dozens of Sn‐ and Zr‐containing silica materials with various channel systems have been prepared successfully in the past decades, containing 8 membered rings (MR) small pore CHA zeolite, 10‐MR medium pore zeolites (FER, MCM‐56, MEL, MFI, MWW), 12‐MR large pore zeolites (Beta, BEC, FAU, MOR, MSE, MTW), and 14‐MR extra‐large pore UTL zeolite. This review about Sn‐ and Zr‐containing metallosilicate materials focuses on their synthesis strategy, catalytic applications for diverse reactions, and the effect of zeolite characteristics on their catalytic performances.

## Graphical abstract

This review summarizes the synthesis strategy and catalytic applications of Sn‐ and Zr‐containing metallosilicate materials, which focus on the effect of zeolite characteristics on their superior catalytic performances. The conclusions, challenges, and future directions of Sn‐ and Zr‐zeolites are also discussed.

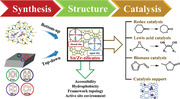

## 1 Introduction

Isomorphous incorporation of tetravalent Ti^4+^, Sn^4+^, and Zr^4+^ metal ions into silica frameworks is an effective strategy for synthesizing Lewis acid catalysts without the co‐generation of Brønsted acid sites.^[^ ^1^ ^]^ As a milestone, titanium silicalite‐1 (TS‐1) with the MFI topology was first reported in 1983, which attracted significant research interest because of the unique activated ability of the tetrahedrally coordinated Ti^4+^ ions to peroxides.^[^ ^1a^ ^]^ Subsequently, the successful insertion of tetrahedrally coordinated Sn^4+^ and Zr^4+^ heteroatoms into silica zeolite framework constructed a series of Sn‐ or Zr‐silicates. As the most studied ones, Sn/Zr‐Beta zeolites with high hydrophobicity and crystallinity have been hydrothermally synthesized in the fluoride‐mediated system.^[^ ^1b,c^ ^]^ The superiority of Sn/Zr‐Beta zeolites in water‐ tolerant characterization has aroused ongoing research in aqueous catalytic systems such as biomass conversions.

However, the traditional fluoride‐assisted route uses environmentally unfriendly fluoride ions and requires a long crystallization time (≥10 days). The obtained zeolites possess large crystal sizes at the micrometer level and upper‐limited metal loadings.^[^ ^2^ ^]^ To solve these issues, numerous researches have been documented toward ameliorating synthesis strategies and developing new metallosilicate analogs. The former mainly includes two methods, the bottom‐up (metal source existing in the initial synthesis gels) approach and the top‐down (metal atoms inserting into the already‐formed framework) approach.^[^ ^3^ ^]^ The different methods generate various targeted zeolites with significant differences in physicochemical properties, which likely results in discrepant catalytic active sites (open/closed, framework/extra‐framework, hydrated/ dehydrated species). The latter focuses on designing Sn‐ and Zr‐silicates with previously unseen topologies and different pore sizes varying form 8‐MR to 12‐MR, even mesoporous silicates.

In general, a Lewis acid is defined as an electron density acceptor. Similarly, the tetrahedral incorporation of metals (Sn/Zr) into silica hosts links with adjacent framework oxygen atoms, leading to the coordinatively unsaturated metal ions and further acting as Lewis acid sites. The framework metal ions accept electron pairs from organic reactants during the catalytic applications of Sn/Zr‐zeolites, which has no effect on the charge balance of the zeolite framework. Hence, this property is capable to adsorb and activate substrate molecules with electron‐rich groups, for example, hydroxyl and carbonyl functional groups.^[^ ^2^, ^4^ ^]^ This chemical activation is closely related to the heteroatom, reactants, and zeolite topology, which then influence the physicochemical properties of the resultant adducts including stability, flexibility, and electronic structure.^[^ ^5^ ^]^

The superiority derived from Lewis acid sites of Sn/Zr‐zeolites in activating hydroxyl and carbonyl groups has aroused considerable recent research in catalytic conversions, especially for biomass‐derived substrates. Additionally, metallosilicates can also support metal particles or clusters and then form composite catalytic materials,^[^ ^6^ ^]^ which retain the merits of Sn/Zr‐zeolites and metal species. As a result, bifunctional features or interface characteristics between metal and zeolite support are always involved in these composite materials.^[^ ^6b,c^ ^]^

Many reviews documented the synthesis of heteroatoms‐containing zeolites and their catalytic applications. For instance, in 2015, Pérez‐Ramírez et al. summarized the design of Lewis‐acid sites in zeolite for the transformation of renewables.^[^ ^3^ ^]^ Two years later, the synthesis of Sn‐containing silica materials and their catalytic performances were reviewed by Sels et al.^[^ ^2a^ ^]^ In 2022, Yu et al. systematically described the metal sites in zeolites concerning synthesis, characterization, and catalysis, which involves the partial description of preparing metal framework‐ substituted zeolite.^[^ ^6c^ ^]^ Thanks to the advances in zeolite synthesis, some emerging approaches of Sn/Zr‐zeolite synthesis appear such as structural reconstruction and interzeolite transformation. Furthermore, the scope of reactions catalyzed by Sn/Zr‐zeolites therein reported has also been broadened, in particular cooperative catalysis derived from the combination of the advantages of both metal sites and Sn/Zr‐zeolite intrinsic properties. Therefore, these current reviews cannot keep pace with the rapid evolution of this field, which motivates us to review the state‐of‐the‐art advances and discuss the present challenges and future chances.

**Figure 1:**
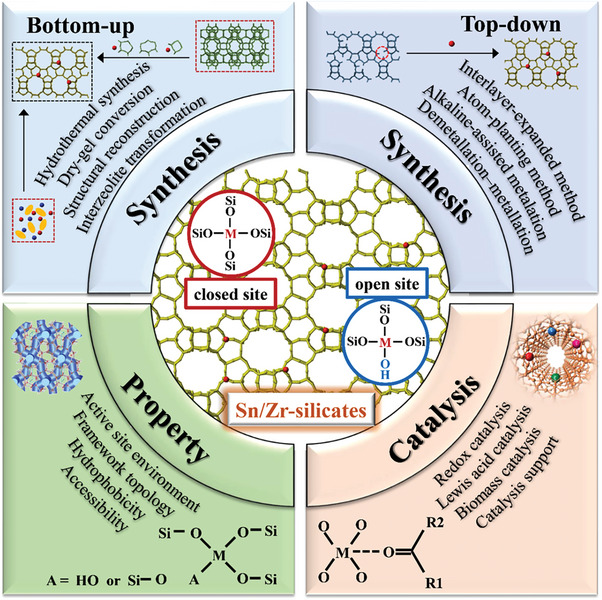
Illustration of the correlation between synthesis, property, and catalytic performance for Sn/Zr‐zeolites. The green letter “M” in open and closed sites is the abbreviation of “metal”, which represents the Sn or Zr atom.

In this context, the recent research progress in synthesizing stannosilicates and zirconosilicates is summarized (8‐MR small pore CHA zeolite, 10‐MR medium pore FER, MCM‐56, MEL, MFI, MWW zeolites, 12‐MR large pore *BEA, BEC, FAU, MOR, MSE, MTW zeolites and 14‐MR extra‐large pore UTL zeolite. Two synthetic methodologies that have been used to prepare Sn‐ and Zr‐containing silica materials will be described: (a) bottom‐up methods (e.g. hydrothermal synthesis, dry‐gel conversion, interzeolite transformation, structural reconstruction); (b) top‐down methods (e.g. atom‐planting, interlayer‐expanded, alkaline‐assisted metalation, direct incorporation of mesoporous materials, demetallation‐metalation). Subsequently, the catalytic applications of Sn‐ and Zr‐containing silica materials are also covered in the present review, which will be correlated with the physicochemical of materials (active site, framework structure, hydrophobicity, and accessibility). These catalytic applications primarily contain redox catalysis (Baeyer–Villiger oxidation and Meerwein–Pondorf–Verley–Oppenauer redox), Lewis acid catalysis (ring opening of epoxides, Aldol reaction, etc.) and biomass catalysis (sugar isomerization, synthesis of lactic acid or alkyl lactates, etc.). Moreover, Sn‐ and Zr‐zeolites are also utilized as catalysis supports of metal nanoparticles or clusters in the transformation of ethanol to butadiene, propane dehydrogenation, selective hydrogenation, etc. In summary, the synthesis‐property‐performance relationship is attempted to be constructed in order to provide essential guidance and to navigate the research direction, as shown in **Figure** **1**.

## 2 Synthesis of Sn‐ and Zr‐Zeolites

**Figure 2:**
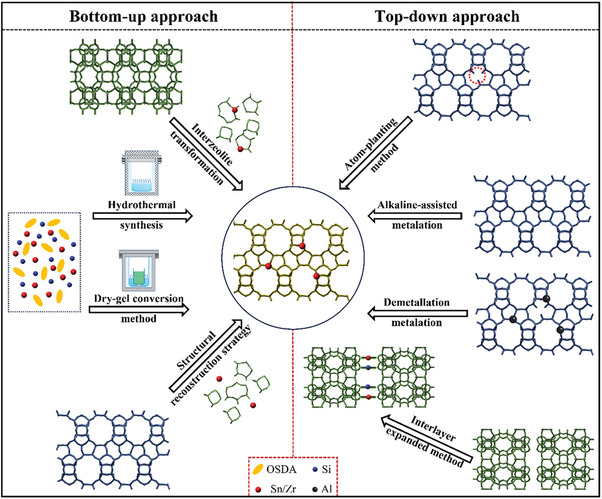
The main strategies for the synthesis of Sn/Zr‐zeolites. For simplicity, Beta topology structure is taken as an example. Black balls represent Al atoms in the initial structure for demetallation–metalation method. Dark blue balls indicate Si atoms in the interlayer‐expanded method. The other balls are colored red, which represent Sn/Zr atoms. The red dotted circle indicates silanol defects within the zeolite.

The formation of a highly ordered framework in the crystallization process is crucial to synthesize metallosilicates. In addition, the isomorphous substitution of Si with tetrahedrally coordinated heteroatoms in the framework generates catalytic active sites.^[^ ^4^ ^]^ The presence of extra‐framework metal oxide species with higher coordination states is not expected from the view of catalytic activity because they are less active and even disadvantageous for catalytic performances in some cases.^[^ ^2a^ ^]^ Therefore, the core is zeolite synthesis for catalytic applications of metallosilicates, wherein developing and designing a rational synthesis method is significantly important according to raw materials, preparation conditions, and practical demand. In general, metallosilicate zeolites are capable of being prepared by two major strategies bottom‐up and top‐down approaches. Certainly, these well‐established regulations are also suitable for the synthesis of Sn/Zr‐silicates, whose detailed synthetic strategies are summarized in **Figure** **2**.

### 2.1 Bottom‐up Approaches

#### 2.1.1 Hydrothermal Synthesis

Inspired by the great success of titanosilicate TS‐1 and other all‐silica zeolites under near neutral conditions with fluoride‐assisted crystallization, a series of metallosilicate zeolites have been explored because some metal sources are sparingly soluble or insoluble in conventional basic media.^[^ ^7^ ^]^ Whereafter, different heteroatoms (Sn, Zr, Mo, W, Hf) are introduced into numerous zeolite topology structures.^[^ ^3^, ^8^ ^]^ Herein, this review mainly focuses on Sn‐ and Zr‐containing metallosilicates with the MFI, MWW, and Beta topology structures.

##### MFI Topology Structure

MFI‐type zeolites possess a channel system with a 3D 10‐membered straight and sinusoidal ring, whose pore diameter ranges from 0.51 to 0.56 nm.^[^ ^9^ ^]^ The most common counterpart is aluminosilicate ZSM‐5 zeolite, which is extensively applied to shape‐selective reactions such as alkylation reactions and methanol to hydrocarbons as a robust solid acid catalyst.^[^ ^9a,b^ ^]^

Stannosilicate Sn‐MFI was first hydrothermally synthesized by dissolving a tin source into an aqueous mixture with the composition of sodium hydroxide (NaOH), colloidal silica, and tetrapropylammonium bromide (TPABr).^[^ ^10^ ^]^ No extra‐framework Sn oxides were observed from the characterizations of ^119^Sn MAS NMR and Sn *3d* XPS technique. This Sn framework‐substituted MFI zeolite was capable of being used as catalysis support for Pt‐based catalyst for reforming.^[^ ^11^ ^]^ Apart from the basic condition, Sn‐MFI zeolite can also be synthesized in a weakly acidic medium (pH 6.4) with the assistance of fluoride.^[^ ^12^ ^]^ In this literature, the effect of adding a sequence of raw materials on the crystallinity and catalytic activity (hydroxylation of phenol) over Sn‐MFI zeolite was examined in detail. The optimal procedure was that the Sn source (SnCl_4_ˑ5H_2_O) was first dissolved in silica source (tetraethoxysilane) followed by the introduce of tetrapropylammonium hydroxide (TPAOH). Additionally, once the Sn source was hydrolyzed in the synthesis gel, it can interact with monomeric Si(OH)_4_ (Q^0^) species, affording the generation of (OH)_3_Si‐O‐M(OH)_3_ (Q^1^), (OH)_2_Si‐[OM(OH)_3_]_2_ (Q^2^), (OH)_1_Si‐[OM(OH)_3_]_3_ (Q^3^) groups.

Subsequently, Vargas et al. found the presence of a residual tin/sodium surface phase (>3 wt.% Sn) in the resultant material through the traditional hydroxide‐ mediated strategy.^[^ ^13^ ^]^ Moreover, this formed tin/sodium overlayer eliminated the difficulty of acid or base reflux. The possible reason was the unmatched hydrolysis rate of the Sn and Si sources.^[^ ^14^ ^]^ Hence, ethylenediaminetetraacetic acid tin salt (EDTA‐Sn) and citrate were used as Sn source and buffer solution, respectively, which retard the release rate of Sn^4+^ ions to interact with Si species during the crystallization process.^[^ ^14^ ^]^ This method facilitated the tetrahedrally coordinated Sn ions insert into the framework, affording homogenous distribution in the solid product. The hydrothermal synthesis of Sn‐MFI material was also attempted in the absence of sodium species.^[^ ^15^ ^]^ The result demonstrated that Sn‐MFI prepared via the sodium‐free method displayed pure polymorphs with a uniform distribution of corresponding elements, which should be of interest to the catalytic application. Bifunctional heteroatom‐containing zeolite can also be hydrothermally synthesized from a gel comprised of Sn and Al source, where framework‐substituted Sn and Al in the zeolite framework acted as Lewis and Brønsted acid sites, respectively.^[^ ^16^ ^]^ All the obtained zeolite material had four‐coordinated Sn and Al in the zeolite framework, regardless of the Sn/Al molar ratio in the gels.^[^ ^16b^ ^]^

**Figure 3:**
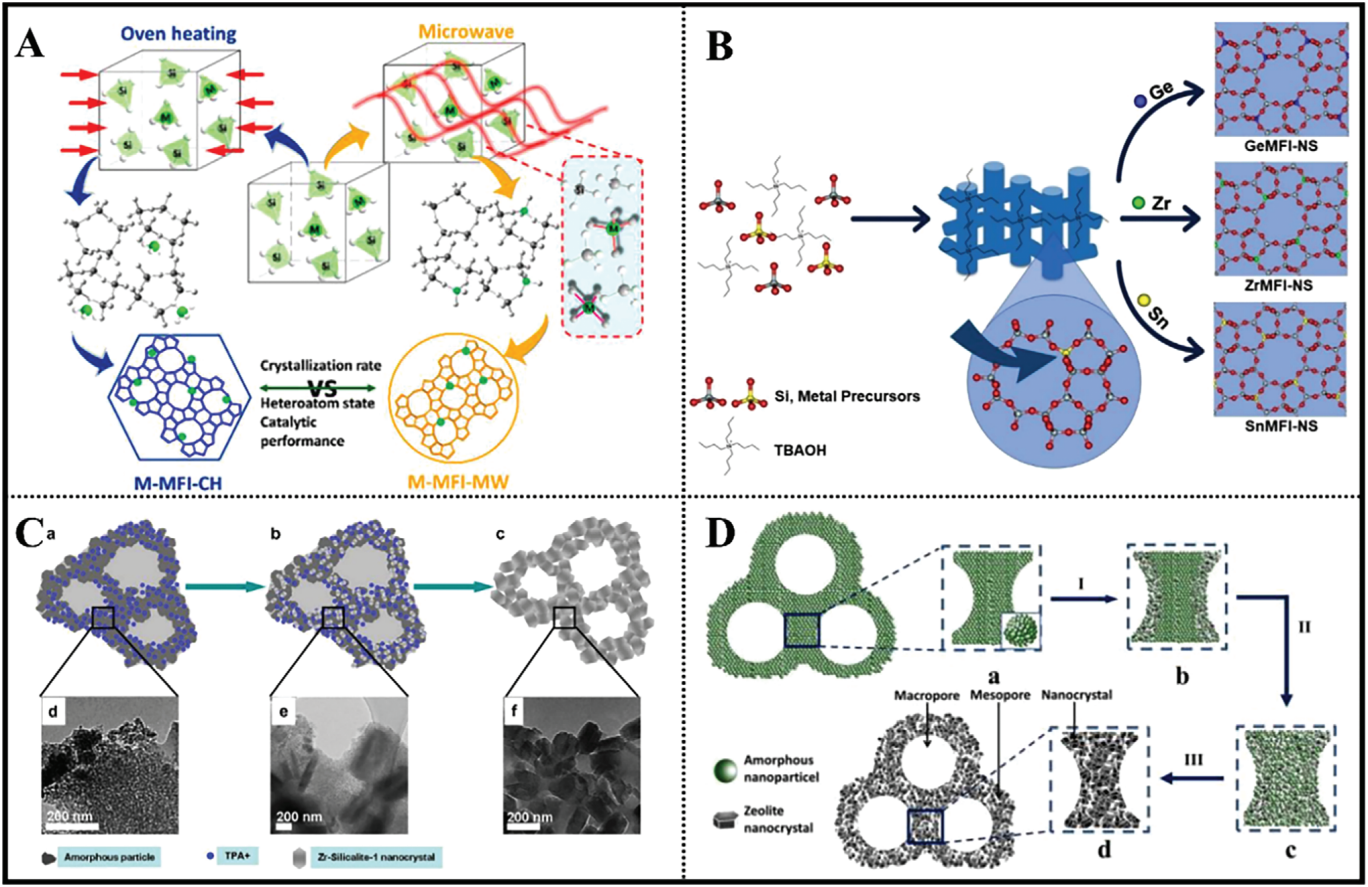
Schematic representation of the synthesis of MFI‐type Sn‐ and/or Zr‐ zeolites. A) Schematic representation of the preparation of M‐MFI heteroatom zeolite via microwave‐assisted approach. Reproduced with permission.^[^ ^18^ ^]^ Copyright 2017, American Chemical Society. B) Illustration of preparation scheme demonstrating the synthesis of various metal‐substituted MFI silicalite‐1 nanosheets. Reproduced with permission.^[^ ^20^ ^]^ Copyright 2021, Wiley‐VCH. C) The synthesis of hierarchically micro‐meso‐macroporous materials constructed from zeolite Zr‐Silicalite‐1 nanocrystals via a quasisolid‐state crystallization transformation process in a glycerin medium. Reproduced with permission.^[^ ^25^ ^]^ Copyright 2012, Elsevier. D) The crystallization transformation process to from hierarchically micro‐meso‐macroporous catalysts constructed from zeolite nanocrystals. Reproduced with permission.^[^ ^26^ ^]^ Copyright 2013, Elsevier.

With the purpose of improving the accessibility of active sites inside the pore channels and diffusion properties of reactants in reactions, hierarchical and nanosheet Sn‐MFI zeolites were born.^[^ ^17^ ^]^ Hierarchical Sn‐MFI zeolite with both micropore and mesopore was smoothly created with the assistance of microwave employing carbon as a hard template, where Sn ions mainly existed in the form of tetrahedrally coordinated state from the analysis of FT‐IR and XPS spectra.^[^ ^17a^ ^]^ Microwave‐assisted route was also used to hydrothermally synthesize MFI‐type heteroatom‐containing (Mn, Ga, Ti, Sn, Cr, and Zr) zeolites (**Figure** **3**A), where microwave field demonstrated a promoted influence on the substitution of partial heteroatoms in the zeolite framework in comparison with conventional oven heating mode.^[^ ^18^ ^]^ Nanosheet stannosilicate zeolites with MFI topology structure were successfully prepared with an amphiphilic organic structure‐directing agent (OSDA).^[^ ^17b^ ^]^ This zeolitic nanosheet was as thin as ≈2 nm in the [010] direction, giving rise to a remarkably large external surface area and mesopore volume. Ren et al. hydrothermally synthesized self‐pillared and single‐unit‐cell Sn‐MFI zeolite nanosheets with large mesopore according to repetitive branching protocol developed for the preparation of all‐silica analog.^[^ ^19^ ^]^ Additionally, as shown in Figure 3B, Sn, Ge, and Zr isomorphous substituted MFI nanosheets can be synthesized using tetra(*n*‐butyl)ammonium hydroxide (TBAOH) as the OSDA under suitable conditions.^[^ ^20^ ^]^

Zr‐substituted MFI‐topology (Zr‐MFI) zeolite was hydrothermally synthesized in the solvent of isopropanol with TPAOH as organic OSDA molecules at 180 °C for 2 d.^[^ ^21^ ^]^ The increase in unit cell volume and the presence of 963 cm^−1^ band in FT‐IR spectra indicated the incorporation of zirconium in the zeolite framework in the form of a four‐coordinated state, but the appearance of polymeric Zr oxides cannot be ruled out due to the limited characterization techniques. When the crystallized temperature was lowered down to 150 °C, the crystallized time was prolonged to 5 days,^[^ ^22^ ^]^ indicative of the positive effect of temperature under the present conditions. In situ incorporation of Zr heteroatom into MFI structure was also conducted in a fluoride medium to obtain MFI zeolites with various Al and Zr contents.^[^ ^23^ ^]^ UV–vis and TG analyses implied the isomorphous substitution of Zr in the zeolite framework. This zeolite material likely serves as a bifunctional catalyst, which is derived from Zr‐based Lewis acid sites and Al‐based Brønsted acid sites. The addition of amphiphilic [(C_2_H_5_O)_3_SiC_3_H_6_N(CH_3_)_2_C_16_H_33_]Cl in the synthetic gel of conventional hydroxide‐ mediated zirconosilicate favored the formation of nanosized Zr‐MFI zeolite.^[^ ^24^ ^]^ Zr‐MFI zeolite with tetrahedrally coordinated Zr^4+^ ions and interconnected micro‐meso‐macroporous multiple systems was also synthesized in the presence of glycerin solvent via a quasi‐solid‐state crystallization using TPAOH as OSDA (Figure 3C), where the usage of glycerin was crucial to form the hierarchical pore.^[^ ^25^ ^]^ Additionally, the pore interconnectivity and Zr location were investigated by laser‐hyperpolarized ^129^Xe NMR spectroscopy and UV–vis spectra, respectively. Afterward, they investigated the influence of synthetic parameters, for example, OSDA, scaffold templates, crystallization time, and solvent types upon the formation of Zr‐MFI with micro‐meso‐macroporous systems in Figure 3D.^[^ ^26^ ^]^ Besides, this methodology was also extended to the synthesis of targeted micro‐meso‐macroporous ZSM‐5 and Beta zeolite.^[^ ^26^ ^]^

##### MWW Topology Structure

The MWW topology structure is comprised of two independent channel systems. One channel system consists of a 12‐MR supercage with the size of 7 × 7 × 18 Å linking to each other through 10‐MR windows and the crystal surface is terminated with 12‐MR side pockets. The other pore channel system is sinusoidal 2D 10‐MR pores.^[^ ^27^ ^]^ The calcination process makes a straightforward transformation of hydrothermally synthesized 2D lamellar precursors to a 3D MWW framework structure. Additionally, the layer feature of the as‐made MWW sample facilitates its structure multiformity.^[^ ^28^ ^]^ The large external surface area derived from nanosheet crystal and side pockets on the crystal surface can not only enhance contact chance for bulky reaction substrates and active centers, but also increase diffusion performance. Thus, MWW‐type metallosilicates are highly anticipated materials in catalytic applications. The promising catalytic performance of Ti‐MWW zeolite in selective oxidation reactions attracts great interest in creating other heteroatom‐containing (Sn and Zr) MWW zeolites.

**Figure 4:**
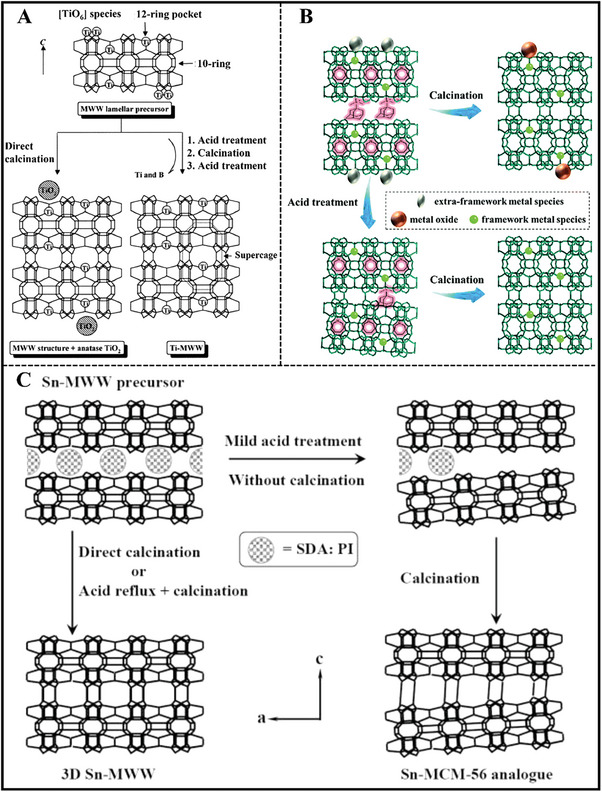
A) Schematic representation of MWW‐type titanosilicate synthesis process by combining conventional hydrothermal synthesis and acid treatment. Reproduced with permission.^[^ ^30^ ^]^ Copyright 2001, American Chemical Society. B) Structural transformation for Sn‐MWW or Zr‐MWW during post‐treatment processes. Reproduced with permission.^[^ ^33^ ^]^ Copyright 2020, Royal Society of Chemistry. C) Hydrothermal synthesis of MWW‐type stannosilicate and its post‐structural transformation to MCM‐56 analogue. Reproduced with permission.^[^ ^31^ ^]^ Copyright 2012, Elsevier.

Lv et al. first synthesized MWW topology zirconosilicate using boric acid as a crystallization‐supporting agent with the assistance of piperidine (PI) or hexamethyleneimine (HMI) OSDA through the traditional hydrothermal method, where alkali metal ions were necessary for the zeolite nucleation and further crystallization.^[^ ^29^ ^]^ Ti‐MWW zeolite can be successfully prepared under alkali‐free conditions (**Figure** **4**A),^[^ ^30^ ^]^ but only the amorphous phase was obtained even at low Zr^4+^ contents with Si/Zr molar ratio of 500 in the synthesis gels without the help of alkali metal ions.^[^ ^29^ ^]^

As for the as‐synthesized Zr‐MWW sample, acid treatment in a diluted concentration was essential for the removal of partial boron in the zeolite and extra‐framework Zr species. Zr species mainly existed in the state of isolated tetrahedral coordination in the 3D Zr‐MWW zeolite framework after suffering from the calcination process. Subsequently, Wu et al. also obtained Sn‐MWW zeolite with a uniform method (Figure 4C).^[^ ^31^ ^]^ As‐made Sn‐MWW was transformed to analogous Sn‐MCM‐56 zeolite via a structural transformation, which exhibited high external surface area as well as an opening pore system, as a result of the existence of partially delaminated MWW sheets. Meanwhile, Guo et al. reported the hydrothermal synthesis of Sn‐MWW zeolite utilizing a reversible structural transformation from the de‐boronated MWW zeolite.^[^ ^32^ ^]^ The zeolite structure went through a 3D‐2D‐3D transformation in the presence of organic HMI during this hydrothermal treatment process.

**Figure 5:**
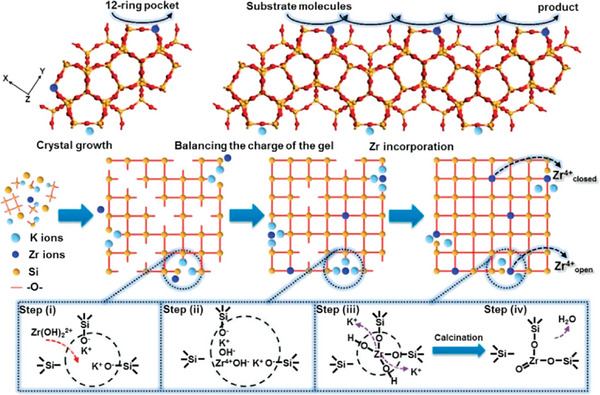
Schematic representation of tetrahedral Zr(IV) incorporated into Zr‐HSZ‐K zeolite. Reproduced with permission.^[^ ^34^ ^]^ Copyright 2021, Elsevier.

To simplify the synthesis steps and overcome the co‐existence of boron, Zhu et al. developed a dual OSDAs strategy that realized the hydrothermal synthesis of Sn‐ and Zr‐containing MWW zeolites with the organic co‐templates of HMI and *N,N,N*‐ trimethyl‐1‐adamantammoniumhydroxide (TMAdaOH) as well as the aid of K^+^ ions (Figure 4B).^[^ ^33^ ^]^ Mild acid treatment was also essential to eliminate the extra‐ framework metal species. Zou et al. successfully constructed hollow nest‐structured Zr zeolite (Zr‐HSZ) with intergrown single/double/multi‐unit‐cell nanosheet via a one‐step rotating hydrothermal method (**Figure** **5**).^[^ ^34^ ^]^ The crystallinity was rather poor in the absence of K^+^ species. Once adding it into the synthesis gels, a well‐defined MWW phase was obtained. Additionally, it was found that K^+^ ions played multiple roles during this hydrothermal process, that is, charge balance of synthesis gel, promoting the formation of Si─O─Zr bonds and expediting the crystallization process.

##### Beta Topology Structure

Beta zeolite possesses a 3D 12‐MR intersecting pore structure (0.66 × 0.67 nm), which is comprised of the intergrowth of polymorphs A and B.^[^ ^35^ ^]^ Different from the synthesis of titanosilicates, Sn atoms are difficult to isomorphous incorporation into the Si‐zeolite framework due to the relatively larger radius (0.71 Å) and longer Sn─O bond length (1.912 Å) of Sn^4+^ by comparing with these (0.68 and 1.892 Å, respectively) of Ti^4+^. In addition, the hydrolysis rate of the Si and Sn source was incompatible.^[^ ^36^ ^]^

**Figure 6:**
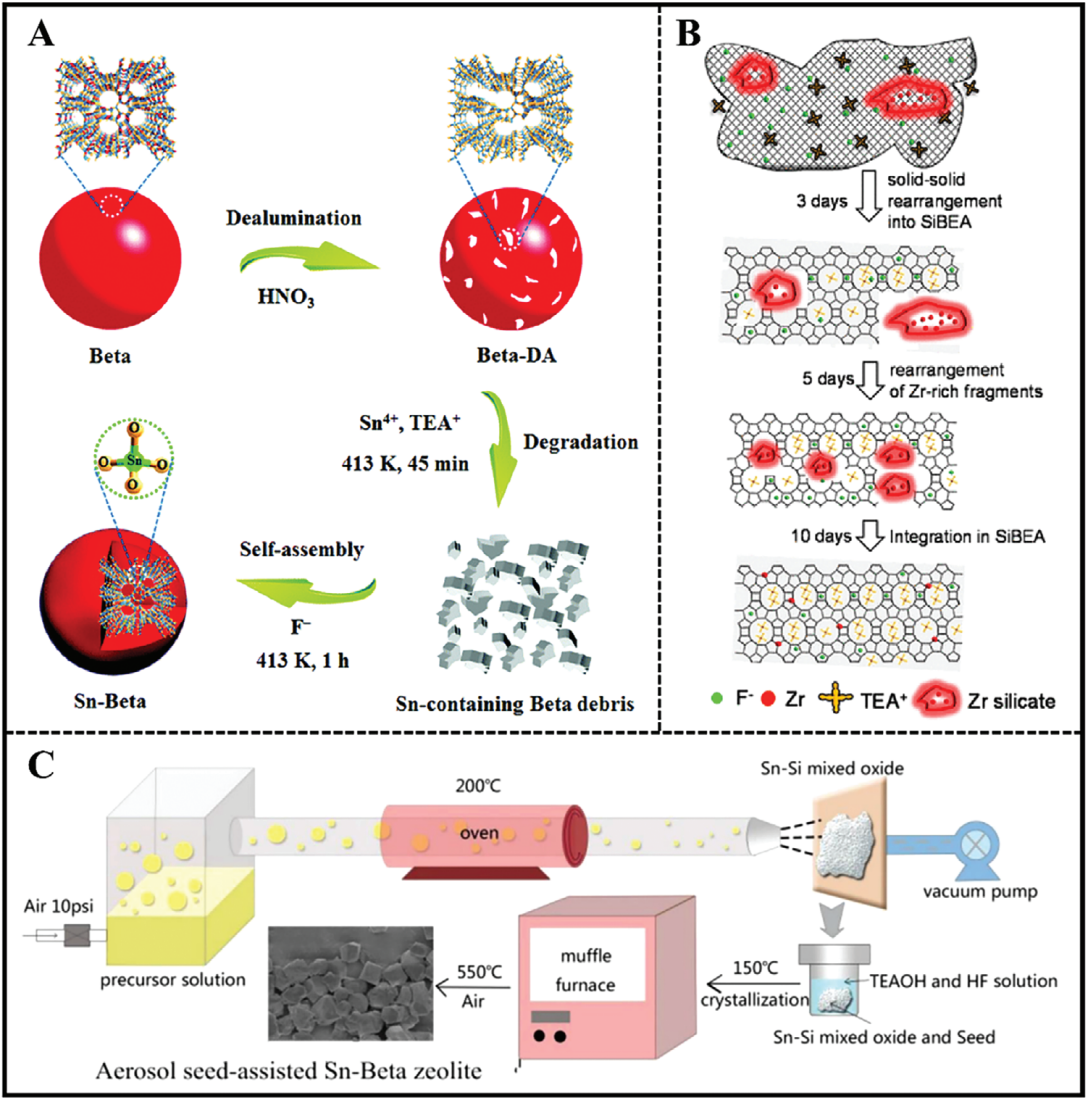
A) Schematic illustration for the synthesis of Sn‐Beta zeolite by structural reconstruction. Reproduced with permission.^[^ ^38^ ^]^ Copyright 2017, Royal Society of Chemistry. B) Proposed mechanism of Zr‐BEA crystallization. Reproduced with permission.^[^ ^57^ ^]^ Copyright 2018, American Chemical Society. C) The aerosol‐seed‐ assisted hydrothermal synthesis of Sn‐Beta zeolite. Reproduced with permission.^[^ ^47^ ^]^ Copyright 2018, Elsevier.

In 1997, Mal et al. first synthesized the Sn‐Beta stannosilicate by hydrothermal method.^[^ ^37^ ^]^ The synthesis gel composed of 33.3 SiO_2_: 0.0–0.42 SnO_2_: 0.0–1.0 Al_2_O_3_: 17.3–23.3 tetraethylammonium hydroxide (TEAOH): 660 H_2_O contributed to the formation of Sn‐Al‐Beta zeolite at 142 °C for 10 days. Al‐free Sn‐Beta was capable of obtaining by adding a certain amount of highly dealuminated Sn‐Al‐Beta as seeds. However, the coordinated state of Sn^4+^ ions was not certain depending on the limited analysis technique in the literature. What's more, the obtained Sn‐Beta zeolite encountered high hydrophilicity resulting from abundant defects in the framework, which was a distinct disadvantage for the adsorption of substrates upon the framework active centers to some extent (**Figure** **6**A).^[^ ^38^ ^]^ Shortly afterward, Corma et al. first disclosed a patent that was related to the synthesis of Sn‐Beta zeolite, which became the most universal strategy in the following period.^[^ ^39^ ^]^ On the one hand, the incorporated fluoride ions (mineralizing agents) contributed to the nucleation process. On the other hand, the fluoride‐assisted route led to low supersaturation, therein requiring long crystallization time (>40 d) and forming large crystal size (>1 µm) with upper‐limited Sn content (generally Si/Sn molar ratio ≥100) in the resulant framework.^[^ ^37^, ^40^ ^]^ Hence, research about the synthesis and applications is rarely reported in the following decade.

Until 2010, Davis et al. reported that Sn‐Beta zeolite exhibited excellent catalytic ability in the isomerization of glucose to fructose,^[^ ^41^ ^]^ which motivated researchers to pay close attention to Sn‐Beta again. The crystallization time of Sn‐Beta can be decreased to 11–12 days when using all‐silica Beta as seeds.^[^ ^42^ ^]^ Fan et al. investigated the influence of seeds (morphology and dispersed state) on the crystallization kinetics of Sn‐Beta zeolite synthesis.^[^ ^43^ ^]^ The addition of dealuminated zeolite seeds with a crystal size of 200 nm reduced the synthesis time from above 20 to 2 days.

Moreover, the synthesis parameters in the synthesis gels also affected the complete crystallization time of Sn‐Beta, such as Sn and H_2_O amounts.^[^ ^44^ ^]^ Tolborg et al. used an un‐seeding method to investigate the synthesis time of Sn‐Beta with discrepant Sn contents in the initial gels.^[^ ^44b^ ^]^ It was found that the complete crystallization time was remarkably different with the variation of Sn contents, for example, low Sn contents (Si/Sn = 400), 4 days; moderate Sn content (Si/Sn = 200‐150), 7–14 days; high Sn content (Si/Sn = 100), up to 60 days. With respect to H_2_O contents, decreasing the H_2_O/SiO_2_ molar ratio can reduce the crystallization time of Sn‐Beta under the same Si/Sn molar ratio to some extent,^[^ ^44c^ ^]^ for instance, H_2_O/SiO_2_ = 7.5, 10 days; H_2_O/SiO_2_ = 6.8, 7 days; H_2_O/SiO_2_ = 5.6, 4 days. In spite of accelerating the crystallization of Sn‐Beta zeolite when the water content was reduced in the synthesis gel, it was unfavorable for the insertion of Sn^4+^ ions into the zeolite framework, giving rise to the formation of more undesired extra‐framework Sn oxide species.^[^ ^45^ ^]^

Sun et al. utilized stainless steel reaction tubes, other than traditional polytetrafluoroethylene lined autoclave, to load the final synthesis gels for the hydrothermal synthesis of Sn‐Beta with the addition of Beta seeds.^[^ ^46^ ^]^ Only 36 h was needed to achieve the full crystallization with the Si/Sn molar ratio of 200, which was ascribed to the better heat transfer of the stainless‐steel reactor. Meng et al. used an aerosol‐seed‐assisted method to synthesize Sn‐Beta zeolite in order to lower the template and F^−^ ions contents, and shorten the crystallization time (Figure 6C).^[^ ^47^ ^]^ The addition of Beta seeds can not only facilitate the crystallization but also was advantageous for the decrease of Sn‐Beta crystal size. The optimal crystallization condition was 1 SiO_2_: 0.4 TEAOH : (0.006–0.01) SnO_2_: 0.4 HF: 5.6 H_2_O with 3 wt.% of seeds at 150 °C for 5–9 days. Subsequently, an aerosol‐assisted hydrothermal method coupled with further mild base treatment was carried out to increase the Sn content and the diffusion property.^[^ ^48^ ^]^ Sn‐Si aerosol powder was crystallized in a quasi‐solid state (H_2_O/SiO_2_ = 2.8), which gave the formation of plate‐like Sn‐Beta zeolite with a Si/Sn molar ratio of 76 within 7 days. Next to this step, a mild base treatment was performed to obtain the hierarchical Sn‐Beta zeolite.^[^ ^48^ ^]^ In 2020, the same group employed aerosol‐based hollow Sn‐Si mixed oxide as the precursor to prepare Sn‐Beta zeolite with high Sn content and mesoporous channels.^[^ ^49^ ^]^ Note that the H_2_O/SiO_2_ molar ratio in the initial synthesis gel can be further decreased to 1.0. Nonetheless, as can be seen from the UV–vis spectra, a majority of extra‐framework Sn species existed in these aerosol‐assisted Sn‐Beta zeolites.^[^ ^47^, ^48^, ^49^ ^]^ Analogously, Takayuki et al. also synthesized Sn‐Beta zeolite with the Si/Sn molar ratio of 60 using a Sn‐Si mixed oxide precursor.^[^ ^50^ ^]^

The positive charge of OSDA in the as‐made all‐silica or metallosilicates via hydrothermal synthesis is usually balanced by the framework defect sites. It has been demonstrated that fluoride not only facilitates the mineralization of silica source but also matches partial positive charge in OSDA molecule.^[^ ^36^ ^]^ Hence, fluoride‐mediated Sn‐Beta zeolite exhibits fewer framework defect sites and high hydrophobicity, which is favorable for the catalytic applications of Sn‐Beta catalyst in aqueous system.^[^ ^38^ ^]^ Considering environmental pollution from fluoride, non‐fluoride synthesis of highly crystalline Sn‐Beta zeolite was realized using *N*‐cyclohexyl‐*N,N*‐ dimethylcyclohexanaminium hydroxide and Beta as OSDA and seeds, respectively, with the aid of Na^+^ ions.^[^ ^51^ ^]^ The added amounts of Na^+^ ions matched with Sn contents in the synthesis gels. If the as‐synthesized Sn‐Beta zeolite was directly calcined at 550 °C, its framework structure collapsed severely. When the Na^+^ ions were exchanged by NH_4_^+^ ions, the highly crystalline Sn‐Beta zeolite was endowed after a further calcination process.^[^ ^51^ ^]^

Zirconosilicate zeolite is difficult to hydrothermally synthesize due to the fact that Zr^4+^ ions possess a larger ionic radius (0.72 Å) than Sn^4+^ ions (0.71 Å),^[^ ^52^ ^]^ resulting in Zr^4+^ ions are untoward to be isomorphously incorporated into the zeolite framework under traditional basic condition. Therefore, the hydrothermal synthesis of Zr‐Beta zeolite requires the assistance of Al^3+^ ions.^[^ ^53^ ^]^ Inspired by the great success of Sn‐Beta zeolite, Jaenicke et al. first reported the hydrothermal synthesis of Al‐free Zr‐Beta zeolite with the fluoride‐assisted strategy using dealuminated Beta zeolite as seeds in a fluoride medium.^[^ ^53^ ^]^ Along with increasing Zr content in the synthesis gel, a longer crystallization time was needed. When the Si/Zr molar ratio was higher than 75, fully crystallized product cannot be obtained even extending the time to 30 days. Whereas, the XRD patterns of Zr‐Beta zeolite with different Zr contents were not presented in the literature. This fluoride‐mediated method opens up the avenue for the hydrothermal synthesis of Zr‐Beta zeolite. Subsequently, the authors exhibited these XRD profiles in 2004.^[^ ^54^ ^]^ Additionally, it was found that the substitution of Zr^4+^ ions into pure‐silica Beta zeolite contributed to the enhancement in the relative ratio of polymorph B to A. In 2006, the same group used the same fluoride‐assisted strategy to hydrothermal synthesize Zr‐Beta and Zr‐Al‐Beta zeolite.^[^ ^55^ ^]^ 27Al MAS NMR spectra revealed that Al species was located in the zeolite framework, affording the formation of the Brønsted acid site detected in pyridine‐adsorbed FT‐IR spectra. However, this acid site was detrimental to the product selectivity in the Meerwein‐Ponndorf‐Verley (MPV) reduction of cinnamaldehyde. Actually, there were dual active sites containing Brønsted and Lewis acid sites in Zr‐Al‐Beta zeolite, which did not arouse sufficient attention at that time.^[^ ^55^ ^]^

Soon afterward, a fluoride‐assisted approach is employed to hydrothermally synthesize Zr‐Beta zeolite by many groups.^[^ ^22^, ^56^ ^]^ For instance, Bui et al. also synthesized Zr‐Beta zeolite from the synthesis gel with a molar composition of SiO_2_: 0.01 ZrOCl_2_: 0.55 TEAOH: 0.54 HF: 7.52 H_2_O at 140 °C for 40 days.^[^ ^56a^ ^]^ It should be pointed out that the fluoride source was commonly extremely dangerous HF rather than relatively less dangerous NH_4_F for the fluorine‐assisted approach, which was discrepant for the hydrothermal synthesis of Sn‐Beta zeolite.

The synthesis mechanism for preparing Zr‐Beta zeolite was investigated by Kots group in Figure 6B.^[^ ^57^ ^]^ The first step in the course of hydrothermal synthesis was the formation of all‐silica Beta according to a solid‐solid rearrangement route. Zr species was then inserted into a crystal with a hexa‐coordinated state, located in a square‐bipyramidal position of zirconium silicate fragments. At this moment, the CO‐adsorbed FT‐IR spectra proved that the Zr species failed to generate Lewis acidity. For the second step, Zr‐rich fragments interacted with initial Si‐Beta through a solid‐solid rearrangement mechanism, producing isolated Zr (IV) in tetrahedral coordination. Besides, CO‐adsorbed FT‐IR spectra confirmed the framework substituted Zr^4+^ ions mainly existed in the state of closed Lewis Zr^4+^ sites. The closed Zr^4+^ sites were transformed into open sites prolonging the crystallization time.^[^ ^57^ ^]^ Hence, precisely controlling the crystallization time was essential for a specific catalytic application of Zr‐Beta zeolite, which was determined by the competition of intrinsic catalytic active sites (i.e., open and closed Zr^4+^ sites). This developed rule was further confirmed by the same group.^[^ ^56d^ ^]^ The synthesis gel for the preparation of Zr‐Beta zeolite with the Si/Zr molar ratio of 200 was subjected to hydrothermal treatment with different crystallization times (in the case, 10 and 15 days). A series of characterizations showed that these two products possessed the same structure, morphology, crystal size, Zr content, and texture properties. But Zr‐Beta synthesized for 10 days had only closed sites. Regarding Zr‐Beta zeolite with a longer synthesized time of 15 days, both open and closed sites were exhibited.^[^ ^56d^ ^]^

To date, Zr‐Beta zeolite has received little attention in comparison with Sn‐Beta, which generally served as a reference material to highlight the superiority of Sn‐Beta catalysts in catalytic applications. Consequently, the hydrothermal synthesis strategy and corresponding application study of Zr‐Beta is much less than those of Sn‐Beta zeolite.

##### Other Topology Structures

Since the zeolite hosts (MFI, MWW, Beta topology) of Sn (IV) or Zr (IV) have attracted remarkable attention for the transformation of renewables, the incorporation of Sn^4+^/Zr^4+^ ions into other topology structures has also been tried.

Harris et al. proposed the direct hydrothermal synthesis of chabazite zeolite (3D 8‐MR) with tetrahedrally coordinated Sn ions in the framework (Sn‐CHA).^[^ ^58^ ^]^ In general, this synthesis route involved two key steps, containing the uniform mixture of silica source of tetraethylorthosilicate and the Sn source of stannic chloride pentahydrate in an aqueous H_2_O_2_ solution and then introducing the organic structure‐directing agent of TMAdaOH. After a period of 24 h, ethanol, and excess water evaporated from this mixture to make H_2_O/SiO_2_ = 3. It should be mentioned that one intermediate rehydration step was required to keep H_2_O/SiO_2_ = 40 and then the solution was evaporated to H_2_O/SiO_2_ = 3 again. Ultimately, aqueous HF was added as the mineralizing agent, which was crystallized at 150 °C for 2 days. Note that Sn‐CHA cannot be crystallized successfully without this rehydration cycle. DNP NMR, UV–vis, and X‐ray absorption spectra showed that Sn ions were inserted into the zeolite framework. For dehydrated Sn‐CHA, there were framework Sn sites with (defect) and without (closed) neighboring Si defect sites. The exposure to the ambient atmosphere led to the formation of hydrated defects and hydrolyzed open sites.^[^ ^58^ ^]^

Al‐free Sn‐ZSM‐11 (MEL topology, Si/Sn > 40) zeolite consists of 3D 10‐MR channels, which were prepared according to a similar protocol for the hydrothermal synthesis of all‐silica ZSM‐11.^[^ ^59^ ^] 119^Sn MAS NMR and XRD implied that most of Sn species were in octahedral coordination, that is, a small part of Sn^4+^ ions existed in the zeolite framework. In spite of the uncertainty of the Sn^4+^ micro‐environment, these stannosilicates were quite active in the hydroxylation of phenol and toluene with the oxidant of aqueous H_2_O_2_.^[^ ^59^ ^]^ Subsequently, Sn‐MEL was hydrothermally synthesized depending on similar procedures with an organic template of tetrabutylammonium hydroxide (TBAOH). The textural properties and morphology of the stannosilicate counterpart were comparable to those of aluminosilicate ZSM‐11 zeolite.^[^ ^60^ ^]^

The tetrahedrally coordinated Sn ions can also be inserted into all‐silica ZSM‐12 (MTW topology) framework with 1D 12‐MR channels by hydrothermal method using hexamethylene bis(benzyldimethyl ammonium hydroxide) as OSDA.^[^ ^61^ ^]^ Unit cell expansion derived from XRD profiles, FT‐IR, and UV–vis spectra suggested the successful substitution of Sn^4+^ into the MTW framework.

Zr‐Al‐Y zeolite was prepared using ZrSO_4_ as a Zr source with OSDA‐free and Al‐assisted hydrothermal route at 98 °C for 18 h, forming Al‐rich materials with SiO_2_/Al_2_O_3_ molar ratio of 5.3.^[^ ^62^ ^]^ The presence of isolated framework Zr^4+^ ions in Y zeolite benefited the formation of intra‐crystalline mesoporous during the subsequent steam treatment process.^[^ ^62^ ^]^

#### 2.1.2 Dry‐gel Conversion Methods

**Figure 7:**
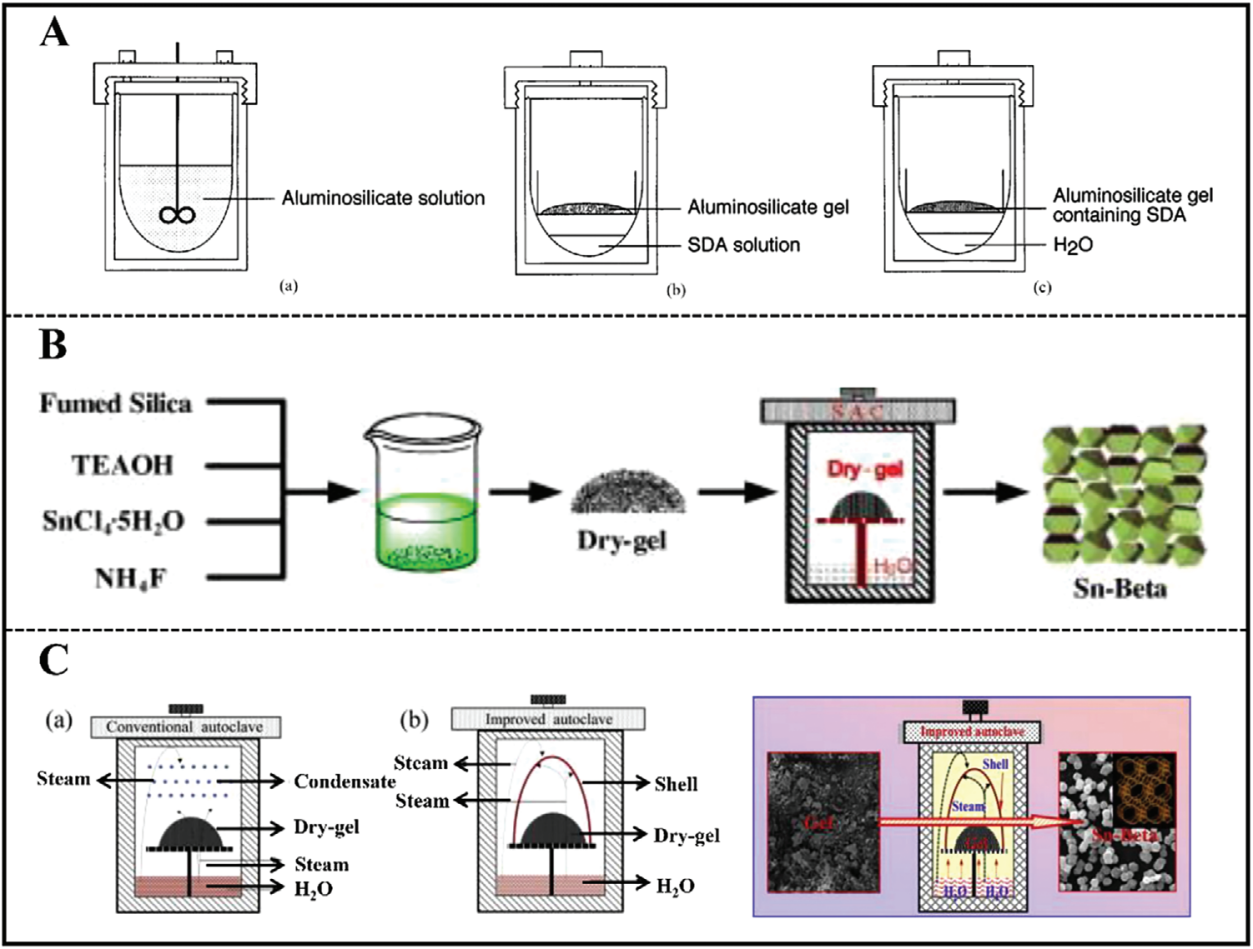
A) Schematic diagram of the methods for microporous crystal synthesis. (a) Hydrothermal method, (b) vapor‐phase transport (VPT) method, and (c) steam‐assisted crystallization (SAC) method. The latter two are the dry gel conversion (DGC) methods. Reproduced with permission.^[^ ^65^ ^]^ Copyright 1999, Springer US. B) Illustration of the Sn‐Beta zeolite preparation by SAC method. Reproduced with permission.^[^ ^69^ ^]^ Copyright 2012, Elsevier. C) Water evaporation and condensation processes in the conventional autoclave (a) and improved autoclave (b) for the synthesis of Sn‐Beta zeolites using the SAC method. Reproduced with permission.^[^ ^70^ ^]^ Copyright 2013, Elsevier.

Dry gel conversion (DGC) is a useful method for the synthesis of zeolite, which can be employed for the preparation of high‐silica or even all‐silica zeolite.^[^ ^63^ ^]^ In 1990, Xu et al. first reported the successful transformation of dried amorphous aluminosilicate gels to crystalline ZSM‐5 zeolite under the vapor atmosphere.^[^ ^64^ ^]^ The dried gel was put in the middle of the autoclave in the altitude direction and the mixed solution of trimethylamine and ethylenediamine was placed in the bottom, avoiding the direct contact of solid and liquid phase. This reaction system was heated statically at 180–200 °C for 5–7 days, forming ZSM‐5 zeolites. The silanol groups in dried gels became active and further reacted in the vapor atmosphere from the liquid phase at the bottom. DGC is an alternative method for these metallosilicate zeolites that cannot be prepared with the conventional hydrothermal route. This appealing method exhibits several characteristics as follows, I) high zeolite yield and easily controlling corresponding element contents in targeted zeolite; II) circumventing the filtration step and rarely producing liquid waste; III) high crystallinity and purity; IV) reducing the OSDA contents to some extents; V) a short crystallized time. Two detailed methods are divided, that is, vapor‐phase transport (VPT) if the OSDAs are volatile that are not contained in the dried gel; and steam‐assisted crystallization (SAC) for non‐volatile OSDAs that are added into the gel (**Figure** **7**A).^[^ ^65^ ^]^

At present, DGC is increasingly used in the synthesis of metallosilicate zeolites with different heteroatoms and topology structures.^[^ ^66^ ^]^ It is also the case for Sn‐ and Zr‐zeolites. Niphadkar et al. systematically studied the influence of synthesis parameters (TPA^+^ amounts, Si/Sn molar ratio, water amounts in the autoclave bottom, crystallization temperature, and crystallization time) upon the synthesis of Sn‐MFI zeolite by the SAC route.^[^ ^67^ ^]^ Compared with the traditional hydrothermal method, the SAC strategy required less TPA^+^ amounts, shorter crystallization time, and also reduced water waste under comparable Sn contents conditions.^[^ ^67^ ^]^ UV–vis spectra demonstrated Sn^4+^ ions centered in the MFI zeolite framework. Sn‐MFI zeolite was also obtained from the transformation of SiO_2_‐SnO_2_ xerogel in a quasi‐solid phase.^[^ ^68^ ^]^

Kang et al. synthesized Sn‐Beta zeolite in a fluoride medium using the SAC method in 2013 (Figure 7B),^[^ ^69^ ^]^ which needed a shorter crystallization time than the conventional hydrothermal strategy. For example, only 5 h was needed at 180 °C when the Si/Sn molar was 125 in the dried gels. Interestingly, the Si/Sn molar ratio can reach low to 83 in the well‐crystallized product with the synthetic time of 60 h, which broke through the restriction of Sn contents (Si/Sn ≥ 100) for the hydrothermal method. Subsequently, this same group investigated the effect of gel drying temperature, water amount in the bottom, crystallization temperature, and crystallization time on the synthesis of Sn‐Beta zeolite (Figure 7C).^[^ ^70^ ^]^ Note that the interior construction of the SAC autoclave also impacted the crystallization process. In general, the condensed water dropped back into the dried gel and changed the gels composition in the conventional autoclave. To solve this issue, an oval glass was constructed that covered the gel, which guided the condensed water from the steam back to the bottom. This modified SAC autoclave positively contributed to the formation of Sn‐Beta, which was not sensitive to the water contents in the bottom of the autoclave. The optimized synthesis conditions were listed as follows, gel molar composition of 100 SiO_2_: 0.8 SnO_2_: 27 (TEA)_2_O: 54 NH_4_F: 750 H_2_O, synthesis gel dried at 60–100 °C for 6–24 h, crystallization in a saturation steam at 160–200 °C for 6–12 h.^[^ ^70^ ^]^

To avoid the environmental pollution of fluoride, Chang et al. synthesized Sn‐Beta zeolite with dealuminated Beta seeds in a non‐fluoride medium according to the SAC route.^[^ ^36^ ^]^ The addition of Beta seeds not only directed the formation of the Beta zeolite framework but also accelerated the growth of zeolite crystals. What's more, the framework structure collapsed if the as‐synthesized Sn‐Beta zeolite was directly calcined. Fascinatingly, it was found that the zeolite framework can be well‐preserved for NH_4_^+^‐exchanged Sn‐Beta zeolite prior to calcination, which was similar to the reported fluoride‐free synthesis of Sn‐Beta.^[^ ^51^ ^]^ This fluoride‐free synthesized Sn‐Beta zeolite in a hydroxide medium exhibited more hydrophilic than the fluoride‐assisted method because more framework Si‐O^−^ groups were needed to compensate for the residual TEA^+^ in the crystallization systems, forming abundant silanol groups after calcination at elevated temperature.^[^ ^51^ ^]^

#### 2.1.3 Interzeolite Transformation

Interzeolite transformation utilizes a parent zeolitic source to synthesize another zeolite framework. Initially, synthesis pioneer Richard M. Barrer synthesized the KFI zeolite without its natural counterpart using analcime zeolites (ANA) as the parent source under geothermal conditions.^[^ ^71^ ^]^ The conversion process is profoundly affected by the properties of the parent zeolite and synthetic parameters (e.g. crystallization temperature and time).^[^ ^72^ ^]^ Okubo et al. considered that parent and daughter zeolites possessed at least one common composite building unit (CBU), named the CBU hypothesis, to overcome kinetic and thermodynamic barriers.^[^ ^73^ ^]^ This would affect the evolution processes of nucleation and crystallization. Many aluminosilicate zeolites have been synthesized via this interzeolite transformation method such as RUT. MFI, MTN, CHA, *BEA, and LEV topology structure zeolites.^[^ ^72^, ^74^ ^]^

As for heteroatom‐containing zeolites, fluoride is necessary to gain certain zeotypes with high hydrophobicity for the hydrothermal synthesis method.^[^ ^75^ ^]^ In alkaline media, some zeotypes go through inferior crystallization behavior compared to their aluminosilicate version.^[^ ^1^, ^37^ ^]^ Consequently, it is a chance to apply an interzeolite transformation strategy to prepare certain metallosilicate zeolites such as stannosilicates and zirconosilicates, resulting from the ease of this conversion.

The interzeolite transformation process from heteroatoms‐containing FAU‐type zeolite (M‐Al‐FAU) to heteroatom‐incorporated CHA‐type zeolites (M‐Al‐CHA, M = Sn, Fe, Ga) was realized successfully. It failed to synthesize M‐Al‐CHA when the amorphous hydrogel was used as raw materials under similar hydrothermal conditions.^[^ ^76^ ^]^ The pristine M‐Al‐FAU was post‐synthesized from dealuminated FAU zeolite under acidic conditions, which was then used as parent zeolite to produce CHA zeolite with the aid of organic TMAdaOH and CHA seeds at 170 ^o^C for 2 days. Electrospray ionization mass spectrometry (ESI‐MS) indicated the transformation of FAU to CHA occurred in the solid phase. Metal species from the decomposition of M‐Al‐FAU zeolite were again substituted into the CHA zeolite framework with the maintaining of a coordination state.^[^ ^76^ ^]^

**Figure 8:**
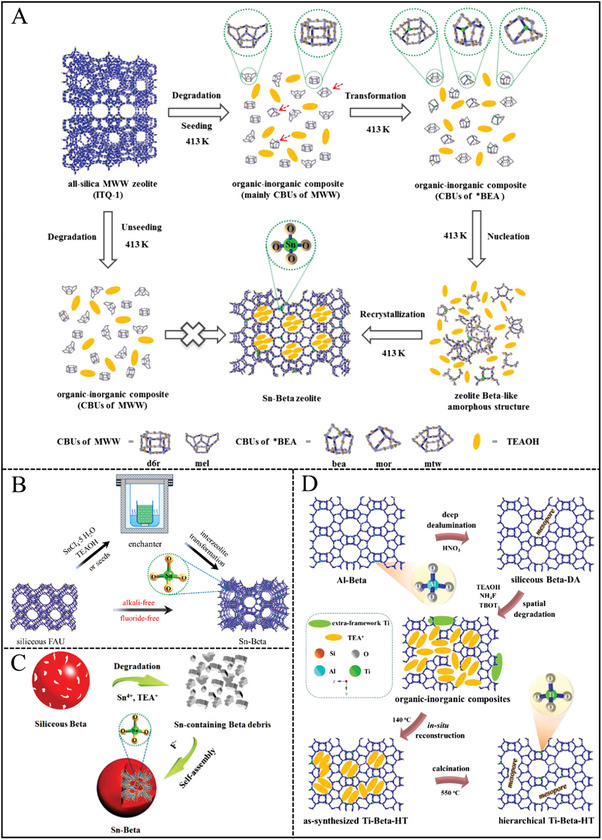
A) Proposed crystallization mechanism for the interzeolite transformation of pristine ITQ‐1 to Sn‐Beta zeolite. Reproduced with permission.^[^ ^77^ ^]^ Copyright 2017, Elsevier. B) The crystallization process for nanosized Sn‐Beta zeolite synthesized from siliceous FAU zeolite without the participation of fluoride and alkali metal ions. Reproduced with permission.^[^ ^79a^ ^]^ Copyright 2019, Elsevier. C) Schematic illustration for the synthesis of Sn‐Beta zeolite by structural reconstruction. Reproduced with permission.^[^ ^38^ ^]^ Copyright 2017, Royal Society of Chemistry. D) The crystallization mechanism for the preparation of hierarchical Ti‐Beta zeolite via in situ structural reconstruction strategy. Reproduced with permission.^[^ ^91^ ^]^ Copyright 2021, Royal Society of Chemistry.

The traditional hydrothermal synthesis of Sn‐Beta zeolite needed long crystallization time and the resultant material exhibited the upper‐limited Sn contents and large crystal size. Therefore, significant research has been done to improve the synthesis method. Interzeolite transformation is an effective strategy among these methodologies. Zhu et al. synthesized Sn‐Beta zeolite using pure silica MWW (ITQ‐1) as parent zeolite within a short time of 3 days. The final Sn‐Beta sample possessed a high isolated Tin loading of 3.03 wt.% (Si/Sn = 63).^[^ ^77^ ^]^ This strategy was also extended to the synthesis of all‐silica Beta and Ti‐Beta.^[^ ^78^ ^]^ As shown in **Figure** **8**A, the revolution process followed the dissolution‐recrystallization mechanism. In addition, the added Beta seeds as well as the structural similarity existing between the parent MWW and daughter Beta zeolites were two key factors during the crystallization process. Compared with the traditional fluoride‐assisted strategy, this method was helpful in forming small‐sized Sn‐Beta zeolite with high hydrophobicity.^[^ ^78^ ^]^ Subsequently, this same group designed an interzeolite transformation route for the synthesis of Sn‐Beta from siliceous Y zeolite with steam‐assisted conversion in the absence of fluoride and alkali metals (Figure 8B).^[^ ^79a^ ^]^ The crystal size of the obtained Sn‐Beta was in the range of 50–150 nm. Moreover, the Sn content in the Beta framework was ≈2.39 wt.% (Si/Sn molar ratio of 81), which exceeded that of the classic fluoride‐mediated method. Unfortunately, this material encountered inferior hydrophobicity, which was similar to Sn‐Beta synthesized under an alkaline medium.^[^ ^36^, ^51^ ^]^ Herein, the added Beta seed was only a crystallization accelerator, which was different from the above‐mentioned interzeolite transformation of ITQ‐1 zeolite.^[^ ^77^ ^]^ The crystallization time was prolonged from 3 to 13 days in the absence of Beta seeds with comparable Sn contents (Si/Sn = 120).^[^ ^79a^ ^]^ In 2023, they synthesized hydrothermally a nanosized Sn‐beta zeolite with a crystal size of 20–50 nm from a low‐cost siliceous FAU zeolite via interzeolite transformation method, which was the minimum one among the reported hydrothermal synthesized Sn‐beta zeolite. In addition, the crystallization process was tracked to reveal that Sn ions were incorporated into the silicate debris in the tetrahedrally coordinated state before establishing the zeolite framework.^[^ ^79b^ ^]^

The interzeolite transformation generally starts from the high‐silica or all‐silica zeolite. On the one hand, the residual Al species in the parent zeolite can be converted into targeted zeolites, acting as Brønsted acid sites, which is likely harmful to their catalytic performances.^[^ ^80^ ^]^ On the other hand, the siliceous zeolites suffer from fast dissolution and high silica solubility in an alkaline synthesis medium.^[^ ^81^ ^]^

Compared with self‐condensation, heteroatoms tend to condense with silicates in an alkaline medium.^[^ ^82^ ^]^ Owning to this condensation tendency on silicate surfaces, the heteroatom concentration in the liquid is extremely low, which results in difficulty in forming (hydr)oxide species despite very high heteroatom contents in the synthesis gels.^[^ ^81b^ ^]^ Whereas, metal ions in the synthesis gels are reported to suppress zeolite nucleation and crystal growth, especially for metal ions with high contents.^[^ ^77^, ^79^ ^]^ Moreover, high metal ions contents likely affect the aggregation of large particles and guard its surroundings from dissolution because the Si─O─Si bond is preferentially dissolved, naturally, decreasing the subsequent dissolution of silicates and further lowering saturation. The tendency of metal─O─Si bond formation is deemed as the function of reversible condensation‐polymerization reaction, which may be influenced by bond‐length, charge density, nucleophilicity, and so on.^[^ ^81b^ ^]^

Interzeoltie transformation strategy possesses great advantages in the synthesis of metallosilicates compared to the traditional hydrothermal method, such as shorter crystallization time, high framework metal contents, and nanosized crystals. Additionally, a higher supersaturation in the reaction gel facilitated the formation of targeted zeolite with well‐crystallinity. In this regard, interzeolite transformation is significant for enriching the metallosilicate zeolites community and satisfying the purpose of processing the catalytic applications.

#### 2.1.4 Structural Reconstruction Strategy

The hydrothermal process of zeolite crystallization involves four steps: 1) depolymerization of a silica source, 2) repolymerization of polysilicate, 3) nucleation as well as nucleus growth, and 4) crystal growth.^[^ ^83^ ^]^ Silica source, the most important initial raw material, affected the synthesis process due to distinctive textural properties, particle size, and dissolution rate. The type of silica source was proved to be correlated to the polymerization state and supersaturation of silicate ions.^[^ ^84^ ^]^ Amorphous silica sources are demonstrated to construct CBUs under the induction effect of the organic template and subsequently establish the corresponding zeolite framework according to the self‐assembling mechanism, which is the core and rate‐determining step during the whole crystallization process for classic spontaneous nucleation theory.^[^ ^81^, ^85^ ^]^ Note that two phenomena usually exist for the traditional synthesis of metallosilicate zeolites, (I) difficulty in nucleation resulting in the frustrated crystallization, (II) upper‐limited metal contents and long crystallization time caused by the inhibiting effect of metal‐substituted ions to nucleation and zeolite growth.^[^ ^77^, ^79^ ^]^

The above analyses recognized that CBUs play significant roles in the nucleation process. In case the CBUs originate from the degraded parent zeolite, the nucleation process should be easier than that using an amorphous silica source with the assistance of a template. Therefore, the zeolite growth will be accelerated benefiting from the more nucleis, which also suppress the generation of impurity phase and promote nanoengineering of zeolite crystal.

In 2017, Zhu et al. first synthesized Sn‐Beta zeolite through a structural reconstruction synthesis approach.^[^ ^38^ ^]^ In detail, aluminosilicate Beta zeolite was dealuminated deeply and degraded in an alkaline TEAOH solution. The fragments of Al‐free Beta and Sn species were then self‐assembled into a Sn‐Beta framework with the assistance of TEA^+^ and F^−^ ions (Figure 8C). The total crystallization process was completed within a relatively narrow time‐window of 1 h, which was two orders of magnitude shorter than the traditional strategy. Moreover, the resultant Sn‐Beta zeolite possessed nanosized crystals from 30 to 50 nm with incredibly high framework Sn content (Si/Sn = 33). Despite the small crystal size of Sn‐Beta prepared by structural reconstruction, its hydrophobicity was comparable to that of bulky Sn‐Beta‐F hydrothermally synthesized by conventional fluoride‐mediated method. Those signals in UV‐Raman spectra, attributed to primary building subunits of Beta framework such as four‐, five‐, and six‐membered rings, appeared in the initial gel, which was not observed when using amorphous TEOS as a silica source. This result illustrated that to some degree CBUs promoted nucleation and crystal growth.^[^ ^38^ ^]^ Nanosized Ta‐Beta (20–40 nm) zeolite with isolated framework Ta^5+^ ions (Si/Ta molar ratio of 52) and Au‐loaded Sn‐Beta (Au/Sn‐Beta, 30–50 nm) catalyst have been subsequently synthesized by this structural reconstruction.^[^ ^86^ ^]^

Subsequently, this method was applied to prepare hierarchical Sn‐Beta material with the mesoporous SDA of polydiallydimethylammonium chloride (PDADMAC).^[^ ^87^ ^]^ The adding amounts of PDADMAC in the synthesis gel greatly affected textural properties, in particular mesoporous volume. In addition, Sn ions were incorporated Beta zeolite framework, existing in the configuration of both open and closed sites. This was then extended to the synthesis of metallosilicates with MFI‐type topology (TS‐1, ZSM‐5, Sn‐MFI, Fe‐MFI, Nb‐MFI).^[^ ^88^ ^]^ Gripper‐like silicon species with rich Si‐OH groups (mainly in Q^2^ groups as well as Q^3^ groups) reacted with metal ions in the synthesis gels, forming stable and isolated Si‐O‐metal groups, which were well‐preserved during the subsequent transformations. This method was also capable of suppressing the production of extra‐framework metal species and exclusive framework metal ions were noticed.^[^ ^88^ ^]^ In a word, gripper‐like silicon species played an important role in heteroatom incorporation and uniform distribution.

Sn‐Beta was post‐treated through steam‐assisted crystallization with the aid of NH_4_F and tetraethylammonium bromide (TEABr), which was also similar to the structural reconstruction approach.^[^ ^89^ ^]^ First, the NH_4_F solution dissolved the crystalline zeolite. Second, TEABr acted as the recrystallization agent of the zeolite framework, healing the framework defects. The treatment process was able to tune the hydrophobicity and morphology of Sn‐Beta zeolite.^[^ ^89^ ^]^ Hydrophilic Ti‐Beta zeolite with Ti‐rich exterior was also obtained via a dissolution‐recrystallization mechanism by treating crystalline Ti‐Beta zeolite with TEAOH solution at high temperature.^[^ ^90^ ^]^ Very recently, Ma et al. proposed an improved structural reconstruction (i.e., in situ structural reconstruction) to prepare hierarchical Ti‐Beta zeolite.^[^ ^91^ ^]^ In this case, all the raw materials containing siliceous Beta, TEAOH, NH_4_F, and titanium butoxide (TBOT) were one‐pot added in an autoclave, leaving out the dissolution procedure in the structural reconstruction method. TEAOH played dual roles of etching effect as the base source and reestablishing zeolite framework as OSDA (Figure 8D).^[^ ^91^ ^]^ The synthesis methodology of Ti‐Beta zeolite is potentially and promisingly expanded to the preparation of Sn/Zr‐Beta zeolite.

**Figure 9:**
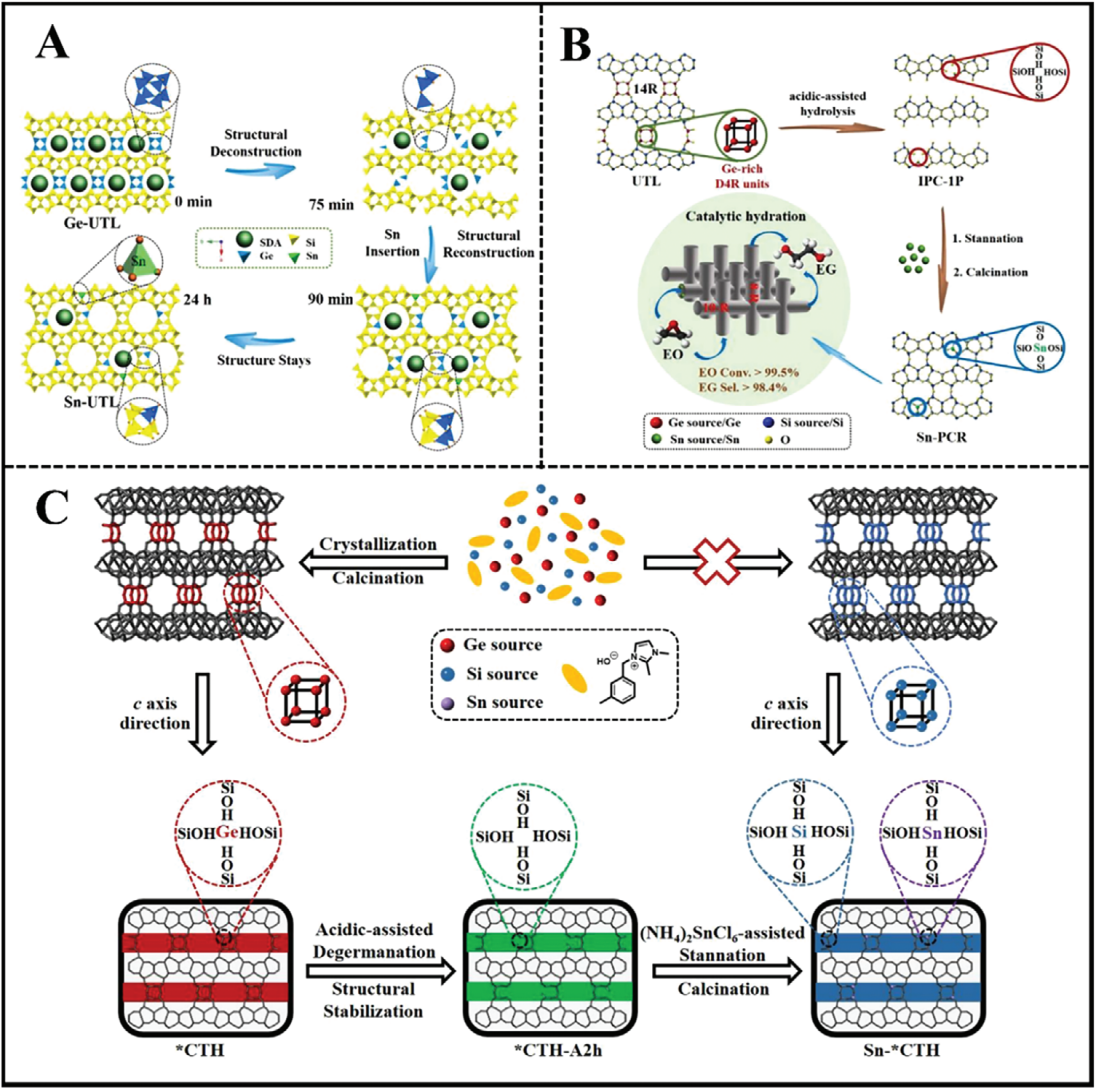
A) Post‐synthesizing extra‐large‐pore Sn‐UTL zeolite through structural rearrangement and isomorphous substitution. Reproduced with permission.^[^ ^92^ ^]^ Copyright 2016, American Chemical Society. B) Preparation of promising shape‐selective ethylene oxide hydration catalyst Sn‐PCR through structural rearrangement and isomorphous substitution. Reproduced with permission.^[^ ^93^ ^]^ Copyright 2022, Wiley‐VCH. C) The post‐synthesis of extra‐large pore stannosilicates Sn‐*CTH with high Sn content through acid‐assisted degermination and subsequent Sn incorporation with (NH_4_)_2_SnCl_6_. Reproduced with permission.^[^ ^94^ ^]^ Copyright 2023, Elsevier.

Liu et al. took advantage of a local structural rearrangement strategy to obtain extra‐large‐pore Sn‐UTL zeolite with intersecting 12‐ and 14‐MR pore channels.^[^ ^92^ ^]^ As‐synthesized UTL zeolite suffered from diluted acid treatment, whose framework structure underwent a rapid structural collapse initially and then reassembled into a stable zeolite framework. The most seriously collapsed intermediate structure was further stannated at 190 °C in an acidic medium. After a period of time, the UTL framework structure was reconstructed and meanwhile, Sn ions were isomorphously substituted into the zeolite framework through the interaction between Sn^4+^ ions and hydroxyl nests in company with the removal of Ge and/or Si species in the layer (**Figure** **9**A).^[^ ^92^ ^]^ In 2022, the same group also used a simple acid treatment strategy to remove all the interlayer D4R units, converting the 3D UTL germanosilicate into a 2D‐layered IPC‐1P intermediate. Isomorphous incorporation of tetrahedrally coordinated Sn active centers and subsequent calcination prepared Sn‐PCR zeolite (Figure 9B).^[^ ^93^ ^]^ Recently, they also demonstrated that OSDA‐free *CTH germanosilicate preserved the original structure under an acid medium for more than 2 h irrespective of the Ge leach.^[^ ^94^ ^]^ The total hydrolysis process created abundant hydroxyl nests and Sn‐*CTH stannosilicates with extra‐large 14‐MR pores were post‐prepared by isomorphous incorporation of tin ions (Figure 9C).^[^ ^94^ ^]^

### 2.2 Top‐down Approaches

Considering the shortcomings of the direct hydrothermal synthesis of Sn/Zr‐zeolites, such as long crystallization time, limited framework metal loading, large crystal size, and usage of toxic fluoride,^[^ ^95^ ^]^ creative top‐down methods were proposed involving two subclasses of direct metalation and demetallation‐metalation.

#### 2.2.1 Direct Metalation

##### Interlayer‐Expanded Method

**Figure 10:**
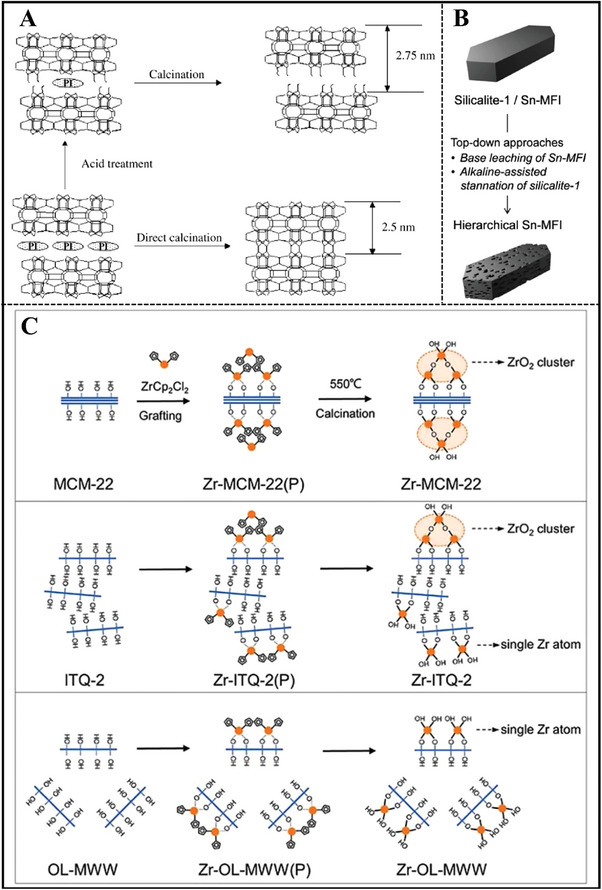
A) Possible scheme for the formation of Ti‐YNU‐1 and 3D Ti‐MWW, as well as their structures. Reproduced with permission.^[^ ^97b^ ^]^ Copyright 2004, Wiley‐VCH. B) Top‐down strategies followed for the preparation of hierarchical Sn‐containing MFI zeolites. Reproduced with permission.^[^ ^114b^ ^]^ Copyright 2014, Royal Society of Chemistry. C) Direct grafting synthesis of bi‐functional Zr‐Al‐MWW zeolites. Reproduced with permission.^[^ ^112b^ ^]^ Copyright 2022, Elsevier.

2D‐layered zeolite lacks the four T‐atoms connection between bridging units of lamellar sheets. This feature endows it with great structural diversity.^[^ ^96^ ^]^ Generally speaking, silylation agents or metal salts are used to link layer silicates, resulting in targeted 3D interlayer expansion zeolites (Me‐IEZs) (**Figure** **10**A).^[^ ^28^, ^97^ ^]^ Moreover, the incorporation of metal ions not only expands the pore size but also makes siliceous zeolite functionalized due to the isolated metal ions‐derived Lewis acid sites.

Bian et al. replaced the conventional silylating agent with bis(2,4‐ pentanedionate)‐dichlorotin [Sn(acac)_2_Cl_2_)] salt, connecting the layer of the COK‐5 precursor.^[^ ^98^ ^]^ The interlayer distance was increased after Sn^4+^ ions were inserted into the interlayer. The resultant crystalline Sn‐COK‐5 with both Brønsted and Lewis acid sites was applied in the transformation of glucose to levulinic acid.^[^ ^98^ ^]^ Subsequently, Gies et al. also performed the interlayer expansion RUB‐36 (Me‐IEZ‐RUB‐ 36) zeolites with Al‐, Ti‐, Sn‐, Fe‐, Eu‐containing linker sites.^[^ ^99^ ^]^ Rietveld refinement of the XRD data demonstrated the structure of Me‐IEZ‐RUB‐36 was well preserved after calcination.^[^ ^99^ ^]^ This method of interlayer expansion was extended to the generalized synthesis of Me‐IEZ‐RUB‐36 (Me = Sn, Fe, Zn, Co) in an acidic solution using corresponding metal acetylacetate salt.^[^ ^100^ ^]^

##### Atom‐Planting Method

The famous atom‐planting method was first declared for the incorporation of metal ions into zeolite.^[^ ^101^ ^]^ In 1984, Al ions were incorporated into the ZSM‐5 framework by AlBr_3_ or AlCl_3_ vapor treatment.^[^ ^102^ ^]^ ZSM‐11 zeolite was also smoothly aluminated with AlCl_3_ vapor at 500 °C.^[^ ^103^ ^]^ Mechanism analysis verified that the metal ions interacted with defect sites involving external and/or internal silanols in the zeolites. Zr‐MCM‐22 and Zr‐MCM‐56 were also prepared by a simple impregnation method.^[^ ^104^ ^]^ Additionally, authors also claimed that Zr^4+^ ions were present in a tetrahedrally coordinated configuration by UV–vis and XPS analyses. Furthermore, metalation could proceed even for the starting zeolite without defect sites in fluoride‐containing solution due to the etching effect of fluoride on silica.^[^ ^2^, ^105^ ^]^ Motivated by this, in 1995, Skeels et al. directly stannated Al‐Y zeolite with a Si/Al molar ratio of 2.5–7.0 in (NH_4_)_2_SnF_6_ solution.^[^ ^106^ ^]^ Active zeolite catalyst was yielded as a result of the isomorphous substitution of Sn to Al within Y zeolite. However, the position, coordinated state, and micro‐environment of Sn ions were unknown due to the limited analysis techniques. Treating Al‐ZSM‐5 (Si/Al = 19) in (NH_4_)_2_ZrF_6_ solution enabled the successful synthesis of Zr‐Al‐ZSM‐5 zeolite with tetrahedrally coordinated Zr^4+^ ions.^[^ ^107^ ^]^ Similarly, extra‐large‐pore Sn‐PLS‐3 zeolite with 14 × 12‐MR was also post‐synthesized using interlayer‐expanded ECNU‐9 zeolite in NH_4_F solution. Sn^4+^ ions were inserted into the hydroxyl nests generated from the corrosion of F^−^ ions to Si^4+^ ions in zeolite sheets, forming framework Sn sites.^[^ ^108^ ^]^

**Figure 11:**
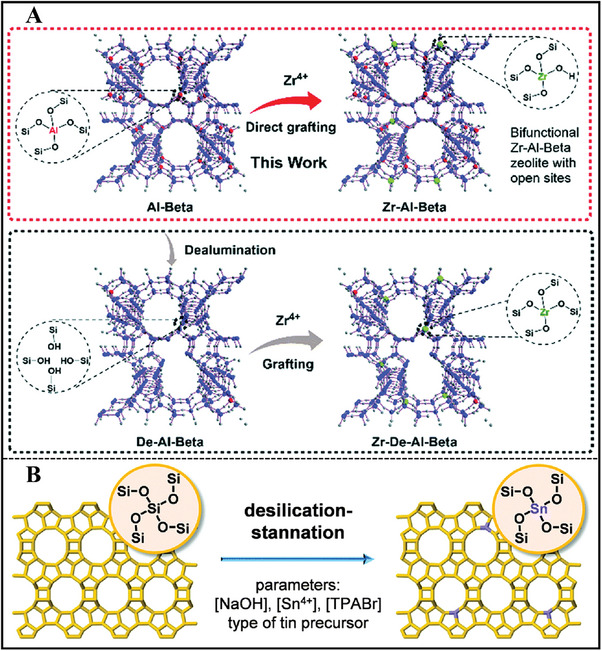
A) One‐step strategy and two‐step strategy for the post‐synthesis of Zr‐Al‐Beta and Zr‐De‐Al‐Beta. Reproduced with permission.^[^ ^110^ ^]^ Copyright 2019, Royal Society of Chemistry. B) Strategy followed to prepare Sn‐BEA by alkaline‐assisted stannation. Reproduced with permission.^[^ ^115^ ^]^ Copyright 2016, Royal Society of Chemistry.

Beta zeolite consists of the intergrowth of polymorphs A and B, creating abundant Si‐OH groups in the framework.^[^ ^109^ ^]^ These defect sites can serve as anchoring sites to introduce heteroatoms into the Beta framework. Recently, Li et al. post‐treated Al‐Beta zeolite in Zr(NO_3_)_4_·5H_2_O solution with ethanol as solvent. This one‐step strategy formed bifunctional Zr‐Al‐Beta zeolite with open Zr (IV) sites as well as framework Al‐derived Lewis and Brønsted acid sites, respectively (**Figure** **11**A).^[^ ^110^ ^]^ Compared with traditional Zr‐De‐Al‐Beta zeolite by a two‐step step, this Zr‐Al‐Beta zeolite demonstrated fewer silanol groups and relatively high hydrophobicity. Subsequently, the same group conducted a mild alkaline treatment of Zr‐Al‐Beta zeolite for tailoring its active sites. It was noticed that Brønsted and Lewis acid sites can be transformed mutually and their contents were weakened significantly through the simple alkaline treatment. The acidity and textural properties were affected by the type of alkali metal ions and their concentration, which were correlated to their catalytic performances.^[^ ^111^ ^]^ This method was also used to prepare Sn/Ni/Fe‐Beta and bifunctional Zr‐Al‐MWW zeolites (Figure 10C).^[^ ^112^ ^]^

##### Alkaline‐Assisted Metalation

During the past decade, numerous investigations have been documented for releasing the diffusion constrains of zeolites. Alkali treatment strategy has proved to be an efficient means to creat mesoporosity and improve diffusion properties.^[^ ^113^ ^]^ In addition, desilication under alkaline media also induced a large number of silanol nests, which played a significant role in accommodating metal species. As a result, Sn ions were incorporated into MFI zeolite using an alkaline‐assisted metalation strategy.^[^ ^114^ ^]^ High‐silica MFI zeolite (Si/Al = 961) was treated under NaOH solution with Sn precursor. However, this stannation method produced a large amount of extra‐framework SnO_2_ species, which was different from the homogeneous distribution of Sn for hydrothermally synthesized Sn‐MFI zeolite material (Figure 10B).^[^ ^114^ ^]^ This post‐synthetic method was also adopted for Beta zeolite (Figure 11B).^[^ ^115^ ^]^ Dapsens et al. selected high‐silica Beta zeolite with a Si/Al molar ratio of 220 as the stannated parent and meanwhile, tetrapropylammonium bromide (TPABr) was used as a protective additive to keep framework crystallinity. The authors studied the influence of the following parameters (concentration of alkaline medium, TPABr amount, the type and contents of Sn precursor) on the catalytic performance during the isomerization of glyoxal in water. Sn‐Beta zeolite catalyst was optimized by adjusting the abovementioned factors and stannated conditions were as follows, SnSO_4_ as metal source, 0.3 mol L^−1^ NaOH, 0.4 mol L^−1^ SnSO_4_, and 0.02 mol L^−1^ TPABr. The TOF of this optimal Sn‐Beta catalyst was still inferior to that of the reference fluoride‐mediated Sn‐Beta zeolite, which may be ascribed to the lower amounts of Sn framework sites and greater surface hydrophobicity over alkaline‐assisted Sn‐Beta zeolite.^[^ ^115^ ^]^ Additionally, the alkaline‐assisted metalation strategy easily constructed metallosilicates with mesoporosity structure, which favored the diffusion of bulky organics and improved the accessibility of reaction substrates with active sites in zeolite crystal.

#### 2.2.2 Demetallation‐Metalation

Since direct metalation encounters the upper‐limited metal active sites, the precursor with rich silanol nests is necessary. Hence, a two‐step strategy (demetallation‐metalation) was born. Aluminosilicate was performed by dealumination treatment, forming rich silanol nests inside the zeolite framework before further metalation. The silanol nests are able to react with metal ions, obtaining metallosilicates.^[^ ^116^ ^]^ The dealuminated parameters including acid concentration, treatment time as well as Al content of Al‐zeolite affect the dealuminated degree. According to the phase state of the metalation process, three main approaches are distinguished, CVD, liquid grafting, and solid‐state ion‐exchange (SSIE).

**Figure 12:**
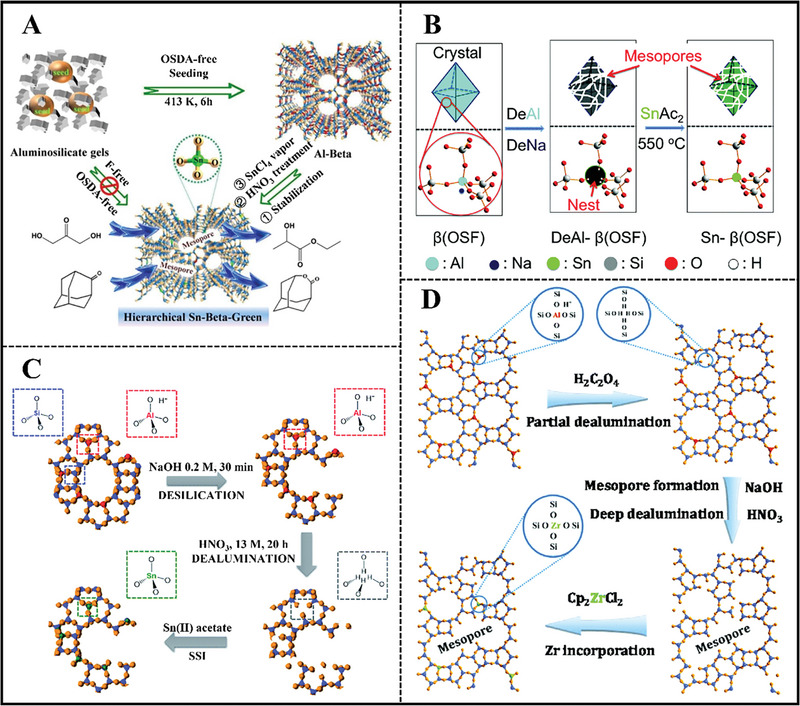
A) Al coordination states depend on the steaming conditions of Beta zeolite. Reproduced with permission.^[^ ^120^ ^]^ Copyright 2018, Elsevier. B) Illumination of the OSDA‐free synthetic processes of hierarchically porous Sn‐β zeolites. Reproduced with permission.^[^ ^140b^ ^]^ Copyright 2017, Royal Society of Chemistry. C) Postsynthetic desilication procedure for mesopores creation in the zeolite Beta using NaOH as desilication agent (step 1), and subsequent formation of stannosilicate via solid state stannation (steps 2 and 3). Reproduced with permission.^[^ ^143c^ ^]^ Copyright 2016, Royal Society of Chemistry. D) Schematic representation of the intra‐crystalline mesopore formation and tetrahedral Zr(IV) incorporation. Reproduced with permission.^[^ ^143a^ ^]^ Copyright 2013, Royal Society of Chemistry.

For the CVD method, highly dealuminated Beta zeolites were treated with anhydrous SnCl_4_ vapor.^[^ ^117^ ^]^ The crystal size of Sn‐Beta zeolite was 60–80 nm and the Sn contents reached the maximum values of 0.5 mmol g^−1^ (corresponding to Si/Sn of 35), which was more efficient in comparison with the traditional fluoride‐mediated Sn‐Beta zeolite. Whereas, extra‐framework Sn species were observed for Sn‐Beta with high Sn amounts above 5 wt.%. In spite of the relatively high hydrophilicity, excellent diffusion properties derived from nanosized crystal and high Sn amounts endowed Sn‐Beta with superior catalytic performance in the Baeyer–Villiger oxidation of 2‐adamantanone than traditional Sn‐Beta.^[^ ^117^ ^]^ Jin et al. used cationic ammonium‐modified chitosan to hydrothermally synthesize mesoporous Beta zeolite.^[^ ^118^ ^]^ Sn ions were incorporated into Beta through the CVD method, affording Sn (IV) amounts of 1.1 wt,%. The presence of intercrystalline/interparticle mesoporosity enhanced the accessibility of active sites by guest organics.^[^ ^118^ ^]^ Hierarchical Beta zeolite was obtained in the presence of polydiallyldimethylammonium chloride, which was further dealuminated and stannated with SnCl_4_ vapor.^[^ ^119^ ^]^ Hierarchical Sn‐Beta zeolite was also achieved via the interaction between SnCl_4_ vapor and hydroxyl nests in dealuminated Beta zeolite derived from an organotemplate‐free route (**Figure** **12**A).^[^ ^120^ ^]^ The mesoporosity was generated from the dealuminated process.^[^ ^120^ ^]^ Al‐Beta (Si/Al = 15) or B‐Beta (Si/B = 11) were used as the starting materials, both generating Sn‐Beta zeolite with Sn amounts of 2.59 and 4.64 wt.%, respectively.^[^ ^121^ ^]^ Nonetheless, three kinds of Sn species were found in two Sn‐Beta zeolites, that is, bulk SnO_2_ species, framework, and extra‐framework Sn species. Subsequently, commercial Al‐Beta was subjected to HNO_3_ treatment.^[^ ^122^ ^]^ And acid‐treated conditions affected the dealuminated degree. After Sn incorporation by the CVD method, bifunctional Sn‐Al‐Beta with both Sn‐derived Lewis acid sites and Al‐based Brønsted acid sites was realized.^[^ ^122^ ^]^

**Figure 13:**
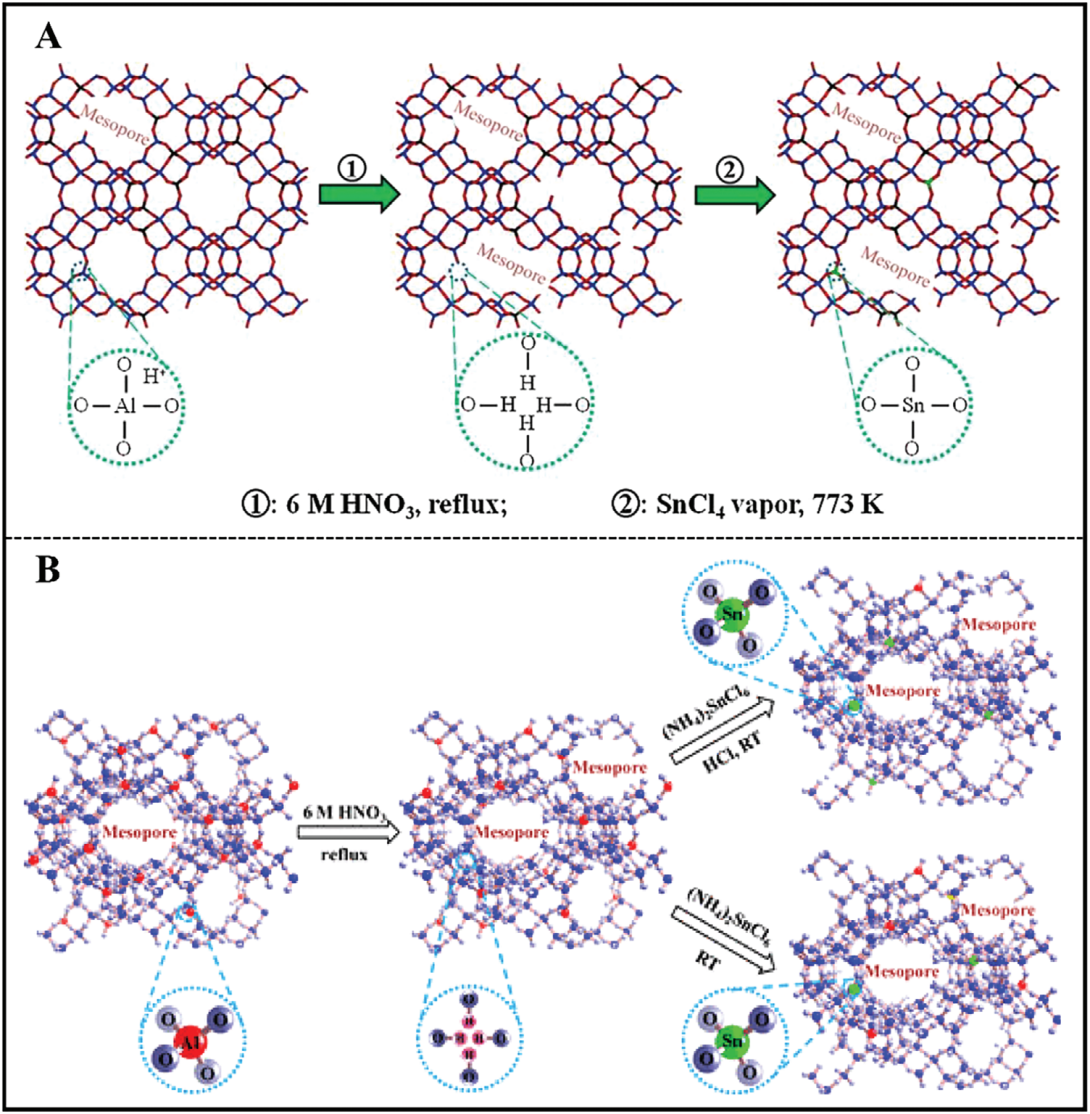
A) Two‐step post‐synthetic strategy for the preparation of Sn‐Y. Reproduced with permission.^[^ ^123a^ ^]^ Copyright 2016, Elsevier. B) Two‐step strategy for the synthesis of Sn‐Y. Reproduced with permission.^[^ ^127^ ^]^ Copyright 2016, American Chemical Society.

Sn‐Y was also prepared by reacting SnCl_4_ vapor with dealuminated ultra‐stable Y zeolite (**Figure** **13**A).^[^ ^123a^ ^]^ The amounts and coordinated state of Sn‐incorporated was closely related to the acid‐treated time. This methodology was also suitable for the synthesis of Sn‐MSE stannosilicate zeolite.^[^ ^124^ ^]^

As for the liquid grafting strategy, the solvent has a prominent effect on the content and coordination states of the incorporated metal species located in zeolite frameworks.^[^ ^125^ ^]^ The dealuminated Beta zeolite was immersed in anhydrous SnCl_4_, forming Sn‐Beta zeolite with high isolated Sn loadings of 5 wt.%. With continuously increasing the Sn amounts, tin dioxide appeared in the extra‐framework position.^[^ ^125a^ ^]^ When the Sn contents reached up to 25 wt.%, Sn‐Beta zeolite exhibited poor catalytic performance because of the existence of abundant SnO_2_ species.^[^ ^125b^ ^]^ Despite the relatively low Sn content of 2 wt.%, Sn‐Beta obtained via grafting in water always exhibited partially extra‐framework Sn species.^[^ ^125c^ ^]^ The usage of 2‐propanol as solvent led to the low isolated Sn sites resulting from the competitive adsorption of solvent to Sn species on the hydroxyl nests.^[^ ^125d,e,f^ ^]^ Solvents with low affinity to the silanol nests, such as dichloromethane, contributed to the Sn ions grafting in hydroxyl groups, giving high Sn amounts up to 6 wt.%.^[^ ^125f^ ^]^ Sn‐Y zeolite containing Al ions was also obtained by two different procedures, thermal grafting in isopropanol and trimethylamine‐mediated chemical grafting in CH_2_Cl_2_.^[^ ^126^ ^]^ Zhu et al. reported that dealuminated Y zeolite was carried out by liquid grafting of (NH_4_)_2_SnCl_6_ under an acidic medium, in order to obtain Sn‐Y zeolite. The addition of HCl in solution facilitated the removal of the residual Al species in Y zeolite and the subsequent insertion of Sn (IV) (Figure 13B).^[^ ^127^ ^]^

Zr‐Beta and Zr‐Y were also derived from grafting ZrOCl_2_ˑ8H_2_O in water over dealuminated Beta and Y zeolites, respectively.^[^ ^128^ ^]^ Interestingly, Zr ions still existed in tetrahedral sites even when the Si/Zr molar ratio was as low as 20 for Zr‐Y zeolite.^[^ ^128a^ ^]^ The isolated Zr amounts in terms of Si/Zr molar ratio even achieved a low of 12.5 in Zr‐Beta zeolite.^[^ ^128^, ^129^ ^]^ Small crystal size, stronger Lewis acidity but high hydrophilicity were observed for grafting Zr‐Beta zeolite compared with hydrothermally fluoride‐mediated Zr‐Beta.^[^ ^130^ ^]^ Partially dealuminated USY and Beta were used as parents to construct bifunctional Zr‐Al‐Y and Zr‐Al‐Beta zeolite, respectively, in the solvent of water.^[^ ^131^ ^]^ Melero et al. found that using Zr(NO_3_)_4_ as a metal source rather than ZrOCl_2_ and a suitable water concentration during the grafting process was appropriate for the dispersion of Zr species within Zr‐Al‐Beta zeolite.^[^ ^132^ ^]^ The combination of acid and base treatment for Al‐containing zeolite can fuel the preparation of hierarchical Zr‐Beta and Zr‐Y zeolites.^[^ ^128^, ^133^ ^]^ Taking MWW‐type borosilicate zeolite precursor ERP‐1P with Zn(NO_3_)_2_ in an acidic solution contributed to the following merits, (I) removal of framework B, (II) formation of silanol nests and (III) generation of delamination structure.^[^ ^134^ ^]^ Then heteroatoms (Sn, Zr, Ti, Hf, Nb, Ta) substituted MWW zeolite with delaminated structure was synthesized by reacting silanol nests with the organic metal precursor in *n*‐BuOH.^[^ ^134^ ^]^ Stabilization of BEC germanosilicate with TEOS followed by modification with ZrOCl_2_ in DMSO produced Zr‐BEC zeolite with high stability and Lewis acidity.^[^ ^135^ ^]^

With respect to the SSIE method, dealuminated zeolite is mixed with a metal source, which is then grounded mechanically and further calcined at high temperatures. This means is simple and potential for large‐scale applicability. Normally, the organometallic compound is adopted as a metal source and the maximum Sn content was ≈10 wt.%, corresponding to the Si/Sn molar ratio of 16.^[^ ^136^ ^]^ Hammond et al. reported that this Sn‐Beta with the high Sn content still exhibited tetrahedral Sn species confirmed by UV–vis and Raman spectra.^[^ ^136^ ^]^ However, the same group claimed that an extra‐framework nuclearity Sn cluster was formed for Sn‐Beta with the Sn content of 10 wt.%, proved by ^119^Sn MAS NMR spectra.^[^ ^137^ ^]^ Recently, Dai et al. used more advanced techniques to characterize the coordinated state of Sn in Sn‐Beta zeolite.^[^ ^138^ ^]^ They found the calcination atmosphere influenced significantly the micro‐environment of Sn in Sn‐Beta‐SSIE. When Sn‐Beta (5 wt.% Sn) was calcinated at 550 °C under vacuum prior to calcination at the same temperature under air conditions, Sn species was mainly incorporated into Beta zeolite framework. Once omitting the vacuum calcination process, a SnO_x_ cluster in the size of 0.5–2 nm was clearly observed. Additionally, both framework Sn sites and extra‐framework SnO_x_ clusters can generate Lewis acidity. The SnO_x_ clusters in the diameter of 0.5–2 nm presented comparable Lewis acidity compared to the framework‐substituted Sn species.^[^ ^138^ ^]^

This SSIE strategy has been extended to the preparation of Zr‐Beta,^[^ ^139^ ^]^ Sn‐Beta (Figure 12B),^[^ ^125^, ^139^, ^140^ ^]^ Ti‐Beta,^[^ ^139a^ ^]^ Zr‐SCM‐1 (delaminated MWW),^[^ ^141^ ^]^ bimetallic Pb/Sn‐Beta.^[^ ^142^ ^]^ SSIE method was also applied to synthesize hierarchical Sn/Zr‐zeolites by combining acid and base treatment or using hierarchical parent Al‐zeolite (Figure 12C,D).^[^ ^143^ ^]^ Synthetic methods for the preparation of Sn‐zeolites were summarized in **Table** **1**.

**Table 1:** Synthetic methods for the preparation of Sn‐zeolites.

No.	Topology	Method	Sn precursor	Seed	F^−^ ions	Tem. [°C]	Time [d]	Si/Al or Si/B	Si/Sn	References
Bottom‐up approaches										
1	MFI	HS	SnCl_4_⋅5H_2_O	No	No	160	1–2	Al free	25–28	[12]
2	MFI	HS	SnCl_4_⋅5H_2_O	No	Yes	200	6–7	Al free	15	[12]
3	MFI	HS	SnCl_4_⋅5H_2_O	No	No	160	3–7	Al free	15–150	[13]
4	MFI	HS	EDTA‐Sn	No	No	200	3	Al free	50–100	[14]
5	MFI	HS	EDTA‐Sn	No	No	200	3	80–240	67	[14]
6	MFI	HS	Sn‐but	No	No	150–160	10–20	Al‐free	125–250	[17b]
7	MWW	HS	SnCl_4_⋅5H_2_O	No	No	170	7	70	40–200	[31]
8	MWW	HS	SnCl_4_⋅5H_2_O	No	No	175	3	>120	52–54	[32]
9	MWW	HS	SnCl_4_⋅5H_2_O	No	No	150	5	Al free	100–150	[33]
10	*BEA	HS	SnCl_4_⋅5H_2_O	Yes	No	142	10	>4000	54.5	[37]
11	*BEA	HS	SnCl_4_⋅5H_2_O	Yes	No	142	10	28.5	78.8	[37]
12	*BEA	HS	SnCl_4_⋅5H_2_O	Yes	Yes	140	20	Al free	120	[37]
13	*BEA	HS	SnCl_4_⋅5H_2_O	Yes	Yes	140	2	Al free	126	[43]
14	*BEA	HS	SnCl_4_⋅5H_2_O	No	Yes	140	4–60	Al free	100–400	[44b]
15	*BEA	HS	SnCl_4_⋅5H_2_O	Yes	Yes	150	5–9	Al free	91–183	[47]
16	*BEA	HS	SnCl_4_⋅5H_2_O	Yes	No	140	7	Al‐free	39–158	[51]
17	CHA	HS	SnCl_4_⋅5H_2_O	No	Yes	150	2	Al free	60–70	[58]
18	MEL	HS	SnCl_4_⋅5H_2_O	No	No	160	2.5	Al free	41–102	[59]
19	MTW	HS	SnCl_4_⋅5H_2_O	No	No	160	5	Al free	73–177	[61]
20	MFI	DGC	SnCl_4_⋅5H_2_O	No	No	110‐200	0.2–1.1	Al free	51–140	[67]
21	*BEA	DGC	SnCl_4_⋅5H_2_O	No	Yes	180	0.1–2.5	Al free	64–490	[69]
22	*BEA	DGC	Sn‐but	Yes	No	140	5	Al‐free	125	[36]
23	CHA	IZT	Sn‐Al‐FAU	Yes	No	170	2	18	41	[76]
24	*BEA	IZT	SnCl_4_⋅5H_2_O	Yes	Yes	140	1–3	Al free	63–203	[77]
25	*BEA	IZT	SnCl_4_⋅5H_2_O	Yes	No	140	1–15	511–514	81–198	[79a]
26	*BEA	SRS	SnCl_4_⋅5H_2_O	No	Yes	140	0.04	>1900	33–152	[38]
27	*BEA	SRS	SnCl_2_⋅2H_2_O	No	Yes	140	0.5	>1800	148–150	[87]
28	MFI	SRS	SnCl_2_⋅2H_2_O	No	No	170	2	Al free	141	[88]
29	UTL	SRS	SnCl_4_⋅5H_2_O	No	No	190	24	Al free	100–232	[92]
30	PCR	SRS	(NH_4_)_2_SnCl_6_	No	No	25	24	Al free	13–123	[93]
31	*CTH	SRS	(NH_4_)_2_SnCl_6_	No	No	25	24	Al free	41–107	[94]
Top‐down approaches										
32	COK‐5	IEM	Sn(acac)_2_Cl_2_	No	No	180	1	32–35	225–579	[98]
33	RUB‐36	IEM	Sn(acac)_2_Cl_2_	No	No	157	2	NA	22	[99]
34	PLS‐3	APM	SnCl_4_⋅5H_2_O	No	Yes	50	5	NA	47–144	[108]
35	*BEA	APM	SnCl_2_⋅2H_2_O	No	No	80	6	NA	NA	[112a]
36	MFI	ASM	Sn(SO_4_)_2_	No	No	65	0.5	961	76	[114a]
37	*BEA	ASM	Sn(SO_4_)_2_	No	No	65	0.5	220	56–298	[115]
38	*BEA	CVD	SnCl_4_	No	No	200–600	0.04	>1700	35–48	[117]
39	*BEA	CVD	SnCl_4_	No	No	500	0.08	>2000	162	[119]
40	*BEA	CVD	SnCl_4_	No	No	500	0.06	217	83	[120]
41	FAU	CVD	SnCl_4_	No	No	500	0.08	6–128	75–823	[124a]
42	MSE	CVD	SnCl_4_	No	No	500	0.08	290	78	[124]
43	*BEA	LG	SnCl_4_	No	No	100	NA	>1500	37–68	[125a]
44	*BEA	LG	SnCl_4_⋅5H_2_O	No	No	25	1	NA	96–391	[125c]
45	*BEA	LG	SnCl_4_⋅5H_2_O	No	No	110	0.29	>1500	30–457	[125f]
46	FAU	LG	(NH_4_)_2_SnCl_6_	No	No	25–170	1	18–253	42–190	[127]
47	*BEA	SSIE	Tin(II) acetate	No	No	RT	0.01	>950	16–32	[136]
48	*BEA	SSIE	Tin(II) acetate	No	No	RT	0.007	>500	15–75	[137]
49	*BEA	SSIE	Tin(II) acetate	No	No	RT	0.02	330	23	[140b]

HS: hydrothermal synthesis; IZT: interzeolite transformation; SRS: structural reconstruction strategy; IEM: interlayer‐expanded method; APM: atom‐planting method; ASM: alkaline‐assisted metalation; DIM: direct incorporation for mesoporous silicates; LG: liquid grafting; NA: not available; RT: room temperature; EDTA‐Sn: ethylenediaminetetraacetic acid tin; Sn(acac)2Cl2: bis(2,4‐pentanedionate)‐dichlorotin; Sn‐but: tin(IV) tert‐butoxide.

## 3 Catalytic Applications of Sn‐ and Zr‐Zeolites

**Figure 14:**
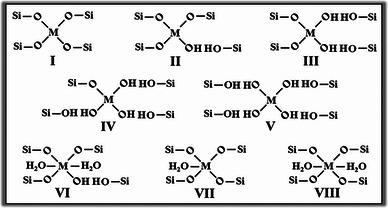
Plausible configurations for dehydrated and hydrated M (M = Sn or Zr) sites in silica frameworks.

The electrons of incorporated Sn/Zr atoms are able to form covalent bonds with oxygen atoms in a silicate framework. When the metal ions are coordinated with four framework oxygen atoms, ‘closed’ site is generated (**Figure** **14**, species I). If the metal ions are coordinated to three framework oxygen atoms and one hydroxyl oxygen atom, this configuration is designated as ‘open’ site (Figure 14, species II). Thus, the covalent electrons are pushed toward the oxygen atom because of the different electronegativity. In this regard, the framework Sn (IV) and Zr (IV) atoms are in the coordinated unsaturated state, which exhibits Lewis acidity.^[^ ^8^ ^]^ Moreover, the oxygen atoms in the framework position present a weak basic site. Consequently, Si‐O‐metal groups can serve as acid‐base pairs during the catalytic reactions.^[^ ^144^ ^]^ The isolated metal‐derived Lewis acid sites are able to accept electron pairs from electron‐rich groups, causing chemical activation. In this sense, carbonyl groups are activated by the transfer of an electron mainly in p_y_ and p_z_ orbitals of the oxygen atom to the empty orbitals of the metal atom (mainly the π(CO) in the Sn LUMO orbitals) and further a back‐donation of the Sn HOMO orbitals to antibonding π^*^(CO) orbitals. This donation and further back‐donation mechanism lead to positive charge density in the carbonyl group and an increase in C─O bond length.^[^ ^145^ ^]^ The catalytic applications of Sn/Zr‐zeolites were systematically summarized in the fields of redox catalysis, Lewis acid catalysis, biomass catalysis, and catalysis support.

### 3.1 Redox Catalysis

#### 3.1.1 Baeyer–Villiger Oxidation

Baeyer and Villiger reported the Baeyer–Villiger (B–V) oxidation in 1899, which was applied in the conversion of ketones into esters or lactones.^[^ ^146^ ^]^ Initially, this reaction was conducted through a noncatalytic process with the oxidants of peracids or persulfate salts. This system produced useless byproducts inevitably, commonly resulting in a low product selectivity in particular when other function groups like C═C bond are present in reaction substrates.^[^ ^127^ ^]^ In order to reduce the adverse effects of organic oxidants, it is urgent to develop an efficient B–V oxidation process with environmentally benign oxidants such as hydrogen peroxide (H_2_O_2_). During the past decades, a series of catalysts have been developed to activate H_2_O_2_ including hydrotalcite,^[^ ^147^ ^]^ acidic anionic resins,^[^ ^148^ ^]^ aluminosilicates,^[^ ^148^ ^]^ germanosilicates,^[^ ^149^ ^]^ and titanosilicates.^[^ ^150^ ^]^ In 1996, titanosilicate TS‐1 was utilized to catalyze B–V oxidation of cyclohexanone and acetophenone. It was found that peroxide Ti species drove this reaction, but the acidic nature led to the formation of byproducts.^[^ ^150^ ^]^

**Figure 15:**
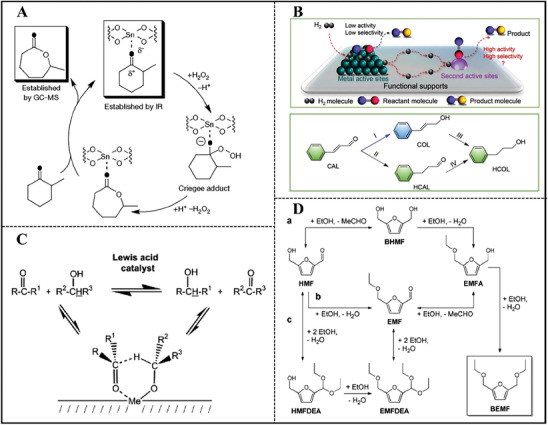
A) Catalytic cycle for the Baeyer‐Villiger oxidation, using hydrogen peroxide catalyzed by Sn‐zeolite beta. Reproduced with permission.^[^ ^40^ ^]^ Copyright 2001, Springer. B) (a) Proposed hydrogenation pathway on dual active sites enabled by hydrogen spillover. (b) Reaction steps of the CAL hydrogenation. *Source*: Reproduced with permission.^[^ ^158^ ^]^ Copyright 2021, Elsevier. C) MPV reaction pathways. R = alkyl or aryl; R^1^ and R^3^ = alkyl or hydrogen; Me = metal. *Source*: Reproduced with permission.^[^ ^161^ ^]^ Copyright 2003, Elsevier. D) Proposed reaction network for the coupled TH and etherification of HMF with ethanol. Reproduced with permission.^[^ ^164^ ^]^ Copyright 2014, Wiley‐VCH.

In 2001, Corma et al. claimed that stannosilicate Sn‐Beta was capable of catalyzing the B‐V oxidation of unsaturated ketones with H_2_O_2_, providing high conversion and selectivity (**Figure** **15**A).^[^ ^40^ ^]^ This reaction mechanism contained the following steps, (I) the adsorption of ketone to Sn sites, (II) the attack of H_2_O_2_ to carbon atom in the carbonyl group, (III) rearrangement, and (IV) desorption of lactone from zeolite catalyst to accomplish a full cycle. Subsequently, this same group observed that open Sn sites in fluoride‐assisted Sn‐Beta zeolite possessed superior catalytic properties for the conversion of cyclohexanone to lactone in comparison with closed Sn sites.^[^ ^151^ ^]^ Later, dozens of stannosilicates have been developed to catalyze B‐V oxidation reactions. In general, Sn‐Beta served as a benchmark catalyst. The relatively large‐sized 2‐adamantanone molecules are applied as the substrate to estimate the catalyst. The 10‐MR window opening imposes significant diffusing limitations for the 2‐adamantanone, but the 12‐MR in Sn‐Beta crystal is enough large for its penetration.^[^ ^123a^ ^]^ The polycyclic structure with high rigidity and stability for adamantanone afforded excellent selectivity as high as 98% in the B–V oxidation.^[^ ^123a^ ^]^ The lower selectivity of ≈80% is obtained when using cyclopentanone and cyclohexanone as substrates.^[^ ^152^ ^]^

In order to increase the diffusion property and enhance the accessibility of active sites, several methods are adopted to achieve this goal as follows, I) decreasing crystal size, II) designing hierarchical zeolite, and III) choosing large pore zeolite with opened channel system.

In order to decrease crystal size, Li et al. tested B–V oxidation of 2‐adamtanone with H_2_O_2_ over a nanosized Sn‐Beta‐CVD catalyst that was post‐treated via the CVD approach.^[^ ^117^ ^]^ In this reaction, the optimal solvent was chlorobenzene. Despite the relatively low hydrophobicity, the superiority of Sn‐Beta‐CVD zeolite in catalytic application still existed in comparison with the conventional fluoride‐mediated Sn‐Beta‐F due to the nanosized crystal size and high Sn contents of the former zeolite.^[^ ^117^ ^]^ The structural construction approach can prepare Sn‐Beta zeolite with a small crystal size in the range of 30–50 nm.^[^ ^38^ ^]^ This catalyst displayed extraordinary catalytic performance in the field of B–V oxidation of 2‐adamantanone in terms of turnover frequency (TOF) and space‐time‐yields (STY) than these Sn‐Beta materials synthesized via CVD and SSIE methods. Sn‐Beta zeolite synthesized through interzeolite transformation also possessed a small crystal size and exhibited excellent catalytic activity in the above‐mentioned reaction.^[^ ^77^, ^79^ ^]^

For designing hierarchical zeolite, hierarchical Beta zeolite was a potential stannated precursor for the post‐synthesis of hierarchical stannosilicate Sn‐Beta with microporous and mesoporous channel systems,^[^ ^118^ ^]^ which has verified that the hierarchical pore structure can improve catalytic performance in the B–V oxidation of 2‐adamantanone than ordinary Sn‐Beta zeolite. Sn‐MFI zeolite nanosheets with a thickness of ≈2 nm along the [010] direction performed more outstanding activity in the B–V oxidation of cyclic ketones in comparison to traditional 3D Sn‐MFI.^[^ ^17b^ ^]^ Specifically, thermally treating the Sn‐MFI sample at 1273 K for 4 h still preserved 95% of its original surface area and 100% of its oxidation activity.^[^ ^17b^ ^]^ Sn‐MCM‐56 (layered MWW zeolite) with a more open pore system offered large reaction space and external specific surface.^[^ ^31^ ^]^ As a result, the stannosilicate Sn‐MCM‐56 catalyst presented unique catalytic performance using large‐sized *tert*‐butyl hydroperoxide (TBHP) as an oxidant. The result was more efficient than 3D Sn‐MWW and traditional Sn‐Beta. Layered zeolites including but not limited to Sn‐UCB‐4,^[^ ^134^ ^]^ Sn‐DZ‐1,^[^ ^134^ ^]^ hierarchical Sn‐Beta,^[^ ^48^, ^49^, ^89^ ^]^ pillared Sn‐MWW,^[^ ^153^ ^]^ and interlayer‐expanded Sn‐PLS‐3,^[^ ^154^ ^]^ were also applied in the B–V oxidation. Moreover, the long‐term stability of the pillared Sn‐MWW was also tested by performing reactions over recycled and calcined catalysts. The catalyst retained its glucose isomerization activity after at least five runs. And the ordered layer structure and intra‐layer crystallinity of the used zeolite catalyst after the fifth run were still preserved as confirmed by XRD and Ar adsorption/desorption isotherms.

FAU topology structure zeolite possesses 3D 12‐MR channels and supercages, which is a promising stannated precursor for satisfying the purpose of preparing large pore catalysts with opening channel systems.^[^ ^123^, ^127^ ^]^ High‐quality Sn‐Y zeolite with a Si/Sn molar ratio of 75 was post‐synthesized by the CVD method by precisely controlling the acid‐treat time in the first step.^[^ ^123a^ ^]^ The catalytic activity was rather high over Sn‐Y zeolite in the oxidation of 2‐adamantanone and the conversion can achieve 97.5% within the reaction time of only 40 min, far outperforming these over hydrothermally synthesized Sn‐MFI, Sn‐Beta‐F, and Sn‐MWW materials.^[^ ^123a^ ^]^ In order to decrease the residual Al amounts in Sn‐Y zeolites (Si/Al = 43), a novel liquid grafting method was also developed by Zhu et al. The Si/Al molar ratio of obtained Sn‐Y was increased to ≈150 under optimal conditions.^[^ ^127^ ^]^ This Sn‐Y catalyst exhibited 87.1% lactone selectivity in the B–V oxidation of cyclohexanone with aqueous H_2_O_2_. The direct introduce of alkali metal ions into the reaction system was an effective approach to enhance the *ε*‐caprolactone selectivity.^[^ ^127^ ^]^ Sn‐UTL (14 × 12‐MR), Sn‐MSE (12 × 10 × 10‐MR) and UTL‐derived tin‐silica pillaring IPC‐1‐SnPI zeolites also demonstrated more efficient catalytic performance for the B–V oxidation, in particular, involving larger substrates (e.g. 2‐adamantanone) and oxidants (e.g. TBHP) than traditional stannosilicates (Sn‐MFI, Sn‐MWW, Sn‐Beta).^[^ ^92^, ^124^, ^152^ ^]^ Moreover, the deactivated zeolite catalyst can be nearly fully recovered to the initial level with a simple calcination under air atmosphere.

In addition to the abovementioned issues (diffusion performance and accessibility of active sites) affecting the catalytic performance of zeolite catalysts, hydrophobicity should also be taken into consideration. Conrad et al. prepared numerous Sn‐Beta zeolites with discrepant hydrophilicity via hydrothermal synthesis and post‐synthetic method,^[^ ^152b^ ^]^ which was applied in B–V oxidation of cyclohexanone with aqueous H_2_O_2_ to study the influence of hydrophilicity on catalytic activity. From the correlation between activity and hydrophilicity, authors declared that Sn‐Beta with moderate hydrophilicity exhibited the highest activity because this amount of silanol sites favored the interaction between reaction substrate and isolated Sn sites but minimized the competitive adsorption of solvent and/or products on the active sites.^[^ ^152b^ ^]^ Whereas, the difference in crystal size‐based diffusion performance was not considered in this contribution. The influence of diffusion performance and hydrophobicity/ hydrophilicity on catalytic performance in B‐V oxidation was investigated over post‐synthesized nanosized Sn‐Beta,^[^ ^117^ ^]^ hierarchical Sn‐Y,^[^ ^123^ ^]^ and microporous Sn‐MSE zeolites.^[^ ^124^ ^]^ Both excellent diffusion performance and high hydrophobicity had a positive effect on the catalytic property of B–V oxidation. Post‐synthesized Sn‐Beta and hierarchical Sn‐Y zeolite demonstrated outstanding diffusion properties and relatively low hydrophobicity in comparison with fluoride‐mediated Sn‐Beta (Sn‐Beta‐F) zeolite.^[^ ^117^, ^123^ ^]^ Nonetheless, diffusion performance may dominate this reaction under the present conditions, therefore resulting in a high catalytic activity of post‐synthesized Sn‐Beta and hierarchical Sn‐Y zeolites. Thus, Sn‐MSE with high hydrophobicity and comparable pore opening to Sn‐Beta‐F zeolite exhibited superior 2‐adamantanone conversion.^[^ ^124^ ^]^ Ion‐exchange of Sn‐Beta‐F with alkali metal (Li^+^, Na^+^, K^+^, Cs^+^) and ammonium cations led to an enhancement of *ε*‐caprolactone selectivity and 2‐adamantanone conversion in the B‐V oxidation of cyclohexanone and 2‐adamantanone, respectively, perhaps due to the fact that silanol groups were occupied by these cations, meanwhile improving the hydrophobicity.^[^ ^155^ ^]^ Recently, Zhu et al. post‐synthesized hierarchical Sn‐Y zeolite via a simple solid‐state ion‐exchange method, where Sn^4+^ ions were incorporated into the zeolite framework by reacting with silanol nests derived from the dealumination process. In comparison with other stannosilicate zeolites, the obtained Sn‐Y possessed excellent catalytic activity in B–V oxidation irrespective of using aqueous hydrogen peroxide or bulky tert‐butyl hydroperoxide as the oxidant. Structure‐performance relationship revealed that Lewis acid site was the catalytically active center and the catalytic activity was depended on zeolite poreengineering and hydrophobicity in an aqueous B–V oxidation system. The relatively high catalytic activity of Sn‐Y zeolite was ascribed to its excellent hydrophobicity and opened channel system.^[^ ^123b^ ^]^

#### 3.1.2 Meerwein–Pondorf–Verley Redox

In 1925, Meerwein–Schmidt and Verley discovered Meerwein–Ponndorf–Verley (MPV) reduction reaction for the first time,^[^ ^156^ ^]^ which is complementary to Oppenauer oxidation.^[^ ^157^ ^]^ In general, the hydrogenation reaction is conducted with H_2_ at elevated pressure and temperature, which needs high requirements for reaction equipment and causes high energy consumption, dangerous operation as well as low product selectivity (Figure 15B).^[^ ^158^ ^]^ With respect to MPV reduction, alcohol is used as a hydrogen source to selectively reduce the C═O bond of carbonyl compound under mild liquid conditions according to direct hydride transfer irrespective of the C═C double bond.^[^ ^159^ ^]^ Various homogeneous Lewis acid catalysts, for example, aluminum isopropoxide and lanthanide complex,^[^ ^160^ ^]^ can trigger the MPV reduction. However, the catalyst is difficult to separate.^[^ ^161^ ^]^ Subsequently, numerous heterogeneous catalysts have been developed for MPV reduction, such as Sn‐ and Zr‐containing Lewis acid zeolites.

Corma et al. demonstrated that stannosilicate Sn‐Beta with stronger stannum‐derived Lewis acidity exhibited promising catalytic performance in the MPV reaction of cyclohexanone in comparison to the tradition titanosilicates.^[^ ^159^ ^]^ Subsequently, this group also investigated the reaction mechanism that was described according to the following steps, I) adsorption of cyclohexanone and alcohol onto tetrahedrally coordinated Sn sites, II) deprotonation of alcohol, III) hydride transfer through six‐membered ring transition state, IV) proton transfer from the zeolite to cyclohexanone (Figure 15C).^[^ ^161^, ^162^ ^]^ The catalytic activity was related to the reaction substrates. Alkyl‐substituted cyclohexanone such as 4‐*tert*‐butylcyclohexanone and 4‐methylcyclohexanone can be transformed easily over Sn‐Beta zeolite, while the activity of 2‐methylcyclohexanone was much lower and 2‐*tert*‐butylcyclohexanone did not show any conversion. This phenomenon likely resulted from the steric hindrance of the carbonyl group.^[^ ^159^ ^]^ Numerous carbonyl compounds including 4‐methoxybenzaldehyde,^[^ ^163^ ^]^ nopinone, norcamphor, acetophenone, cinnamaldehyde, ^[^ ^130^, ^161^ ^]^ and biomass‐derived 5‐hydroxymethylfurfural (Figure 15D),^[^ ^164^ ^]^ have been reported as reactants in the MPV reduction over Sn‐ and Zr‐zeolites.

The topology structure and physicochemical properties of the silica host also affect the catalytic activity of MPV reduction. Among the stannosilicates, Sn‐Beta exhibited excellent performance even using bulky 4‐*tert*‐butylcyclohexanone molecules as a reactant,^[^ ^159^, ^165^, ^166^ ^]^ indicating that diffusion limitations were not present within microporous zeolites. However, the significant diffusing limitation on the large dimension substrate such as cyclododecanone was observed. Hence, the synthesis of hierarchical Sn‐Beta zeolite with micropore and mesopore structure has become a research hotspot. As for cyclohexanone, microporous and hierarchical Sn‐Beta demonstrated similar reaction rates, while cyclooctanone and cyclododecanone were converted rapidly over hierarchical Sn‐Beta zeolite.^[^ ^143c^ ^]^

The nature of Sn sites would impact the catalytic activity of zeolite for the MPV reduction reaction. Extra‐framework SnO_2_ species, the supporter of SiO_2_ as well as supported SnO_2_ species over SiO_2_ were all useless for the above‐mentioned reaction with cyclohexanone substrate.^[^ ^159^, ^166^ ^]^ In consequence, it is speculated that the isolated framework Sn (IV) in tetrahedral coordination is the active site. Furthermore, Sn sites in open form were key to achieving high catalytic activity for MPV reaction as demonstrated by experimental analysis and density functional theory calculation.^[^ ^162^ ^]^ According to the reaction mechanism, the deprotonation of alcohol benefited from the Sn‐OH‐derived Lewis basicity. Lewis et al. claimed the in situ interconversion of open and closed Sn sites under reaction conditions.^[^ ^164^ ^]^ Consequently, the need for ex situ open Sn sites in Sn‐containing zeolites for MPV reduction reaction is always debatable.

Several catalysts including Zr‐Beta,^[^ ^54^, ^81^, ^111^, ^167^ ^]^ Zr‐Y,^[^ ^128b^ ^]^ Hf‐Beta,^[^ ^168^ ^]^ Hf‐Y^[^ ^169^ ^]^ zeolites and silica‐grafted Sn^[^ ^170^ ^]^ have also been studied as catalysts for MPV reduction besides Sn‐Beta zeolite. Among them, stannosilicate Sn‐Beta and zirconosilicate Zr‐Beta become the most common Lewis acidic nanoporous materials. The combination of the Hf active site with FAU‐type topology structure should be paid attention to in the future. The partial catalytic applications of Sn/Zr‐zeolites as redox catalysis are shown in **Table** **2**.

**Table 2:** The catalytic applications of Sn/Zr‐zeolites as redox catalysis.

Catalyst	Substrate	Desired product	Solvent	T [°C]	Time [h]	Conv. [%]	Sel. [%]	References
Sn‐MFI	2‐adamantanone	ketone	dioxane	95	16	92.0	>98.0	[17b]
Sn‐Beta	2‐adamantanone	ketone	chlorobenzene	90	8	>99.0	>99.0	[38]
Sn‐Beta	cyclohexanone	lactone	dioxane	90	3	53.0	>52.0	[40]
Sn‐Beta	2‐adamantanone	lactone	MTBE	56	6	96.0	>94.0	[40]
Sn‐Beta	2‐adamantanone	lactone	chlorobenzene	90	5	90.0	>99.0	[77]
Sn‐Beta	cyclohexanone	lactone	fluorobenzen	85	0.67	19.2	91.2	[77]
Sn‐Beta	2‐adamantanone	lactone	chlorobenzene	90	4	95.6	>99.0	[79a]
Sn‐UTL	cyclohexanone	cyclohexanol	2‐propanol	100	8	30.7	97.2	[92]
Sn‐UTL	cyclohexanone	lactone	acetonitrile	75	3	23.8	72.7	[92]
Sn‐UTL	2‐adamantanone	lactone	chlorobenzene	90	6	>99.0	>99.0	[92]
Sn‐*CTH	2‐adamantanone	lactone	chlorobenzene	90	5	89.7	>99.0	[94]
Sn‐*CTH	cyclohexanone	lactone	acetonitrile	75	1	20.0	92.4	[94]
Sn‐Y	2‐adamantanone	lactone	chlorobenzene	90	0.67	97.5	97.3	[123a]
Sn‐MSE	2‐admantanone	lactone	chlorobenzene	90	1	78.1	77.3	[124]
Sn‐Y	cyclohexanone	lactone	fluorobenzen	85	0.5	12.3	97.8	[127a]
Sn‐Y	2‐adamantanone	lactone	chlorobenzene	90	8	12.8	99.1	[127a]
Zr‐Y	4‐MP	HA	2‐pentanol	120	4	94	75.2	[128b]
Zr‐DZ‐1	2‐adamantanone	lactone	1,4‐dioxane	75	4	83.2	>95.0	[134]
Sn‐Beta	cyclohexanone	cyclohexanol	2‐butanol	100	4	97.5	92.6	[143c]
Sn‐MWW	2‐adamantanone	lactone	1,4‐dioxane	90	4	49.4	97.6	[153]
Sn‐Beta	cyclohexanone	cyclohexanol	2‐butanol	100	1	95.4	95.4	[159]
Zr‐Beta	2‐adamantanone	lactone	1,4‐dioxane	90	7	76.8	>97.0	[162]
Zr‐Beta	cyclohexanone	lactone	2‐butanol	100	1	65.6	86.8	[162]
Zr‐Beta	crotonaldehyde	1‐Butanol	/	200	1	15.5	12.4	[167a]
Zr‐Beta	cyclohexanone	cyclohexanol	2‐propanol	82	5	44.0	99.0	[167b]
Zr‐Beta	cyclohexanone	cyclohexanol	dodecane	80	3	94.2	>99.0	[167d]

4‐MP: 4‐methoxypropiophenone; HA: hydrophobic anethole.

### 3.2 Lewis Acid Catalysis

#### 3.2.1 Ring Opening of Epoxides

The ring opening of epoxides is an important conversion for synthesizing fine chemicals in the chemistry industry. The nucleophilic reagent can easily attack the C─O bond in epoxides that are significant electrophilic reagents, which lead to the ring opening of substrates. In general, oxygen‐ and nitrogen‐containing molecules including water, alcohol, ammonia as well as amine are the nucleophilic reagents.^[^ ^171^ ^]^

**Figure 16:**
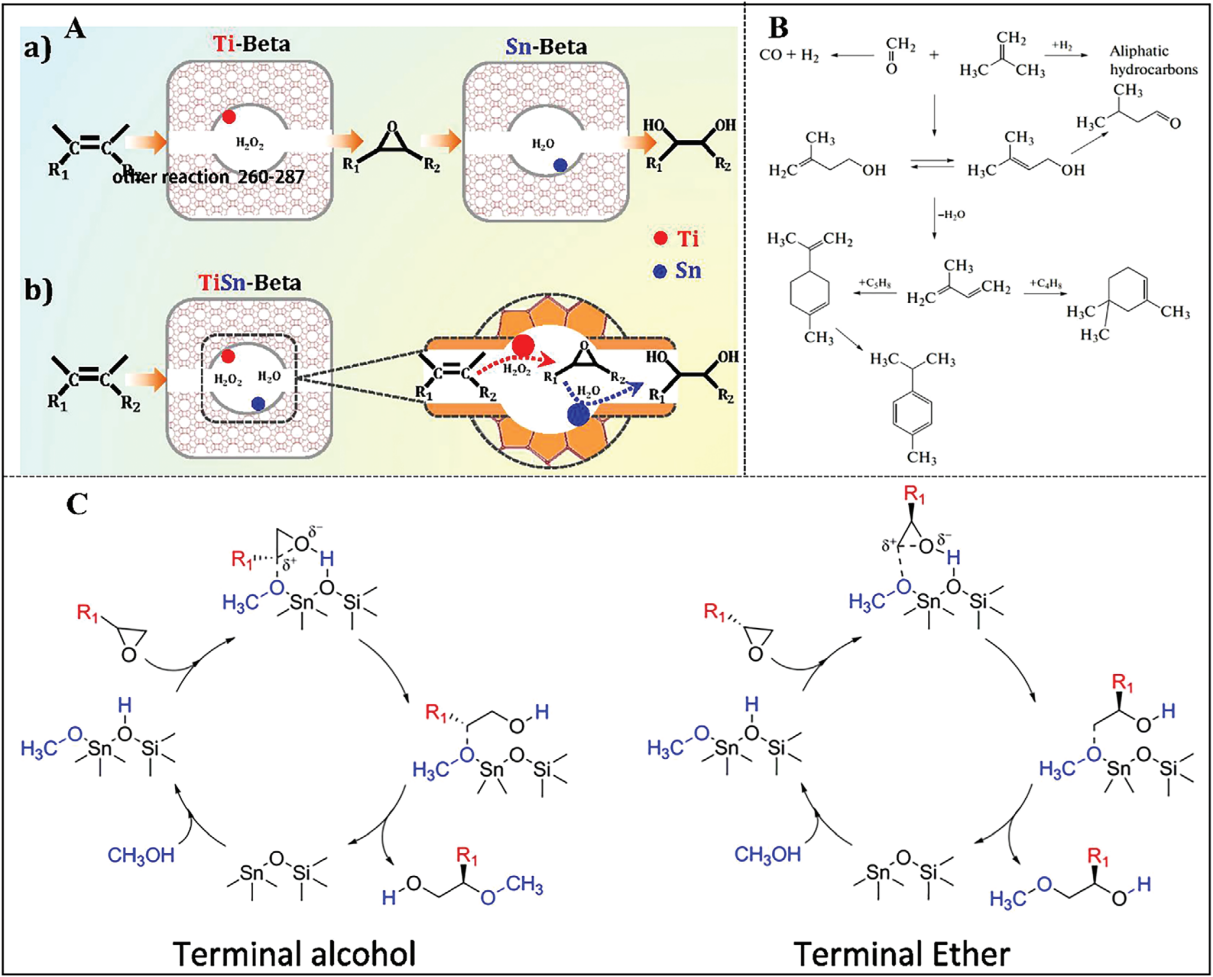
A) Routes for the conversion of alkenes to 1,2‐diols. Route a: indirect route including two processes: alkene epoxidation and epoxide hydration. Route b: tandem catalytic route. Reproduced with permission.^[^ ^174^ ^]^ Copyright 2021, Elsevier. B) Schematic catalytic conversions in the reaction of isobutylene with formaldehyde. Reproduced with permission.^[^ ^184^ ^]^ Copyright 2019, Pleiades. C) Activation of the epoxide by an alcohol that is dissociated and bound to the Sn site. Reproduced with permission.^[^ ^176^ ^]^ Copyright 2018, Elsevier.

Ethylene glycol (EG) is an important bulk chemical intermediate that is applied as solvents, automotive antifreeze, raw material for polyesters, and other down‐stream products.^[^ ^172^ ^]^ At present, EG is produced by the non‐catalytic hydration process of ethylene oxide (EO).^[^ ^172^ ^]^ Sn‐SSZ‐13 (CHA topology) post‐synthesized by SSIE strategy with (CH_3_)_2_SnCl_2_ as Sn source was employed to catalyze EO hydration.^[^ ^173^ ^]^ The narrow 8‐MR pore channels allowed the free diffusion of EO, H_2_O, and EG molecules, but limited the further self‐condensation of EG to byproducts of diethylene glycol (DEG) and triethylene glycol (TEG). The robust Sn‐derived Lewis acidity confined in 8‐MR SSZ‐13 structure has made a remarkable breakthrough in the formation of EG under mild conditions with a near stoichiometric H_2_O/EO molar ratio. DFT calculation demonstrated Sn active sites greatly decreased the activation energy of the reaction process and also further confirmed the open Sn(OSi)(OH)_3_ configuration was the most favorable active site.^[^ ^173^ ^]^ Later, Li et al. designed TiSn‐Beta bifunctional Lewis catalyst again, which converted alkene to produce 1,2‐diols (**Figure** **16**A).^[^ ^174^ ^]^ The isolated Ti and Sn sites facilitated the alkene epoxidation and epoxide hydration, respectively. Recently, Liu group synthesized Sn‐PCR with Sn active site confined in the 10 × 8‐MR channels, which served as a promising shape‐selective catalyst for EO hydration, reaching a remarkable catalytic performance of 99.5% EO conversion and a steady 98.4% EG selectivity under extremely mild conditions.^[^ ^93^ ^]^

The products prepared through ring opening of epoxides with alcohol are widely applied in the solvents and pharmaceutics industry.^[^ ^175^ ^]^ Solid Lewis acid catalysts M‐Beta (M = Sn, Zr, Hf) were prepared for the regioselective ring opening of epichlorohydrin with methanol (Figure 16C).^[^ ^176^ ^]^ The excellent catalytic performance mainly benefited from the types of framework structure and Lewis acid sites. Sn‐Beta zeolite synthesized with the assistance of fluoride exhibited more efficient performance for substrate conversion and product regioselectivity in comparison to other metallosilicates. Both experimental data as well as theoretical DFT calculation verified the reaction mechanism. Alcohol adsorbed on the Sn sites first activated epichlorohydrin. Another alcohol molecular then further nucleophilically attacks the substrate. Besides, the Beta topology structure was well preserved without the evident decrease in conversion after the fourth run.^[^ ^176^ ^]^

Titanosilicate catalysts were also capable of triggering epoxides aminolysis without solvent under mild conditions.^[^ ^177^ ^]^ Inspired by the research, Ti/Zr‐containing MFI zeolites were then synthesized with the purpose of catalyzing the ring opening of epoxides with alcohol or amine.^[^ ^178^ ^]^ It was proved that the framework Zr species endowed Zr‐MFI with catalytic properties. Recycling experiments confirm that no significant change in the activity was observed, even after five recycles. ICP analysis confirmed that Zr was not leached during the reaction. Textural characterizations using XRD and surface area analysis confirmed that the catalyst is stable.^[^ ^178^ ^]^ Subsequently, hierarchical M‐Beta (M = Sn, Zr, Ti) zeolites were postsynthesized by the combination of sequential acid‐alkaline treatment and SSIE method, which were also employed to the ring opening of styrene oxide with aniline.^[^ ^143a^ ^]^ With moderate zirconium‐derived Lewis acidity, mesoporous Zr‐Beta zeolite possessed a higher styrene oxide/aniline adsorption ratio. As a result, this catalyst gave better catalytic performance in styrene oxide aminolysis in comparison with hierarchical Sn‐ and Ti‐Beta zeolites. The presence of mesopores favored the diffusion of molecules and improved the accessibility of active sites, resulting in enhanced catalytic activity.^[^ ^143a^ ^]^ Under mild conditions, zirconosilicate Zr‐MOR also performed well in activity and regioselectivity in epoxides aminolysis reactions.^[^ ^179^ ^]^ The catalytic results in the ring opening of epoxide with amines were determined by the intrinsic nature of substrates (e.g., basicity) and active sites (e.g., Lewis acidity).

#### 3.2.2 Aldol Reaction

**Figure 17:**
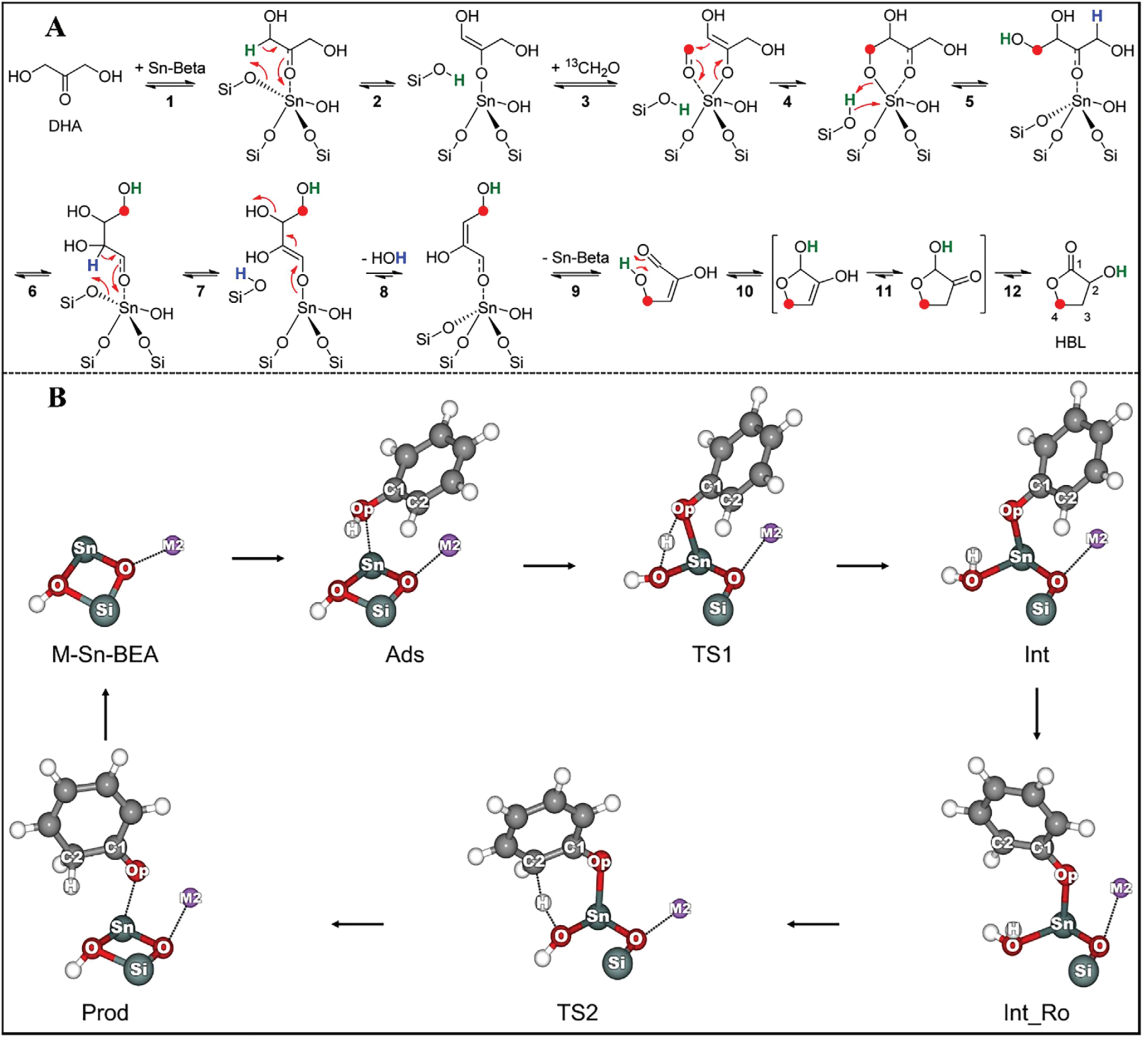
A) Proposed mechanistic pathway for the C─C coupling between DHA and formaldehyde catalyzed by the framework Lewis acidic Sn sites in Sn‐Beta. Reproduced with permission.^[^ ^181^ ^]^ Copyright 2015, American Chemical Society. B) Reaction mechanism scheme for phenol tautomerization on M‐Sn‐BEA (M = Li, Na, K, Rb, and Cs). Reproduced with permission.^[^ ^191^ ^]^ Copyright 2021, Elsevier.

As representative solid Lewis acid catalysts, stannosilicates are able to catalyze aldol reactions to contribute new C─C bonds. Among them, Sn‐Beta zeolite has been reported to transform glycolaldehyde into lactates via the aldol reaction route.^[^ ^180^ ^]^ Subsequently, the coupling of dihydroxyacetone (DHA) and formaldehyde over various M‐zeolite (M = Ti, Sn, Zr, Hf, Al, Sn) catalysts generated *α*‐hydroxy‐*γ*‐butyrolactone (HBL).^[^ ^181^ ^]^ Compared with other zeolites (<11%), stannosilicates Sn‐MFI, Sn‐Beta catalysts showed much higher HBL yield of 64%, 61%, and 60%, respectively, with comparable DHA conversion. In addition, the research also confirmed that SnO_2_ was inactive for this reaction, indicating that the HBL selectivity mainly depended on the coordination state of metal species rather than the zeolite framework structure. The reaction mechanism was revealed from spectroscopic analysis, theory calculation, and isotopic labeling, I) soft enolization of DHA, II) aldol addition of formaldehyde to the Sn‐enolate, III) forming erythrulose (**Figure** **17**A).^[^ ^181^ ^]^

Numerous Sn‐containing zeolites (Sn‐Beta, Sn‐MFI) were adopted for the selective transformation from glycolaldehyde to tetrose sugars under mild conditions (<100 °C, water).^[^ ^44a^ ^]^ Relatively high selectivity (97%) and yield (74%) of C_4_ sugars were observed for the Sn‐MFI catalyst. For a control experiment, other zeolites obtained lower C_4_ sugar selectivity due to the formation of hexose sugars via consecutive aldol condensation of glycolaldehyde.^[^ ^44a^ ^]^ Lewis et al. proceeded with an aldol reaction of aromatic aldehydes with acetone over M‐Beta (Hf, Zr, Sn) zeolites.^[^ ^144^ ^]^ Acid‐base pairs in the Si‐O‐metal groups favored soft enolization through *α*‐proton abstraction. Those Lewis acid catalysts can resist the adverse influence of acidic solution and water on catalytic activity, which is different from the usual base catalysts. Unlike the DHA and formaldehyde substrates, herein, the Zr‐Beta catalyst exhibited superior activity than other zeolites (Sn‐ and Hf‐Beta).^[^ ^181^ ^]^

Whereas, Sn‐MFI is less active than Sn‐Beta zeolite in the synthesis of isopulegol, which was attributed to not only the extremely small pore diameter but also the low molar ratio of open to closed Sn sites for tetrahedrally coordinated Sn species.^[^ ^182^ ^]^ Similar to MPV reduction and B‐V oxidation, open Sn sites were more active than closed Sn sites for carbonyl‐ene reaction.

Apart from stannosilicate zeolites that are capable of catalyzing the carbonyl‐ene reaction, Al‐ and Zr‐containing zeolites can also accomplish this goal.^[^ ^183^, ^184^ ^]^ With respect to the produce of isoprene (a monomer for the large‐scale synthesis of isoprene rubber) from isobutylene and formaldehyde, targeted product yield was correlated with Brønsted acid amounts and topology structure through screening the zeolite catalysts (Al‐MFI, Al‐Beta, Al‐FAU, Zr‐Beta, Sn‐Beta and Nb‐Beta) (Figure 16B).^[^ ^184^ ^]^ It was shown that high Brønsted acid amounts and medium‐pore MFI zeolites were advantageous for the formation of isoprene. Mesoporous 3D Zr‐TUD‐1 material demonstrated high conversion and selectivity of above 99% in the cyclization of citronellal to isopulegol, where no diffusion limitation was noticed.^[^ ^183^ ^]^ Good stability was also observed in the recycling experiments even using industrial‐grade citronellal.^[^ ^183^ ^]^

#### 3.2.3 Propane Dehydrogenation

Light olefins are important bulk chemical feedstock in the petrochemical industry with great demand. At present, the catalytic cracking or methanol‐to‐olefins (MTO) processes mainly account for alkenes production.^[^ ^185^ ^]^ An alternative route, dehydrogenation of light alkanes, is also developed to produce light olefins. Extensive catalysts (Pt, Sn, Ga, V, CrO_x_) were used in this reaction.^[^ ^8^, ^186^ ^]^ In this part, stannosilicates are focused on converting propane into propene. In 2016, Wang group demonstrated that metallic Sn, a highly active species, was active in propane dehydrogenation reaction.^[^ ^185^ ^]^ This result made a breakthrough for the traditional concept that Sn only played a promoter role.^[^ ^185^ ^]^ Compared with PtSn‐based catalyst, the catalytic performance of impregnated Sn/SiO_2_ catalyst remained at the same level. ^[^ ^185^ ^]^ In order to protect Sn species from loss, hexagonal mesoporous silica (Sn‐HMS) with Sn species in framework position was synthesized successfully.^[^ ^187^ ^]^ Sn‐HMS with isolated Sn sites confined in the framework structure was able to resist the tin loss and Sn ions reduction, improving the stabilization of the catalyst significantly. Propane was adsorbed on the isolated Sn species connecting with three framework Si‐O groups, forming intermediates by coordination of Sn^δ+^ to isopropyl group and of oxygen in Si‐O‐Sn group to *α*‐H in propane. Finally, propene was produced via *β*‐H cleavage.^[^ ^187^ ^]^ Subsequently, the same group reported that the isolated Sn^2+^ ions anchoring on mesoporous silica showed excellent activity and stability for propane dehydrogenation due to the strong interaction between Sn^2+^ ions and the support.^[^ ^188^ ^]^

Yue et al. found that the post‐treated Sn‐Beta with framework Lewis acidity can efficiently activate C─H bond in propane, which exhibited excellent catalytic performance in dehydrogenation reaction.^[^ ^189^ ^]^ Meanwhile, Brønsted acidity can catalyze cracking reactions. As a result, a high ratio of Lewis/Brønsted acid benefited the dehydrogenation reaction. When the calcined Sn‐Beta was exchanged with Na^+^ to cover Brønsted acid site, giving rise to the outstanding propane conversion (40%) and propene selectivity (92%). Moreover, the deactivation phenomenon was nearly not observed after 72 h. For the dehydrogenation reaction, open Sn sites in the framework were presumed to be more efficient than closed Sn sites.^[^ ^189^ ^]^

#### 3.2.4 Other Reactions

Sn/Zr‐zeolites can also be extensively applied to other reactions as robust Lewis acid catalysts. Heteroatom‐containing (Ti, Zr, Sn, and Ce) Beta zeolites were post‐synthesized and exploited into the ketonization of propionic acid in the vapor phase.^[^ ^190^ ^]^ Zr‐Beta zeolite presented optimal catalytic performance in terms of activity and selectivity compared with other zeolites. The linear correlation between ketonization activity and Lewis acid density indicated that these Lewis acid sites were responsible for this ketonization reaction, which were derived from isolated framework Zr species mainly in the form of open sites. The stability test of the Zr‐Beta catalyst was performed at 350 °C with *W*/*F* = 2. The conversion maintains at ≈50% over 60 h on the stream, with the 3‐pentanone selectivity higher than 96%. The excellent stability of Zr‐Beta with a high selectivity toward 3‐pentanone is a result of its low activity for further aldol condensation of 3‐pentanone.^[^ ^190^ ^]^ In addition, thermogravimetric analysis also demonstrated the stability of Zr‐Beta zeolites remarkably outperformed that of H‐Beta zeolites due to the less carbon deposition.^[^ ^190^ ^]^

Density functional theory calculation predicted that Zr‐Beta and Sn‐Beta can catalyze phenol tautomerization and the catalytic performance was enhanced to some extent with the exchange of alkali metal ions (Figure 17B).^[^ ^191^ ^]^ After further analysis, it was found that this reaction contained two steps, the dissociation of O‐H in phenol and the subsequent protonation of the intermediate, where the first one was the rate‐determining step.^[^ ^192^ ^]^

*N*‐alkylation reaction of aniline with benzyl alcohol was attempted over various silicates (MFI and Beta) with Sn, Ti, Zr, and Hf active sites.^[^ ^22^ ^]^ Among these zeolites, Zr‐Beta and Hf‐Beta exhibited excellent activity and selectivity for this conversion. The induction period can be suppressed through a pre‐activation with the reactants such as benzyl alcohol. This reaction was carried out by borrowing hydrogen from the substrate of benzyl alcohol and then forming an Hf‐hydride intermediate.^[^ ^22^ ^]^

Self‐condensation of butanal to 2‐ethyl‐2‐hexenal (2‐EN) is a significant step in the produce of 2‐ethylhexanol (2‐ethyl‐1‐hexanol) (an important industrial alcohol).^[^ ^193^ ^]^ Zr‐Beta was confirmed to be an active and selective catalyst for this reaction.^[^ ^55d^ ^]^ The byproducts were mainly 1‐butanol, butanoic acid, 4‐heptanone, butyl butanoate, and octadienes produced by different reaction routes such as Cannizzaro disproportionation, ketonization, esterification, and tandem hydrogenation/dehydration. The conversion of open Zr sites was superior to that of closed Zr sites, while both sites possessed similar 2‐EN selectivity. Thus, Zr‐Beta with enhanced open Zr sites is the desired catalyst for the selective synthesis of 2‐EN.^[^ ^55d^ ^]^ The partial catalytic applications of Sn/Zr‐zeolites as Lewis acid catalysis are demonstrated in **Table** **3**.

**Table 3:** The catalytic applications of Sn/Zr‐zeolites as Lewis acid catalysis.

Catalyst	Substrate	Desired product	Solvent	T [°C]	Time [h]	Conv. [%]	Sel. [%]	References
Sn‐MFI	glycolaldehyde	C4 sugars	water	80	0.5	76.0	74.0	[44a]
Zr‐Beta	butanal	2‐EN	/	260	3	57.5	46.1	[56d]
Sn‐PCR	EO	EG	water	40	10	99.5	98.4	[93]
Zr‐Beta	styrene oxide	amino alcohols	solvent‐free	35	0.5	80.7	76.4	[143a]
Zr‐Beta	furfural	γ‐valerolactone	2‐propanol	120	24	>99.0	95.0	[143a]
Zr‐Beta	benzaldehyde	benzalacetone	toluene	90	5	94.0	92.1	[144]
Sn‐Beta	cyclohexene oxide	cyclohexanediol	water	40	6	90.2	92.7	[171]
Sn,Ti‐MWW	ethylene	EG	water	40	5	>99.0	96.2	[172]
Sn‐CHA	epoxypropane	1,2‐propanediol	water	60	8	72.5	71.1	[173]
Sn,Ti‐Beta	epoxypropane	1,2‐propanediol	water	60	2	74.6	93.4	[174]
Zr‐ZSM‐5	styrene oxide	β‐amino alcohol	amine	50	0.75	89.0	86.0	[178]
Zr‐MOR	styrene oxide	β‐amino alcohol	amine	60	4	100	91.0	[179]
Sn‐Beta	fructose	methyl lactate	water	160	16	>99	54.0	[180]
Sn‐Beta	lyxose	methyl lactate	methanol	160	16	96	39	[180]
Sn‐Beta	mannose	methyl lactate	methanol	160	16	96	47	[180]
Sn‐Beta	turanose	methyl lactate	methanol	160	44	72	26	[180]
Sn‐Beta	DHA	HBL	dioxane	160	3	98.0	68.0	[181]
Sn‐Beta	DHA	BHB	dioxane	160	3	98	60	[181]
Zr‐TUD‐1	citronellal	isopulegol	toluene	80	0.5	99.5	99.0	[183]
Sn‐Beta	propane	propylene	/	630	72	40.0	92.0	[189]
Zr‐Beta	propionic acid	3‐pentanone	/	350	0.5	33.1	93.9	[190]

2‐EN: 2‐ethyl‐2‐hexenal; EO: ethylene oxide; EG: ethylene glycol; DHA: dihydroxyacetone; HBL: α‐hydroxy‐γ‐butyrolactone;

BHB: α,β‐butenolide α‐hydroxy‐γ‐butyrolactone.

### 3.3 Biomass Catalysis

Isomorphous incorporation of tetrahedral Sn or Zr active centers into zeolite framework generated rich Lewis acid sites, which can interact with electron‐rich substituents (e.g. carbonyl groups, alcohol groups) and then activated reaction substrates.^[^ ^194^ ^]^ Considering the presence of plentiful carbonyl and alcohol groups in bio‐molecules, Sn/Zr‐zeolites are expected to serve as a promising catalyst in the field of biomass catalysis.^[^ ^195^ ^]^ In this regard, biomass as a clean energy source bears the potential ability to further convert into high‐value fuels and other down‐stream products.

#### 3.3.1 Sugar Isomerization

The report of monosaccharides isomerization upon Lewis acid catalysts is later in comparison to the reaction over basic catalysts. Generally, the representative Lewis acid catalysts (e.g., AlCl_3_) are preferentially coordinated with water molecules and then encounter partial hydrolysis, resulting in the easy inactivation of catalysts.^[^ ^196^ ^]^ Therefore, those reactions using Lewis acid catalysts usually proceed under anhydrous conditions. Moreover, the catalyst/H_2_O system, featured by the greenness with water as the sole solvent, is consistent with the concept of sustainable development and has already attracted significant attention.^[^ ^3^, ^5^, ^197^ ^]^ In consequence, the carbohydrate isomerization over Lewis acid catalysts with water as the sole solvent has aroused great research interest.

Moliner et al. studied the catalytic property of glucose isomerization reaction over metallosilicates (Sn‐Beta and TS‐1) under the aqueous condition in 2010.^[^ ^198^ ^]^ As a representative catalyst, Sn‐Beta with high hydrophobicity showed superior activity and fructose selectivity. The reaction substrate can be completely converted when the concentration of glucose solutions is even up to 45 wt.%. This finding inaugurates a new avenue for biomass conversion with heterogeneous Lewis acid Sn‐Beta catalysts. The Sn‐Beta zeolite is also capable of catalyzing other isomerization reactions with different biomass substrates such as lactose,^[^ ^114b^ ^]^ xylose,^[^ ^125^, ^199^ ^]^ lyxose,^[^ ^180^ ^]^ mannose,^[^ ^180^ ^]^ and galactose.^[^ ^180^ ^]^

For the isomerization of xylose and dihydroxyacetone (DHA), Lari et al. studied the influence of zeolite framework structure, synthesis method, solvent type, and hydrophobicity on the catalytic performance of Sn‐zeolites.^[^ ^200^ ^]^ For the transformation of xylose to xylulose in batch mode in water, Sn‐Beta‐F was more active than other Sn‐zeolites. Once changing the operation from batch mode to continuous mode within 24 h, alkaline‐aided Sn‐MFI was the most stable catalyst, whose loss percent to initial activity was minimum (50%) compared with Sn‐MFI‐F (80%), Sn‐Beta‐F (80%), Sn‐MOR (90%), Sn‐Beta (90%), and Sn‐FAU (99%). In the aqueous solution, the decrease in activity and selectivity was mainly attributed to framework amorphization, Sn site restructuring, and Sn leaching. In CH_3_OH system, these effects were mitigated.^[^ ^200^ ^]^ Similar work was conducted for glucose isomerization over Sn‐substituted BEA, MOR, MFI, and MWW zeolites.^[^ ^201^ ^]^ It was evidenced that Sn‐Beta and Sn‐MOR with 12‐MR pores can fuel glucose isomerization effectively and 10‐MR Sn‐MFI and Sn‐MWW hardly catalyzed glucose because of the narrow pores.^[^ ^201^ ^]^ Surprisingly, Sn‐MFI with nanosheet structure performed excellent glucose conversion (31.4%) and fructose selectivity (87.1%) than Ge and Zr‐substituted MFI nanosheets.^[^ ^20^ ^]^

Sn‐Beta zeolite prepared by SSIE exhibited a 90% loss of initial activity for glucose isomerization in water in continuous flow after 30 h. Whereas, the stability was improved significantly in CH_3_OH solution.^[^ ^202^ ^]^ The deactivation mechanism also gave insight into CH_3_OH and a mixture solvent containing CH_3_OH:H_2_O of 9:1. The presence of water was beneficial for the decrease of carbonaceous residues within pores and the changes in Sn micro‐environment. van der Graaff et al. compared the catalytic activity and investigated the deactivated mechanism for glucose isomerization over hydrothermally synthesized Sn‐Beta‐F and post‐synthesized Sn‐Beta‐PS.^[^ ^203^ ^]^ Sn‐Beta‐F maintained 30% of initial activity after 24 h in continuous mode. The deactivated Sn‐Beta‐F cannot recover to the initial activity through the calcination method at elevated temperatures. The deactivation was caused by carbon deposition and framework damage. Sn‐Beta‐PS zeolite possessed worse activity and stability compared to Sn‐Beta‐F, whose deactivation was ascribed to framework damage and Sn leaching. The stability can be enhanced for Sn‐Beta‐F zeolite by using a mixture solvent of ethanol and water.^[^ ^203^ ^]^

The structure configuration of Sn sites in the Sn‐Beta framework affected significantly the catalytic behavior of the isomerization reaction. It was generally accepted that the open Sn sites are responsible for the activity regarding MPV reduction and B–V oxidation.^[^ ^2a^ ^]^ As for the isomerization reaction, theory calculation predicted that the open sites of Sn were more active than the closed Sn sites used.^[^ ^41^, ^204^ ^]^ In the hydride transfer step, the reaction transition state was stabilized by both open Sn sites and adjacent silanol groups. The open‐chain glucose linked with Sn in a monodentate form, while the ─OH group in glucose coordinated with the silanol group by hydrogen bonding. The above‐mentioned coordination mode contributed to hydride transfer and the following proton transfer step, reducing the activation energy for this reaction. Vlachos et al. also calculated the epimerization of glucose into mannose and predicted this process proceeding via the Bilik mechanism.^[^ ^204^ ^]^ Soon afterward, Bermejo‐Deval et al. excluded the participation of silanol groups in the epimerization of glucose into mannose through Na^+^ ions exchange with silanol groups.^[^ ^205^ ^]^ Ammonia‐modified Sn‐Beta zeolite displayed a sharp decrease in activity due to the poisoning of open Sn sites by NH_3_.^[^ ^205^ ^]^

The isomerization mechanism has been broadly investigated by spectroscopic and computational techniques. NMR and IR spectra indicated that Sn‐Beta zeolite adsorbed glucose and fructose molecules in open‐chain forms.^[^ ^206^ ^]^ It was well‐established that the glucose isomerization obeyed an intramolecular hydride shift from the C2 to C1 position in glucose.^[^ ^207^ ^]^ The detailed mechanism of glucose isomerization over Sn‐Beta zeolite was narrated as follows, I) adsorption and activation of glucose via its coordination to active sites; II) hydride transfer; III) desorption of fructose. The key step of hydride transfer was kinetically relevant (rate‐limiting step) through kinetic isotopic investigations.^[^ ^41^, ^207^, ^208^ ^]^ Hammond et al. investigated the continuous flow activity of Sn‐Beta for the isomerization of glucose to fructose. Extended time‐on‐stream studies reveal Sn‐Beta to be a very stable catalyst during continuous operation in an organic solvent. Spectroscopic methodologies reveal that deactivation in these cases is related to fouling of the micropores with the product and higher molecular weight carbonaceous residue. Periodic regeneration by heat treatment is found to restore full activity, allowing Sn‐Beta to be used for over 700 h continuously with no greater than 20% loss in activity. In contrast, operation in an aqueous media is extremely disadvantageous, as it causes total destruction of the catalyst and permanent deactivation. In these cases, however, long‐term activity can still be achieved by modifying the solvent chosen for the reaction, with methanol appearing to be a suitable alternative.^[^ ^20^ ^2]^

#### 3.3.2 5‐(Hydroxymethyl)furfural (HMF) Synthesis

As a potential platform molecule, HMF is capable of being converted to numerous biofuels and bulk chemical products via hydrogenation, hydrogenolysis as well as oxidation processes.^[^ ^209^ ^]^ In general, the dehydration of glucose and fructose from cellulosic biomass can produce HMF.^[^ ^210^ ^]^ Fructose directly dehydrates to yield HMF. But the transformation of glucose to HMF needs two steps and the process involves glucose to fructose and fructose to HMF.^[^ ^210^ ^]^ In other words, fructose is an intermediate for glucose to HMF. Hence, the transformation of fructose to HMF is relatively easier than that of glucose to HMF from this perspective. In Section 3.3.1, Lewis acid sites catalyzed the isomerization reaction of glucose to fructose, which probably generated a mannose byproduct via an epimerization reaction.^[^ ^204^ ^]^ While the process of fructose‐to‐HMF was assisted by Brønsted acid site. As a result, the conversion of glucose to HMF reaction is carried out by using bifunctional catalysts with Lewis and Brønsted acid sites.^[^ ^210^ ^]^ Other byproducts are hardly detected including oligomers (designed as humins) except main products HMF, intermediate fructose, and byproduct mannose during the glucose‐to‐HMF reaction,^[^ ^211^ ^]^ which results in a low mass balance. In addition, several byproducts such as lactic acid, furfural, and levulinic acid are also observed occasionally. The present review focuses on the catalytic applications of Sn/Zr‐zeolites, especially the dehydration of glucose to HMF. Sn/Zr‐zeolites with bifunctional Lewis and Brønsted acid sites are expected to serve as promising catalysts for this reaction. Isolated framework Al ions provide Brønsted acid sites, while the Sn or Zr heteroatoms creat Lewis acid sites. Therefore, developing novel metallosilicates (herein Al, Sn‐containing zeolite) with various framework structures is important for modulating the catalytic performance of glucose‐to‐HMF reaction.

At the very beginning, Lewis acid zeolites were mixed with a homogeneous Brønsted acid solution, triggering the glucose‐to‐HMF reaction. A classic Sn‐Beta catalyst is proven to be highly effective in glucose‐to‐fructose reactions under an aqueous solution with an extremely low pH of 1.0.^[^ ^198^, ^207^ ^]^ This study provides a new idea for the one‐pot synthesis of HMF from glucose under acidic media. Nikolla group employed Sn‐Beta zeolite coupling with acid catalyst of HCl to convert glucose substrate into HMF product through single or biphasic reaction systems.^[^ ^212^ ^]^ The single‐phase system contained a reaction substrate of glucose, Sn‐Beta, and HCl in water, whereas the biphasic system was comprised of glucose, Sn‐Beta, HCl, and organic phases such as 1‐butanol. The use of a biphasic system possessed a higher glucose conversion and HMF selectivity than that of a single system. This result was ascribed to the fact that the produced HMF was extracted from the aqueous phase to the organic phase, therefore inhibiting the transformation of HMF to undesired byproducts. It also confirmed that both Sn‐Beta and HCl were needed for those conversions, where Sn‐Beta and HCl were responsible for glucose‐to‐fructose and fructose‐to‐HMF reactions, respectively.^[^ ^212^ ^]^ Combining with a homogeneous HCl catalyst, a heterogeneous catalyst Al‐Beta zeolite with a Si/Al molar ratio of 12.5 was also applied to proceed with this one‐pot reaction.^[^ ^213^ ^]^ However, it is urgent to develop bifunctional catalysts with both Lewis and Brønsted acid sites in one heterogeneous zeolitic framework for the one‐pot reaction.

In 2015, bifunctional Sn‐Al‐Beta zeolite was prepared by partial dealumination of Al‐Beta zeolite and further CVD treatment.^[^ ^122^ ^]^ The Al and Sn amounts can be modulated by adjusting the dealuminated degree and the contact time with SnCl_4_ vapor, which were associated with Brønsted and Lewis acid sites, respectively. Sn‐Al‐Beta zeolite exhibited 60% of glucose conversion and 62.1% of HMF selectivity under optimal reaction conditions.^[^ ^122^ ^]^ In 2020, hierarchical Sn‐Al‐Beta zeolite was also hydrothermally synthesized with a cyclic quaternary ammonium salt, which was also capable of driving the conversion of glucose to HMF in aqueous system.^[^ ^214^ ^]^

The post‐synthesized method was also utilized to construct hierarchical Sn‐Beta with the purpose of enhancing diffusion performance and decreasing the probability of the formation of humin.^[^ ^119^ ^]^ Yang et al. employed PDADMA as OSDA to hydrothermally synthesize hierarchical Al‐Beta zeolite.^[^ ^119^ ^]^ Subsequently, Al‐Beta was dealuminated and further stannated by the CVD method, giving Sn‐Beta zeolite. This hierarchical Sn‐Beta demonstrated higher HMF yield than microporous Sn‐Beta zeolite, meanwhile which gave excellent stability.

Other topology structures like FAU were also used to accommodate Sn ions. Sn‐Al‐Y zeolite, with the Si/Sn and Si/Al molar ratio of 65 and 164, respectively, exhibited 17% HMF yield and 36% glucose conversion at 140 °C in the water system.^[^ ^215^ ^]^ Nonetheless, this zeolite catalyst deactivated rapidly on account of the formation of humin and/or the competitive adsorption of water to framework Sn sites. When using DMSO as the solvent, good reusability was observed over Sn‐Al‐Y zeolite. In this literature, the catalytic behavior of Ga‐Al‐Beta was also investigated.^[^ ^215^ ^]^

The deactivation phenomenon of zeolite catalysts usually occurs in the catalytic process of glucose‐to‐HMF even though they sometimes demonstrated extraordinary HMF yield. The formation of an undetected humin byproduct can block the pore channels within zeolites. Besides, the framework structure occasionally was destroyed to some extent, in particular in water media.^[^ ^216^ ^]^ The isolated framework metal ions such as Sn ions within certain Sn‐Beta zeolites were not stable in aqueous glucose, which can be migrated from the framework to an extra‐framework position, even further leaching into reaction systems under harsh reaction conditions.^[^ ^203^ ^]^ In general, compared with water media, zeolite catalysts are stable in terms of framework structure and isolated metal ions in organic systems.^[^ ^217^ ^]^ In contrast to the abovementioned research, a dramatic enhancement was observed for the stability of post‐synthesized Sn‐Beta in the continuous mode of glucose‐to‐HMF reaction when adding small amounts of water into the methanol/glucose reaction system.^[^ ^218^ ^]^ This improved stability was ascribed to two points. First, the presence of small amounts of water decreased the accumulation of carbonaceous deposition within the zeolite pores. Second, its presence minimized the changes in the coordination sphere and of the microenvironment of Sn and Si in a hydrated state. The second effect dominated the role of tiny amounts of water in this reaction.^[^ ^218^ ^]^ Consequently, the addition of small amounts of water is an ingenious means to balance activity and stability using Sn‐Beta zeolite as a heterogeneous catalyst for glucose‐to‐HMF in organic solvents.

#### 3.3.3 Synthesis of Lactic Acid or Alkyl Lactates

Lactic acid (LA) is a significant raw material in the field of the food industry, which is also used as the building block of biopolymer and platform chemicals.^[^ ^219^ ^]^ At present, the fermentation of sugars to LA still accounts for the major market share.^[^ ^2a^ ^]^ Subsequently, it was found that Lewis acid catalyst can effectively provoke the synthesis of LA or alkyl lactates (AL) from sugars including but not limited to triose, pentose, and hexoses.

As triose sugars, both dihydroxyacetone (DHA) and glyceraldehyde (GLA) are converted into LA and AL products over Lewis acid catalysts with the solvent of water and alcohol, respectively.^[^ ^32^, ^220^ ^]^ The detailed reaction mechanism contained the following steps, I) dehydration of DHA/GLA to form pyruvic aldehyde over weak Brønsted acid sites or Lewis acid sites, II) conversion of pyruvic aldehyde to hydrate or hemiacetal, III) formation of LA/AL according to intramolecular MPV or Cannizzaro reaction over Lewis acid sites. DHA can isomerize reversibly to form GLA over Lewis acid catalyst following the hydride shift mechanism. Therefore, when using DHA or GLA as substrates, comparable LA/AL yields were obtained.^[^ ^219^, ^221^ ^]^

Among various stannosilicates (Sn‐MFI and Sn‐Beta), Sn‐Beta zeolite exhibited the highest catalytic performance for the synthesis of AL from triose sugars in methanol due to the strongest Lewis acidity.^[^ ^95^ ^]^ But Sn‐Beta with 3D 12‐MR channels only exhibited similar catalytic activity to Sn‐MFI catalyst with 10 × 10‐MR channels in water.^[^ ^222^ ^]^ This result indicated that diffusion limitation could be ignored for this reaction. Other parameters also affected the catalytic behavior, for example, Lewis acid strength, material hydrophobicity, and confinement effect. Considering the faster rate‐limiting step over weak Brønsted acid sites than that over Lewis acid sites,^[^ ^223^ ^]^ it was a robust strategy to enhance the catalytic performance by adding weak Brønsted acid sites into catalysts. Other Sn‐zeolite catalysts, Sn‐MWW,^[^ ^33^ ^]^ Sn‐Beta,^[^ ^77^ ^]^ and Sn‐MFI,^[^ ^222^ ^]^ were also adopted into the transformation of trioses to LA/AL.

In addition, the reaction substrates of pentose and hexose can also be converted to LA/AL through the catalysis effect of Sn/Zr‐zeolites. The conversion of pentose to LA/AL involved two key steps retro‐aldol condensation and triose‐to‐LA/AL reactions. Similarly, the transformation of glucose to LA/AL is a cascade reaction according to the following three steps, including isomerization of glucose to fructose, retro‐aldol condensation of fructose to triose, and conversion of triose to LA/AL.^[^ ^180^ ^]^ The first two steps require the aid of Lewis acid sites. As discussed in the above section, the last step needs Lewis acid sites or weak Brønsted acid sites. Whereas, Brønsted acid sites facilitate that pentose/hexose dehydrate to produce furfural/HMF products. Hence, the conversion process of pentose/hexose to LA/AL takes place over Lewis acid sites or the combination of Lewis acid and weak Brønsted acid sites.^[^ ^180^ ^]^

Sn‐Beta synthesized by different methods was tested in the synthesis of AL from glucose. 44% ML yield was obtained over nanosized Sn‐Beta zeolite post‐synthesized by SSIE approach at 160 °C for 10 h.^[^ ^224^ ^]^ The excellent catalytic performance was attributed to the presence of mesoporosity, which bore the advantage of the enhancement in diffusion performance and the accessibility of active sites. Post‐synthesized hierarchical Sn‐Beta zeolite using hierarchical Al‐Beta as the raw material was also exploited as a catalyst to drive the ML produce.^[^ ^143d^ ^]^ The ML yield of 52.5% over hierarchical Sn‐Beta was superior to that of 43.0% over microporous Sn‐Beta zeolite under the same reaction conditions, derived from the promoting effect in the formation of intermediates.^[^ ^143d^ ^]^ A fluoride‐free and low template content synthesis of hierarchical Sn‐Beta zeolite was reported, which was comprised of three steps, dealumination, stannation, and TEAOH treatment.^[^ ^225^ ^]^ The amounts of TEAOH significantly impacted the textural property of resultant Sn‐Beta zeolite. The optimal Sn‐Beta zeolite afforded a 58% ML yield from glucose at 160 °C for 10 h.^[^ ^225^ ^]^

With the purpose of inhibiting the undesirable side reactions and facilitating the main reaction, the modification of Sn‐Beta is necessary on some occasions. For instance, a Lewis acid‐base catalyst Zn‐Sn‐Beta was established, where the introduction of Zn not only increased the Lewis acidity but also induced the generation of Lewis basicity.^[^ ^226^ ^]^ This bifunctional Zn‐Sn‐Beta zeolite contributed to the LA yield of 48% from glucose in water at 190 °C within only 2 h. The high performance was derived from the inhibiting effect of Lewis basicity on the side reactions related to carbohydrate dehydration. Adding alkali ions in the Sn‐Beta zeolite or in the reaction system directly was confirmed to be an effective method to increase the LA yield, achieving 75% ML yield in methanol.^[^ ^227^ ^]^ This strategy was also suitable for Sn‐Beta‐F and post‐synthesized Sn‐Beta materials. This phenomenon was due to the fact that the presence of alkali ions improved the retro‐aldol condensation and the subsequent generation of ML and suppressed the formation of 3‐deoxyglucosone byproduct.^[^ ^227^ ^]^

#### 3.3.4 Furans to Terephthalic Acid or *p*‐Xylene

Petroleum‐based terephthalic acid (TPA) and its derivatives are conventional monomers for the synthesis of polyesters such as polyethylene terephthalate in the polymer industry, which are generally produced from partial oxygenation of petroleum‐derived *p*‐xylene (PX).^[^ ^228^ ^]^ With the increasing exhaustion of fossil resources and environmental pollution concerns, using biobased resources to produce TPA or PX is a highly appealing route from the perspective of green chemistry and sustainable development.

In 2014, Davis's group reported the synthesis of 4‐(hydroxymethyl)benzoic acid (HMBA, intermediate of PTA) and dimethyl terephthalate (DMT) from 5‐(hydroxymethyl)furoic acid (HMFA, a partially oxidized product of HMF) over solid Lewis acid zeolite catalysts.^[^ ^229^ ^]^ Using Sn‐Beta zeolite as a heterogeneous catalyst, HMFA was reacted with ethylene to produce HMBA with 31% selectivity and 61% HMFA conversion via a Diels–Alder (D–A) and dehydration route at 190 °C for 6 h. Once the function group of HMFA was protected by methanol to generate methyl 5‐(methoxymethyl)furan‐2‐carboxylate (MMFC), it was reacted with ethylene to synthesize methyl 4‐(methoxymethyl) benzenecarboxylate (MMBC) with 28% MMFC conversion and 46% MMBC selectivity over Sn‐Beta zeolite for 2 h. As for the Zr‐Beta zeolite catalyst, 81% MMBC selectivity and 26% MMFC conversion were obtained for 6 h. The products of HMBA and MMBC were able to be oxidized to form PTA and DMT, respectively. Other Lewis acid catalysts such as Sn‐MFI, Ti‐Beta, and Brønsted acid catalyst Al‐Beta, exhibited significantly inferior catalytic performance in this D‐A and dehydration reaction. The presence of Brønsted acid sites induced the formation of humins and coke by‐products. Besides, the fully oxidized product of 2,5‐furandicarboxylic (FDCA) from HMF was inactive in this reaction because the intensive electron‐withdrawing effects of the two carboxyl groups led to a remarkable electron‐poor diene.^[^ ^229^ ^]^ Two side products of methyl 4‐formylcyclohexa‐1,3‐diene‐1‐carboxylate and methyl 4‐formylbicyclo[2.2.2]oct‐2‐ene‐1‐carboxylate were identified between the reaction of MMFC and ethylene over Sn‐ and Zr‐Beta zeolite catalysts.^[^ ^230^ ^]^ Madon‐Boudart tests indicated the D‐A dehydration reaction was reaction‐limited and not diffusion‐limited.^[^ ^230^ ^]^

The computational investigation confirmed that the reaction mechanism obeyed the D–A cycloaddition of ethylene and HMFA followed by a dehydration reaction.^[^ ^231^ ^]^ The first step was rate‐limiting and isolated Sn species stabilized the transition state of D–A cycloaddition via electrostatic interaction, starting from the coordination of carbonyl oxygen in HMFA to the Sn sites.^[^ ^231^ ^]^

**Figure 18:**
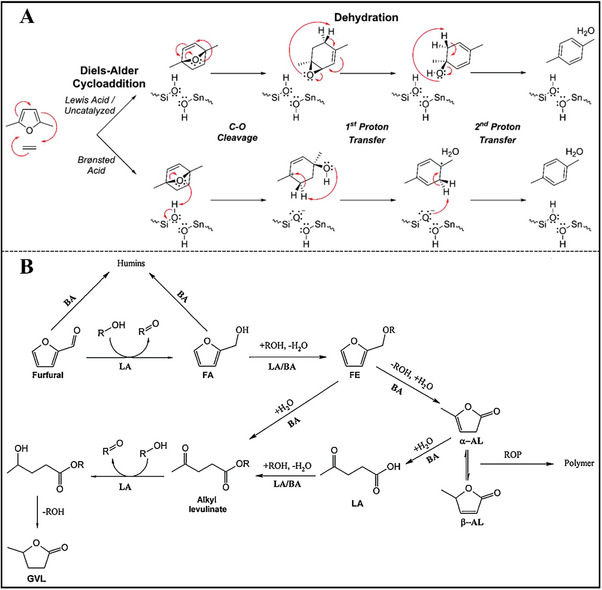
A) Reaction mechanism for the Diels‐Alder cycloaddition of DMF and ethylene, and subsequent three‐step dehydration to water and *p*‐xylene. The top mechanism, uncatalyzed or Lewis acid catalysis; the bottom mechanism, Brønsted acid catalysis. Reproduced with permission.^[^ ^234^ ^]^ Copyright 2017, Wiley‐VCH. B) Reaction networks for the one‐pot conversion of furfural to GVL. Reproduced with permission.^[^ ^237^ ^]^ Copyright 2016, Elsevier.

The conversion of dimethylfuran (DMF) into *p*‐xylene (PX) can be achieved via the D–A dehydration route, which is then able to be oxidized to TPA. Chang et al. compared the catalytic activity of Zr‐, Sn‐, and Ti‐substituted Beta in the synthesis of PX from DMF and ethylene.^[^ ^232^ ^]^ Zr‐Beta zeolite had the highest catalytic performance in terms of turnover frequency, lifetime, and PX selectivity due to its inhibiting effect of DMF to 2,5‐hexanedione and further polymerization. Under optimal conditions, 99% DMF conversion and 90% PX selectivity were afforded over Zr‐Beta zeolite with a Si/Zr molar ratio of 168. Additionally, Zr‐Beta facilitated the D–A cycloaddition reaction, but was less efficient for the subsequent dehydration. As for this tandem reaction consisting of D–A and dehydration reactions, the compromised result was that a moderate improvement was observed in catalytic performance for the Zr‐Beta zeolite catalyst compared with Al‐Beta zeolite.^[^ ^232^ ^]^ Yu et al. also performed this reaction in a continuous reactor over Al‐Beta, Sn‐Beta, and Zr‐Beta zeolites.^[^ ^233^ ^]^ The first order rate was noticed for both DMF and ethylene over these three zeolite catalysts. In addition, Zr‐Beta and Sn‐Beta were stable in the lifetime test, however, Al‐Beta zeolite was easily deactivated, resulting from its rapid oligomerization of DMF and 2,5‐hexanedione. ^[^ ^233^ ^]^ Patet et al. found that Sn‐Beta and Zr‐Beta had comparable catalytic activity, while Ti‐Beta zeolite was inactive.^[^ ^234^ ^]^ The open metal sites were the catalytically active sites and correspondingly, silanol groups of open sites based on Brønsted acid sites were responsible for the hydrolysis of furan ring (**Figure** **18**A).^[^ ^234^ ^]^ Toluene can be also produced from 2‐methylfuran and ethylene with > 90% conversion and > 70% selectivity via D–A cycloaddition and dehydration reactions over Lewis acid catalysts such as Sn‐ and Zr‐Beta zeolites.^[^ ^235^ ^]^

#### 3.3.5 γ‐Valerolactone Synthesis

As an important fine chemical, solvent, and fuel additive, the synthesis process of GVL from furfural or sugars has attracted significant research interests.^[^ ^236^ ^]^ Furfural would experience four successive cascade steps to reach GVL including hydrogenation of furfural, ring opening, hydrogenation of levulinic acid, and cyclization.^[^ ^56a^ ^]^ Both furfural and levulinic acid are hydrogenated over Lewis acid sites, obeying the MPV hydrogen transfer mechanism. While Brønsted acid sites catalyzed the process of ring opening of the furfural ring. Hence, the whole process needs bifunctional catalytic centers (Lewis and Brønsted acid sites). In the beginning, Lewis acid catalyst Zr‐Beta and Brønsted acid catalyst Al‐MFI were coupled to carry out the synthesis of GVL from furfural. Seventy‐eight percent product yield was obtained in a 5 wt.% furfural solution with the hydrogen source and solvent of 2‐butanol.^[^ ^56a^ ^]^ A Physical mixture of Zr‐Y and Al‐Y was also performed for the conversion of furfural to GVL, giving 85% GVL yield at 120 °C for 5 h.^[^ ^128a^ ^]^ In view of the proximity of Lewis and Brønsted acid sites, packaging these two sites into one host is highly desired. Thus, Sn‐Al‐Beta zeolite was post‐synthesized to conduct the produce of GVL from furfural.^[^ ^237^ ^]^ The amounts of Al and Sn were tightly related to the Brønsted and Lewis acidity, respectively, then impacting the catalytic performance. Sn‐Al‐Beta with Si/Al and Si/Sn of 473 and 63, respectively, exhibited an optimal GVL yield of 60% at 180 °C in 2‐butanol. The GVL yield over integrated Sn‐Al‐Beta was higher than a physical mixture of Sn‐Beta and Al‐Beta, indicating the necessity of close proximity for Brønsted and Lewis acid sites within Sn‐Al‐Beta (Figure 18B).^[^ ^237^ ^]^ Analogously, microporous Zr‐Al‐Beta and mesoporous Zr‐Al‐TUD‐1 were prepared to drive the GVL produced from biomass‐derived furfural.^[^ ^238^ ^]^

Hierarchical Meso‐Zr‐Al‐Beta zeolites were developed by multiple‐step post‐synthesis for the furfural‐to‐GVL reaction.^[^ ^143b^ ^]^ The presence of mesopores was good for increasing the accessibility of active sites and improving the diffusion property. This Zr‐zeolite catalyst exhibited the highest GVL yield of 95% and excellent recyclability.^[^ ^143b^ ^]^ Heteropolyacid (HPA) was loaded on Zr‐Beta zeolite to establish an HPA/Zr‐Beta catalyst, where Zr‐Beta and HPA acted as Lewis and Brønsted acid sites, respectively.^[^ ^239^ ^]^ HPA/Zr‐Beta demonstrated an outstanding GVL yield of ≈70% at 160 °C for 24 h, which was higher than that of Sn‐Al‐Beta due to the strong Brønsted acidity and excellent thermal stability.^[^ ^239^ ^]^ Besides, the spent HPA/Zr‐Beta catalyst was subjected to calcination at high temperatures (>573 K) for its regeneration. The catalysis results showed that the initial activity of the catalyst was completely regained after calcination at 773 K, reaching ≈68% GVL yield. Thus, these results suggest that the HPA/Zr‐Beta is reusable after calcination in air.^[^ ^239^ ^]^

The downstream levulinic acid can be also converted to GVL product by MPV reduction over Zr‐Beta catalyst.^[^ ^56b^ ^]^ The GVL yields were up to 96% and 99% in a batch and continuous flow reactor, respectively. When using xylose as the starting reactant, Brønsted acid sites are needed to catalyze the dehydration step of xylose to furfuran that is different from the catalytic process with furfuran as the substrate.^[^ ^131^, ^132^ ^]^ Zr‐Al‐Beta zeolite with Lewis and Brønsted acidity was also applied to proceed with the one‐pot conversion of xylose to GVL. Under optimal reaction conditions (190 °C, catalyst loading of 15 g L^−1^, xylose concentration of 30.5 g L^−1^), 34% GVL yield was observed within 10 h.^[^ ^132^ ^]^ Zr‐Al‐Beta zeolite can be also used to drive the catalytic reaction of glucose‐to‐GVL.^[^ ^240^ ^]^ Al and Zr contents remarkably influenced the product distribution. The GVL yield achieved maximum at the Al/Zr molar ratio of 0.2. Optimizing reaction temperature (190 °C) and time (24 h) led to a maximum GVL yield of 24%.^[^ ^240^ ^]^ From the above catalytic results, we know that using xylose as the starting reactant was more favorable to the GVL formation compared to the reaction substrate of glucose, although both the two catalytic conversions required the assistance of bifunctional zeolite catalyst with Brønsted and Lewis acidity.

#### 3.3.6 Ether Synthesis

Ethers are widely applied in fine chemicals, polyester building blocks, and biofuel additives, which are traditionally produced between two alcohol molecules by acid‐catalyzed (Lewis or Brønsted acid) condensation.^[^ ^2^, ^164^ ^]^ In 2010, Bozell et al. conducted ether synthesis from benzyl alcohol derivatives and butanol over Sn‐Beta stannosilicate.^[^ ^163^ ^]^ Alcohol was usually produced from the oxidation of aldehyde in the practical applications. Thus, it was possible that aldehyde was used as the starting reactant to synthesize ethers through cascade reactions of MPV reduction and etherification reactions. The synthesis of methoxybenzyl butyl ether from methoxy benzaldehyde and 2‐butanol was carried out with the catalysts of Sn‐ and Zr‐Beta zeolites. These catalysts exhibited highly active and selective performance toward targeted ether products.^[^ ^163^ ^]^ One‐step synthesized Zr‐Al‐Beta without the dealuminated procedure acted as a bifunctional catalyst to proceed with the reduction‐etherification of cinnamaldehyde (CAL) to 1‐cinnamyl 2‐propyl ether (CPE) in isopropanol.^[^ ^110^ ^]^ It showed higher catalytic performance and water‐tolerance ability than dealumination‐derived Zr‐De‐Al‐Beta, which was attributed to the contribution of Lewis acidic open Zr sites, the Brønsted acidic framework Al sites, and relatively high hydrophobicity. CPE was produced over Al‐free Zr‐De‐Al‐Beta catalyst mainly with Lewis acidity, but its yield was remarkably lower than Zr‐Al‐Beta. This indicated that Lewis acid also can function in the etherification process, however, its efficiency was much lower than that of Brønsted acid.^[^ ^110^, ^241^ ^]^ Anethole as a kind of flavor was produced from a green route via MPV reduction and dehydration of 4‐methoxypropiophenone over Zr‐MSU‐3 material, affording 91% anethole yield with a cis/trans isomer ratio of 8:92.^[^ ^129^ ^]^

The etherification of furfural or HMF with alcohol is a promising route for producing biodiesel components due to its high miscibility and energy density.^[^ ^242^ ^]^ Selectively converting HMF to 2,5‐bis(alkoxymethyl)furan was realized over Lewis acidic Sn‐ and Zr‐Beta zeolite, where intermediate 2,5‐bis(hydroxymethyl)furan (BHMF) was derived from the MPV reduction of HMF and further etherification of BHMF was required to produce ether with above 80% yield.^[^ ^243^ ^]^ As for Sn‐Beta zeolite catalyst, MPV reduction of HMF to BHMF was the rate‐limiting step. Besides, different alcohols can be utilized to gain various ethers with adjustable molecular mass from C10 to C14.^[^ ^243^ ^]^

A similar reaction was performed for the synthesis of (butoxy)methylfuran (BMF) from furfural over Sn‐Al‐Beta zeolite in a continuous mode.^[^ ^244^ ^]^ After 100 h on stream, a high selectivity of above 75% can still be kept for optimal Sn‐Al‐Beta with 2 wt.% Sn and 0.5 wt.% Al ions.^[^ ^244^ ^]^ Raw Zr‐Al‐Beta zeolite undecorated by alkali metal ions demonstrated a catalytic effect for the produce of furfuryl isopropyl ether (FPE) between furfural and isopropanol, which resulted in 29.5% FPE yield at the furfural conversion of 32.7% with the temperature of 120 °C for 3 h.^[^ ^111^ ^]^ In this literature, furfural alcohol was the desired product and FPE was deemed as a byproduct. This catalytic result was just a compared data and the optimal catalytic performance for the synthesis of FPE was unknown due to the absence of an optimal process.^[^ ^111^ ^]^

*α*‐Hydroxy acid esters are useful for the synthesis of functional polyester, which can be obtained from the transformation of tetrose sugars over Lewis acid catalysts.^[^ ^245^ ^]^ Over microporous Sn‐Beta or Sn‐MFI zeolites, the MMHB yield was not ideal due to the sterical restriction.^[^ ^245^ ^]^ Sn‐Al‐MFI with micro‐/mesoporosity can robustly perform the conversion of macromolecule glucose to 5‐(ethoxymethyl)furfural in ethanol, giving 44% targeted product yield.^[^ ^246^ ^]^ With respect to this reaction, it suffered from three tandem reactions including the isomerization of glucose to fructose over Lewis active sites, dehydration of fructose to HMF over Brønsted acid sites, and thereafter etherification of HMF to 5‐(ethoxymethyl)furfural also on Brønsted acid sites.^[^ ^246^ ^]^ The partial catalytic applications of Sn/Zr‐zeolites as biomass catalysis are exhibited in **Table** **4**.

**Table 4:** The catalytic applications of Sn/Zr‐zeolites as biomass catalysis.

Catalyst	Substrate	Desired product	Solvent	T [°C]	Time [h]	Conv. [%]	Sel. [%]	References
Sn‐MFI	glucose	fructose	water	90	12	31.4	87.1	[20]
Sn‐MWW	glucose	methyl lactate	methanol	160	20	>99	44.0	[32]
Zr‐Beta	cinnamaldehyde	CA	isopropanol	82	5	97.3	91.4	[110]
Zr‐Beta	furfural	furfural alcohol	isopropanol	120	3	97.2	97.7	[111]
Sn‐Beta	glucose	5‐HMF	DMSO	160	4	60.0	37.3	[122]
Sn‐Beta	glucose	HMF	DMSO	160	4	60	37	[122]
Zr‐Beta	furfural	GVL	2‐propanol	120	24	>99	95.0	[143a]
Sn‐Beta	fructose	methyl lactate	water	160	16	>99	54.0	[180]
Sn‐Beta	mannose	methyl lactate	methanol	160	16	96	47	[180]
Sn‐Beta	turanose	methyl lactate	methanol	160	44	72	26	[180]
Sn‐Beta	lyxose	methyl lactate	methanol	160	16	96	39	[180]
Sn‐Beta	glucose	fructose	water	110	0.5	55	32	[198]
Sn‐Beta	xylose	furfural	water	110	3	84	14	[199]
Sn‐Beta	xylose	xylulose	water	110	1	90.0	90.0	[200]
Sn‐Beta	glucose	fructose	CH_3_OH	140	30	90.0	41.8	[202]
Sn‐Beta	Starch	HMF	THF/NaCl‐H_2_O	180	1.16	75	52	[212]
Sn‐Beta	glucose	HMF	THF/NaCl‐H_2_O	180	1.16	79	57	[212]
Sn‐Beta	DHA	methyl lactate	methanol	80	24	>99	>99	[220a]
Sn‐Beta	DHA	lactic acid	water	125	24	>99	90	[220c]
Sn‐Beta	GLA	methyl lactate	methanol	80	24	>99	>99	[220a]
Sn‐Beta	GLA	lactic acid	water	125	24	>99	90	[220a]
Sn‐Beta	xylose	xylulose	water	90	0.5	81	24	[222]
Sn‐Beta	glucose	methyl lactate	methanol	160	10	>99	58.0	[225]
Zr‐Beta	HMFA	MMBC	ethylene	190	6	26.0	21.0	[229]
Sn‐Beta	MMFC	MMBC	dioxane	190	2	28	13	[229]
Sn‐Beta	2‐methylfuran	toluene	ethylene	250	8	>90	>70	[235]
Zr‐HY	furfural	GVL	2‐pentanol	120	5	95.0	85.0	[236c]
HPA/Zr‐Beta	furfural	GVL	2‐propanol	160	24	100	54.3	[239]

CA: cinnamyl alcohol; HMFA: 5‐(hydroxymethyl)furoic acid; MMBC: methyl 4‐(methoxymethyl) benzenecarboxylate; DMSO:

dimethylsulfoxide; THF: tetrahydrofuran; DHA: dihydroxyacetone; GLA: glyceraldehyde; MMFC: methyl 5‐(methoxymethyl)

furan‐2‐carboxylat; MMBC: methyl 4‐(methoxy‐methyl)‐benzenecarboxylate; HMF: 5‐hydroxymethylfurfural. GVL: γ‐

valerolactone.

### 3.4 Sn‐ and Zr‐Zeolites as the Catalysis Support

Sn/Zr‐zeolites have become generalists for catalysis applications and exhibited fascinating catalytic performance in the fields of redox, Lewis acid, and biomass catalysis. Apart from these, they also can be employed as catalysis support to load metal species to construct multifunctional catalysts from the consideration of their intrinsic Lewis acid property, confined effect, and metal‐support interaction, which have been emerging as major players in the catalytic applications of ethanol to butadiene, propane dehydrogenation, hydrogenation, oxidation and so on.

#### 3.4.1 Ethanol to Butadiene

The conversion of ethanol to butadiene (ETB) is an appealing reaction due to the importance of butadiene as a polymer building block.^[^ ^247^ ^]^ It is generally accepted that the ETB reaction contained the following five steps, I) forming acetaldehyde from ethanol dehydrogenation; II) generating acetaldol from acetaldehyde via aldol condensation; III) forming crotonaldehyde from acetaldol dehydration; IV) forming crotonyl alcohol from crotonaldehyde via MPV reduction; V) the dehydration of crotonyl alcohol to butadiene. Reaction I, II, and IV were considered the pivotal steps, while the dehydration steps of III and V were easily performed.^[^ ^248^ ^]^ Ivanova et al. found that Ag metal was a very efficient catalyst for ethanol dehydrogenation (step I).^[^ ^249^ ^]^ From Sections 3.1 and 3.2, it was known that Sn/Zr‐zeolites with Lewis acidity were active and selective catalysts for aldol condensation (step II) and MPV reduction (step IV). Therefore, the combination of Ag metal sites and zeolite‐based Lewis acid sites in one catalyst was proposed.^[^ ^248^, ^249^ ^]^

In 2015, Sushkevich et al. hydrothermally synthesized Zr‐Beta as the support of metallic Ag.^[^ ^6b^ ^]^ Authors mainly elucidated the effect of Zr‐zeolites on the catalytic activity, which was sequenced in the following order: Ag/Zr‐Beta > Ag/ZrO_2_/SiO_2_. This activity order was positively correlated with the amounts of Lewis acid within catalysts. Ag/Zr‐Beta with 1.0 wt.% Ag and 1.1 wt.% Zr demonstrated the best performance in terms of 47.9% ethanol conversion and 55.6% butadiene selectivity.^[^ ^6b^ ^]^ One year later, the same group changed the synthesis method of Zr‐Beta zeolite from conventional hydrothermal synthesis to dealuminated‐derived liquid grafting in order to produce more open Zr sites.^[^ ^250^ ^]^ The amounts of open Zr sites were not correlated with the Al contents in the starting Al‐Beta, conversely which was tightly related to the crystal size. The smaller crystal size led to higher open Zr sites, further resulting in higher contents of Lewis acidity. Under the same Ag loading of 1 wt.%, the initial reaction rate over the Ag/Zr‐Beta catalyst was linearly correlated with the open sites of Zr species, which presented significant superiority compared to that over traditionally hydrothermally synthesized Ag/Zr‐Beta. The optimal Ag/Zr‐Beta zeolite catalyst possessed the uppermost productivity of 0.58 g g^−1^ h^−1^ with a selectivity of close to 60%.^[^ ^250^ ^]^ Subsequently, they further confirmed the leading role of open Zr sites within Ag/Zr‐Beta zeolite catalyst for the synthesis of butadiene from ethanol using FT‐IR spectroscopy and DFT calculations.^[^ ^56c^ ^]^ The higher catalytic performance of open Zr sites was contributed by their convenient accessibility and higher strength of Lewis acidity.^[^ ^56c^ ^]^ In 2022, Zhang et al. utilized computational techniques to shed light on the reaction mechanism of the ETB process over a Zr‐Beta‐based catalyst.^[^ ^6a^ ^]^ The results showed that MPV reduction was the rate‐limiting step during the five basic steps. The energy barrier of this proton transfer step for open Zr sites (0.78 eV) was much lower than that (1.79 eV) for closed Zr sites. Isolated and closed Zr sites possessed comparable energy barriers to the zirconium oxide in this process. Consequently, the catalytic performance of open Zr sites within Zr‐Beta zeolite was superior to that of closed Zr sites and zirconium oxides in the ETB conversions.^[^ ^6a^ ^]^

Kurmach et al. used a Gemini‐type surfactant as OSDA to hydrothermally synthesize hierarchical Zr‐MTW zeolites as the host of metallic Cu to design a Cu/Zr‐MTW catalyst.^[^ ^251^ ^]^ Zr‐MTW zeolite had a micro‐mesoporous structure with a mesoporous diameter of ≈6 nm. Additionally, the acid‐base property of Zr‐MTW was associated with the concentration of fluoride in the synthesis gels. Cu‐doped Zr‐MTW (Cu/Zr‐MTW) catalyst was evaluated in ETB reaction, whose productivity for the desired product of butadiene was linearly correlated with the concentration of Lewis acid sites. The best‐performance with the ethanol conversion of 81% and butadiene selectivity of 68% was achieved over the Cu/Zr‐MTW catalyst.^[^ ^251^ ^]^

#### 3.4.2 Propane Dehydrogenation

Propene is one of the most significant feedstocks in the field of chemical industry, which is produced in the processes of fluid catalytic cracking and naphtha cracking.^[^ ^252^ ^]^ Facing the exhaustion of fossil fuels and the increasing demand for propene, alternative novel methods are proposed, including methanol‐to‐olefin, Fischer–Tropsch, and propane dehydrogenation (PDH). The abundant light alkanes from the shale gas make the PDH technique a promising route for replacing the petroleum‐based propene production process.^[^ ^253^ ^]^ Apart from the obvious catalysis effect of Sn‐zeolites for PDH reactions (see Section 3.2.4), using them as catalysis support is also an effective strategy for establishing robust catalysts oriented to PDH reaction. The partial catalytic applications of Sn/Zr‐zeolites as catalysis support are illustrated in **Table** **5**.

**Table 5:** The catalytic applications of Sn/Zr‐zeolites as catalysis support.

Catalyst	Substrate	Desired product	Solvent	T [°C]	Time [h]	Conv. [%]	Sel. [%]	References
Ag/Zr‐Beta	ethanol	butadiene	/	600	5	45.2	58.1	[6a]
Ag/Zr‐Beta	ethanol	butadiene	/	600	3	48.0	56.0	[6b]
Ag/Zr‐S‐1	styrene	SO	acetonitrile	80	8	80.0	84.0	[24]
Ag/Zr‐Beta	ethanol	butadiene	/	320	5	30.0	64.0	[56c]
Al,Sn‐CHA	NO_x_	N_2_	/	900	4	37.0	>99	[76]
Ag/Zr‐Beta	ethanol	butadiene	/	320	3	15.5	67.1	[247a]
Zr‐MTW	ethanol	butadiene	/	375	3	34.0	53.0	[251]
PtNa/Sn‐ZSM‐5	propane	propylene	/	590	3	41.7	95.3	[254]
PtNa/Sn‐ZSM‐5	propane	propylene	/	590	3	41.7	95.3	[254]
Pt/Sn‐Si‐Beta	propane	propylene	/	550	5	27.1	>99	[255]
Pt/Sn‐Beta	propane	propylene	/	570	24	45.8	99.0	[256]
Pt/Sn‐Y	propane	propylene	/	570	48	44.1	96.9	[256]
Pt‐Sn‐Beta‐R	propane	propylene	/	570	250	90.0	99.0	[257]
Ni/Zr‐Beta	citronellal	menthol	*tert*‐butanol	80	22	96.0	96.0	[258]
Ni/Zr‐Beta	citral	menthol	*tert*‐butanol	80	22	100	89.0	[259]
Rh/Zr‐Beta	citral	menthol	*tert*‐butanol	80	229	100	86.0	[259]
Rh/Zr‐Beta	PTBP	alcohol	2‐propanol	80	4	100	100	[260]
Pt/Sn‐Beta	citral	alcohol	2‐propanol	80	4	92.0	87.0	[262]
Pt/Sn‐MFI	glycerol	LA	water	100	24	89.8	80.5	[263]
Pt/Sn‐MFI	PyA	AA	water	100	4	22.3	94.7	[263]
Au/DeAl‐Sn‐Beta	acetol	LA	water	120	3	93.4	73.2	[264]

SO: styrene oxide; PTBP: 4‐*tert*‐butylphenol; LA: lactic acid; PyA: pyruvic acid; AA: acetic acid.

**Figure 19:**
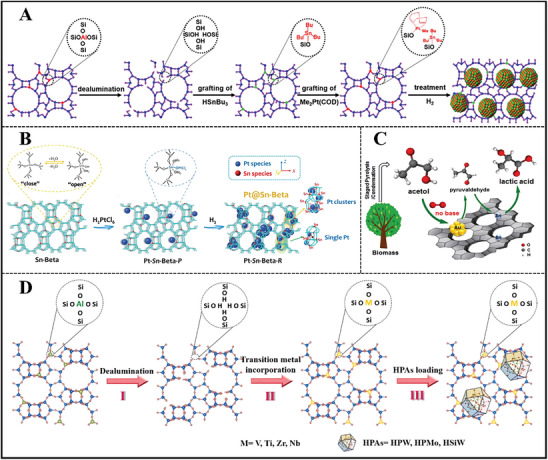
A) Synthesis procedure for Pt/Sn‐Beta catalysts with different Sn/Pt ratios. Reproduced with permission.^[^ ^256^ ^]^ Copyright 2020, American Chemical Society. B) Schematic representation of the proposed synthetic route for Pt single atoms and subnanometric clusters encapsulated in Sn‐Beta (Pt1&Ptn@Sn‐Beta). Reproduced with permission.^[^ ^257^ ^]^ Copyright 2021, Elsevier. C) One‐pot production of Lactic acid from acetol over dealuminated Sn‐Beta supported gold catalyst. Reproduced with permission.^[^ ^264^ ^]^ Copyright 2017, American Chemical Society. D) Schematic illustration of the preparation process of yHPAs‐xM/SiBeta. Reproduced with permission.^[^ ^265^ ^]^ Copyright 2021, Elsevier.

In 2011, Sn‐modified ZSM‐5 was used as the support for the Pt catalyst in the PDH reaction, where partial Sn species existed in the zeolite framework.^[^ ^254^ ^]^ The framework‐substituted Sn altered the interfacial property between Pt and ZSM‐5 support, facilitating hydrogen spillover and further the reduction of Pt. This PtNa/Sn‐ZSM‐5 catalyst demonstrated excellent behavior with 41.7% propane conversion and 95.3% propene selectivity at 590 °C.^[^ ^254^ ^]^ In 2019, Wang et al. took advantage of the strong interaction between Pt and tetrahedrally coordinated Sn species within Beta zeolite framework to establish Pt/Sn‐Beta catalyst with ultra‐small Pt nanoclusters in a size of 1.6 nm for non‐oxidative PDH reaction.^[^ ^255^ ^]^ A high dispersion of Pt species was observed for the Pt/Sn‐Beta sample. This catalyst resulted in an excellent propene formation rate of 2.3 mol g^−1^_Pt_ h^−1^ with a propene selectivity of above 99% at 550 °C. Good stability and regenerated performance were exhibited due to coke‐ and sinter‐resistant features derived from the strong interaction of Pt and framework Sn species.^[^ ^255^ ^]^ One year later, Xu et al. also designed a Pt/Sn‐Beta catalyst via a similar method for PDH.^[^ ^256^ ^]^ The big difference was the synthesis of Sn‐Beta zeolite via a two‐step post‐synthesized method. The Sn source in the former literature was SnCl_4_ˑ5H_2_O and it was tributyltin hydride (Bu_3_SnH) in this paper. Characterizations indicated that there were three types of Sn species within Pt/Sn‐Beta zeolites, (structure A) tetrahedrally coordinated Sn(IV) bonding to the zeolite framework through four Si‐O‐Sn linkages; (structure B) twofold coordinated Sn(II) liked to the zeolite framework through two Si‐O‐Sn linkages; (structure C) metallic Sn within Pt‐Sn alloy. In structure B, the unsaturated Sn(II) species can act as linking sites to anchor Pt nanoclusters. Besides, structure B was the main form of Sn species among these three structures and correspondingly, Pt clusters were fixed at the isolated Sn(II) sites in the zeolite framework. This Pt/Sn‐Beta catalyst possessed extraordinary performance with initial propane conversion of 50%, propene selectivity of > 99%, TOF of 114 s^−1^, and deactivation rate of 0.006 h^−1^, along with good regenerated ability (**Figure** **19**A).^[^ ^256^ ^]^

In 2021, Wu's group also designed a Pt/Sn‐Beta catalyst for the PDH process.^[^ ^257^ ^]^ Herein, Sn‐Beta zeolite was hydrothermally synthesized by structural reconstruction within a short time. Subsequently, Sn‐Beta zeolite was impregnated in H_2_PtCl_6_ solution (denoted as Pt‐Sn‐Beta‐P) and then reduced under H_2_ flow at 500 °C, affording Pt‐Sn‐Beta‐R catalysts. For the Pt‐Sn‐Beta‐P sample, Sn existed in the form of (Si‐O)_3_(H_2_O)_2_Sn^IV^‐(O)‐[PtCl_5_]^−^ and after reduction, (Si‐O)_2_Sn^II^‐(O)‐Pt_n(n = 1–15)_ structure was distinguished by ^119^Sn MAS NMR, XAFS and in situ XPS and XRD spectra. No Pt nanoparticles and Pt_x_Sn_y_ alloy were detected in the resultant Pt‐Sn‐Beta‐R samples. That is, subnanometric Pt clusters were confined within Sn‐Beta zeolite with the assistance of strong interaction between the host of framework Sn species with Beta zeolite and the guest of Pt species. The (Si‐O)_2_Sn^II^‐(O)‐Pt_n(n = 1–15)_ structure served as efficient active sites to steer PDH reaction with low‐temperature activity (47% propane conversion at 500 °C), high propene selectivity of above 99% and good stability (Figure 19B).^[^ ^257^ ^]^

#### 3.4.3 Hydrogenation

The hydrogenation reaction proceeds through the combination of metal and Sn/Zr‐zeolites, which is mainly distinguished into cascade and support‐enhanced reactions. The metal and isolated Sn^4+^/Zr^4+^ within Sn/Zr‐zeolites serve as individual catalytically active sites in the former reaction, which play roles of hydrogenation and Lewis acid catalysis, respectively. For the latter reaction, the presence of Sn/Zr‐zeolites supports the benefits of the hydrogenation of metal species such as through increasing metal dispersion, decreasing the size of metal particles, enhancing the adsorption and activation of guest reactants, and so on.

Menthol is extensively employed in pharmaceuticals, toothpaste, cigarettes, and chewing gum.^[^ ^258^ ^]^ One‐pot synthesis of menthol from (±)‐citronellal was accomplished using a bifunctional Ni/Zr‐Beta catalyst, where isopulegols were intermediates. This reaction suffered from two detailed steps, the cyclization of citronellal to isopulegols (catalyzed by Zr‐Beta zeolite) and the hydrogenation of isopulegols to menthol (catalyzed by Ni species). Ni/Zr‐Beta catalyst afforded a remarkable diastereoselectivity of 90–94% and a high menthol yield of 86–97%.^[^ ^258^ ^]^ The yield and diastereoselectivity of menthol reached up to 95% and 94% under a low pressure of 0.2–2 MPa by minimizing the rates of side reactions.^[^ ^259^ ^]^ The combination of hydrogenation and MPV reduction was attempted for the hydrogenation of 4‐*tert*‐butylphenol and *p*‐cresol over Rh/Zr‐Beta catalysts.^[^ ^259^ ^]^ The usage of Zr‐Beta zeolite reduced the intermediate of 4‐alkylcyclohexanone via the highly stereoselective MPV reduction over Lewis acid sites. The hydrogenation of 4‐tert‐butylphenol and *p*‐cresol to cis‐alcohols was 95% and 89% stereoselectivity, respectively, over 0.5% Rh/Zr‐Beta catalyst.^[^ ^260^ ^]^

For hydrogenation of C═O group, Lewis acid sites in the vicinity of the metal particle are beneficial for the activation of the C═O bond.^[^ ^261^ ^]^ Therefore, a Pt/Sn‐Beta catalyst was designated for the selective hydrogenation of *α,β*‐unsaturated aldehydes.^[^ ^262^ ^]^ Additionally, the presence of framework Sn^4+^ sites in Sn‐Beta zeolite enabled the size of Pt particle low to 0.5–2.5 nm for Pt/Sn‐Beta sample compared to Pt/Beta (a mean size of 5.1 nm), due to the stabilized effect of isolated Sn^4+^ to Pt species. This Pt/Sn‐Beta catalyst exhibited exceptional activity and selectivity in the conversion of *α,β*‐unsaturated citral to unsaturated alcohol by activating the C═O bond and thereby enhancing its hydrogenation rate.^[^ ^262^ ^]^

#### 3.4.4 Other Reactions

Employing Sn/Zr‐zeolites as catalysis support is also an effective strategy for designing and preparing productive catalysts in other reactions. Similar to the hydrogenation reaction (Section 3.4.3), cascade and support‐enhanced reactions are defined for identification. As an example of a cascade reaction, the synthesis of lactic acid started from abundant glycerol (a byproduct of biodiesel production) instead of DHA and GLA over a Pt/Sn‐MFI catalyst.^[^ ^263^ ^]^ This reaction was driven in a tandem reaction route that Pt and Sn‐MFI were responsible for the selective oxidation of glycerol to DHA/GLA and isomerization of DHA/GLA to lactic acid. Pt/Sn‐MFI possessed outstanding catalytic performance with 89.8% glycerol conversion and 80.5% lactic acid selectivity under base‐free conditions.^[^ ^263^ ^]^ A bifunctional Au/Sn‐Beta catalyst was also prepared for the conversion of acetol to lactic acid via a cascade reaction, where pyruvaldehyde was detected as the linking intermediate (Figure 19C).^[^ ^264^ ^]^ One pot conversion of furfural to GVL needs the assistance of Brønsted and Lewis acid sites (see Section 3.3.5). Heteropolyacid (HPA) was anchored on Zr‐Beta zeolite to construct bifunctional HPA/Zr‐Beta zeolite, where HPA and Zr‐Beta played the role of Brønsted and Lewis acid sites, respectively.^[^ ^239^ ^]^ Phosphotungstic acid supported Zr‐Beta catalyst exhibited ≈70% GVL yield at 160 °C for 24 h.^[^ ^239^ ^]^

With respect to support‐enhanced reactions, nanocrystalline Zr‐MFI was hydrothermally synthesized by introducing amphiphilic organosilane in the synthesis gels, which was used as the support to load silver nanoparticles.^[^ ^24^ ^]^ This Ag/Zr‐MFI catalyst exhibited higher catalytic performance than Zr‐MFI and even well‐known TS‐1 zeolite in the epoxidation of styrene due to the cooperative relationship between Ag and Zr‐MFI. Authors claimed that the large ionic radius and electronegative of Zr^4+^ resulted in the high polarizability of Zr─O bond within Zr‐MFI zeolite, which then can activate TBHP efficiently. The Ag nanoparticle can also activate TBHP, forming Ag‐O^−^ species. Both the activated oxygen species reacted with olefin to generate epoxide.^[^ ^24^ ^]^ HPW was anchored heteroatoms‐ containing (V, Ti, Nb, and Zr) Beta zeolites for oxidative desulfurization (ODS), in which the synergistic effect between Beta‐type metallosilicate zeolites and HPW led to form abundant peroxometallate intermediate complexes with excellent oxidized ability toward ODS reaction (Figure 19D).^[^ ^265^ ^]^

Heteroatom‐containing CHA zeolites ([Al, M]‐CHA, M = Fe, Ga, Sn) were hydrothermally synthesized by interzeolite transformation of [Al, M]‐FAU zeolite, which were used as the support of Cu species for the selective catalytic reduction (SCR) of NO_x_ by NH_3_ (NH_3_‐SCR).^[^ ^76^ ^]^ A difference in NH_3_ conversion effectiveness at low temperatures was observed. Cu/[Al, Ga]‐CHA exhibited nearly 100% conversion of NH_3_ at 150 °C, while Cu/[Al, Sn]‐CHA catalyst had excellent stability after the hydrothermal treatment at 900 °C.^[^ ^76^ ^]^ Based on CHA crystal structure collapse at 1050 °C, the thermal stability of Sn, Al‐CHA was deemed to be greater than that of Al, Fe‐CHA, and Al, Ga‐CHA, although its relative crystallinity was less than that of Al‐CHA at 600–950 °C. ^[^ ^76^ ^]^ Wang et al. investigated diffusion behaviors of dibenzothiophene and 4,6‐dimethyldibenzothiophene over three mesoporous silica‐based catalysts (iMo/Zr‐FDU‐12 and NiMo/Zr‐MCFs) in the process of diesel hydrodesulfurization.^[^ ^266^ ^]^ The effective diffusion coefficient was ordered in the following sequence for both DBT and 4,6‐DMDBT, NiMo/Zr‐MCFs > NiMo/Zr‐FDU‐12. Reaction kinetic revealed that the diffusion limitation was greatly relieved when the pore size was above 25 times higher than the kinetic diameter of sulfur‐containing organics.^[^ ^266^ ^]^

## 4 Conclusion and Perspective

Sn/Zr zeolites are increasingly paid attention in the field of scientific and industrial manufacturing communities, which have been becoming superstars among metallosilicate catalysis followed by titanosilicate zeolites. The following conclusions, challenges, and future directions accompany their synthesis and catalytic applications.

1. Both bottom‐up and top‐down methodologies are employed to synthesize Sn/Zr‐containing zeolites successfully. The bottom‐up approach is mainly comprised of hydrothermal synthesis, dry‐gel conversion, interzeolite transformation, and structural reconstruction, which facilitates the introduction of Sn and/or Zr ions with tetrahedral coordination into the zeolite framework. As for fluoride‐assisted hydrothermal synthesis, structural reconstruction, and interzeolite transformation strategy, the obtained Sn/Zr‐zeolites generally exhibit fewer crystal defect sites and excellent hydrophobicity, which are beneficial for catalytic applications in aqueous media, in particular for biomass conversion. In addition, fluoride may also cause environmental problems. Moreover, the added metal ions in the synthesis gel prohibit the crystallization and limit the isolated framework metal incorporation, causing a long synthesis period. Based on the novel idea of efficient utilization of structural building units for zeolite nucleation and crystal growth, structural reconstruction and interzeolite transformation strategies are developed. These two methods are efficient in decreasing crystal size, shortening crystallization time, increasing amounts of framework metal ions, and enhancing framework hydrophobicity. Nonetheless, the mineralizing agent of fluoride is still necessary in some cases.
2. The top‐down approach is a post‐synthetic strategy. Compared with the bottom‐up synthesis method, this approach requires short crystallization time and the resultant material possesses high framework metal content and relatively small crystal size. First, the high metal contents in the initial mixture resulted in the formation of polymerized Sn oxides easily. This situation can be minimized or even eliminated by optimizing the synthesis parameters and selecting a suitable prepared method. Second, this approach is not universal and only suitable for some given zeolites. The frameworks of pristine zeolite with low Si/Al molar ratio (e.g. LTL, MAZ, EON) collapsed easily once immersed in an acid‐aqueous solution. If Al‐rich precursor‐derived Sn/Zr‐containing zeolite is needed, more advanced dealuminated techniques should be developed in the future. Third, in order to create more silanol nests accommodating metal ions, post‐treatment of well‐crystallized zeolite with acid, alkaline, or fluoride is required. However, metal ions fail to occupy all the dealumination‐derived silanol nests in the following metalation stage, which leads to residual defect sites and improves crystal hydrophilicity. Last but not least, hierarchical Sn/Zr‐zeolites can be synthesized by combining acid and base treatment or using hierarchical aluminosilicates as an initial precursor.
3. Sn/Zr‐zeolites are active and selective catalysts in redox reactions, Lewis acid catalysis, biomass catalysis, and robust catalysis support. Sn/Zr‐zeolite catalysts obtained from various methods possess discrepant catalytic performance for a specific reaction. The synthesis parameters affect the property of the resultant Sn‐Beta, for example, framework hydrophobicity, diffusion ability, Sn species coordination states and distribution, Lewis acidity, etc. Those physicochemical properties likely contribute to significantly different catalytic behavior, irrespective of the similar XRD patterns.
4. The diffusion property, hydrophobicity, and confinement effect are tightly related to catalytic performance sometimes. In general, Sn/Zr‐zeolites with microporous structures impose severe diffusing limitations on bulky substrates. However, mesoporous silicates improve the diffusion of guest organic and accessibility of active sites benefiting from large pore sizes. The confinement effect favors the catalytic activity for some reactions like MPV reduction in the absence of diffusion limitation. In addition, Sn/Zr‐containing zeolite materials synthesized by bottom‐up methods are more hydrophobic than the obtained ones by top‐down approaches, especially with the assistance of fluoride. The excellent hydrophobicity of Sn/Zr‐zeolites increases the material's stability and catalytic activity for the related aqueous systems. Moreover, developing hierarchical Sn/Zr‐zeolites is significant for certain reactions benefiting from the balancing of diffusion property, hydrophobicity, and confinement effect.
5. Sn/Zr‐zeolites with isolated Sn and Zr species in the framework exhibit Lewis acidity. While the amount and strength of Lewis acid sites are discrepant in a certain topology structure. For a specific reaction, the optimal type of metal site and topology structure is various. Besides, zeolite topology is associated with the diffusion property, micro‐environment, and acid strength of metal species, which further affect the catalytic performance. Exploiting novel hosts for Sn/Zr‐zeolites is always inspiring.
6. The general view is that Sn/Zr (IV) species in tetrahedral coordination form are the active centers for the catalytic reaction using stannosilicates/zirconosilicates as Lewis acid catalysts. Compared with framework metals, extra‐framework ones are less active and even inactive. Note that the structure configuration of isolated metal (open sites vs closed sites) is also significantly related to the catalytic activity. For certain reactions, metal active sites with open configurations are demonstrated to become more active than closed ones, including B–V oxidation, MPV reduction, and glucose isomerization. However, these results are verified through utilizing ex situ characterization methods that may not reflect the actual micro‐environment of metal species under reaction conditions. Developing in situ characterizations is significant for an in‐depth understanding of the real relationships of materials synthesis, active site structure, and catalytic performance.
7. The integration of Lewis and Brønsted acidity has attracted considerable research in the field of multiple cascade reactions. Considering the unique coordinated ability of isolated Sn‐derived Lewis acid sites with carbonyl and hydroxyl groups in biomass platform molecules, stannosilicates become more applicable than zirconosilicates in biomass catalysis in most cases. Although the nominal Lewis and Brønsted acid sites are required in these tandem reactions, the amount and strength of Lewis and Brønsted acid are various. The synthesis of biofuel and chemicals from biomass in cascade reactions is an important direction by means of designing multifunctional catalysts and screening process conditions.
8. The deactivated mechanism of Sn‐ and Zr‐zeolites mainly contains metal leaching, fouling, site restructuring, and structure collapse. In an aqueous catalytic system, hydrothermally synthesized zeolites generally demonstrated higher stability than post‐synthetic ones due to the fact that the amorphization phenomenon and modification of the tin structure easily occur over hydrophilic zeolites. Additionally, this deactivation was permanent, and cannot be recovered. In most cases, the stability is improved using organic substance as the solvent, where metal sites are stable in zeolite framework and fouling may be the main reason for deactivation. Regeneration through a simple calcination treatment is capable of completely restoring their catalytic performance. Moreover, the chelating effect of sugars sometimes provokes the metal leaching in zeolites during biomass conversions.
9. Sn/Zr‐zeolites can also be used as support to host metal species to establish multifunctional catalysts from the standpoint of their intrinsic Lewis acid property, metal‐support interaction, and confined effect. The combination of metal and Sn/Zr‐zeolites leads to form cascade and support‐enhanced reactions. Cascade reactions are derived from the respective functions of metal and Sn/Zr‐zeolites. With respect to support‐enhanced reaction, the presence of Sn/Zr‐zeolite supports is beneficial for the performance improvement of metal species such as dispersion, particle size, stability, electronic effect, etc. The proximity of metal and acid sites within this composited catalyst material should be paid attention to in the future.

Every synthesis strategy has its advantages and disadvantages, which are only suitable for a special one or several Sn/Zr‐zeolites. Improving the present synthesis technique and developing a novel methodology for the already existing such as Zr‐Beta or unfeasible zeolite like Sn‐MAZ are still on the way. Moreover, structural reconstruction and interzeolite transformation are inspirational, which build a bridge between conventional hydrothermal synthesis and the post‐synthesized method. Constructing creative synthesis methodologies, new zeolite catalysts, and novel catalytic applications, are three engines to promote the development of the Sn/Zr‐zeolites community. It is well convinced that the recent advance made in synthesis and catalytic applications of Sn/Zr‐zeolites will soon definitely break new ground in metallosilicate catalysis in a rational way.

## Conflict of Interest

The authors declare no conflict of interest.
